# An Entry to Enantioenriched 3,3-Disubstituted Phthalides through Asymmetric Phase-Transfer-Catalyzed γ-Alkylation

**DOI:** 10.1021/acs.joc.0c00880

**Published:** 2020-05-14

**Authors:** Marina Sicignano, Rosaria Schettini, Giovanni Pierri, Maria Leda Marino, Irene Izzo, Francesco De Riccardis, Luca Bernardi, Giorgio Della Sala

**Affiliations:** † Dipartimento di Chimica e Biologia “A. Zambelli”, Universitá degli Studi di Salerno, Via Giovanni Paolo II 132, 84084 Fisciano, SA, Italy; ‡ Department of Industrial Chemistry “Toso Montanari” & INSTM RU Bologna, Alma Mater Studiorum University of Bologna, Viale del Risorgimento 4, 40136 Bologna, Italy

## Abstract

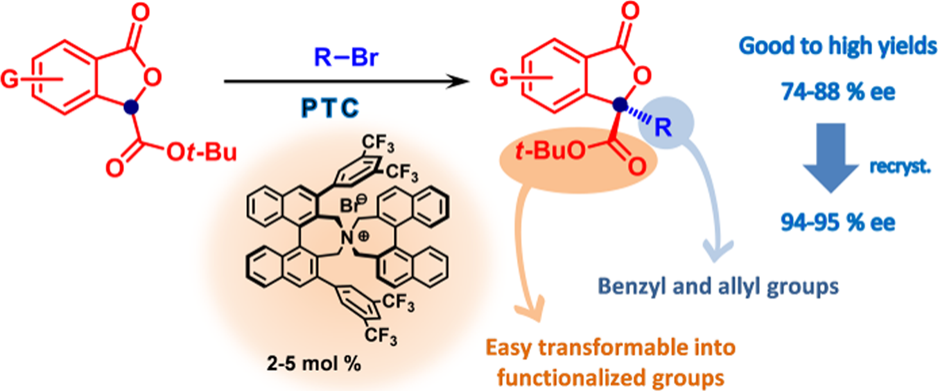

A novel asymmetric phase-transfer-catalyzed γ-alkylation of phthalide 3-carboxylic esters has been developed, giving access to 3,3-disubstituted phthalide derivatives, which present a chiral quaternary γ-carbon in good to excellent yields and good enantioselectivities (74–88% ee). The enantiomeric purity could be substantially enhanced to 94–95% ee by recrystallization. Both electron-withdrawing and electron-releasing substituents are well tolerated on the phthalide core as well as on the aromatic moiety of the alkylating agent. This methodology, enabling the introduction of an unfunctionalized group at the phthalide γ-position, fully complements previously reported organocatalytic strategies involving functionalized electrophiles, thus expanding the scope of accessible 3,3-disubstituted products. The high synthetic value of this asymmetric reaction has been proven by the formal synthesis of the naturally occurring alkaloid (+)-(9*S*,13*R*)-13-hydroxyisocyclocelabenzine.

## Introduction

**Figure 1:**
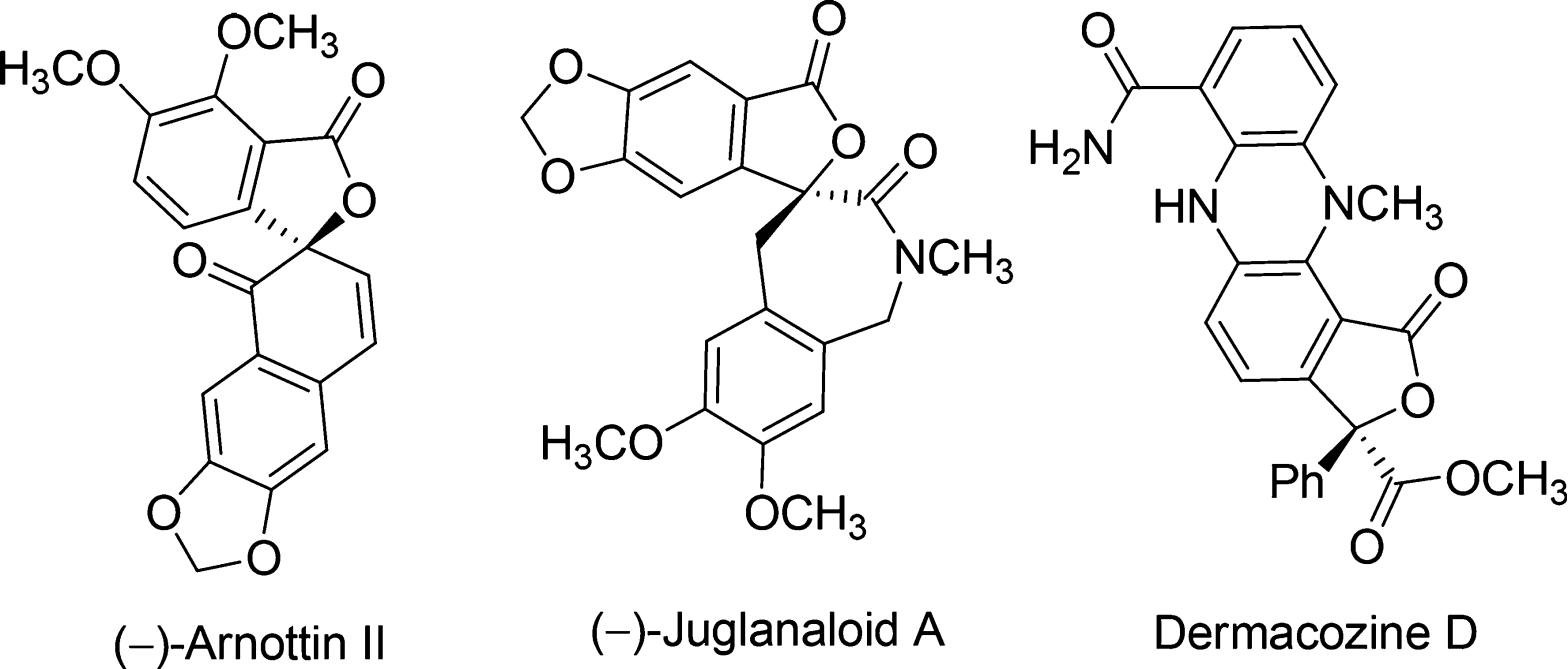
Representative natural 3,3-disubstituted phthalides with a chiral γ-quaternary carbon atom.

The isobenzofuran-1(3*H*)-one core is a ubiquitous pharmacophore incorporated in the structure of a large family of natural products and synthetic analogues, known collectively as phthalides, which display a considerably wide range of useful biological activities.^1,2^ In addition, phthalides are also valuable intermediates for the synthesis of several drugs and naturally occurring compounds such as anthracyclines and other antibacterial and anticancer quinones,^3^ phthalanes,^4^ isocoumarins,^5^ phthalazines,^6^ and others.^5,7^ Despite the conspicuous efforts devoted to the asymmetric syntheses of phthalides, typically performed through the stereocontrolled formation of the lactone ring, the vast majority of methods furnish 3-monosubstituted lactones, whereas enantioenriched 3,3-disubstituted derivatives containing a chiral γ-quaternary carbon atom, also widely represented in nature (Figure 1), remain elusive.^8^

**Figure 2:**
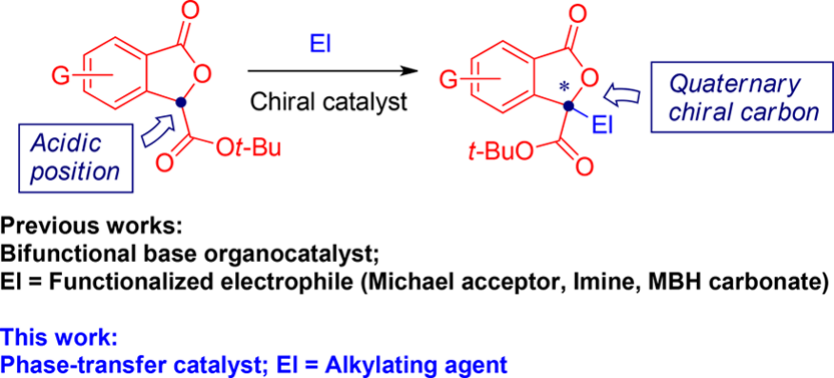
Enantioselective synthesis of 3,3-disubstituted phthalides through direct group insertion at the γ-position.

To this end, the stereocontrolled direct introduction of an electrophilic group at the γ-position of a 3-substituted phthalide has recently emerged as a viable alternative (Figure 2).^9−14^ In this context, γ-activated phthalide esters and nitriles proved to be ideal substrates in reactions catalyzed by bifunctional and polyfunctional base organocatalysts containing a thiourea moiety. Such catalysts, which are tailor-made for functionalized electrophiles capable to interact with hydrogen-bond donor groups, furnished excellent results with imines,^10^ Michael acceptors,^11^ and Morita–Baylis–Hillman carbonates.^12,13^ Remarkably, reactions with unfunctionalized alkylating agents, such as alkyl halides, which lack hydrogen-bond acceptor groups, have never been reported to date in asymmetric nor racemic version. For this transformation, which would significantly expand the scope of accessible 3,3-disubstituted products, the abovementioned bifunctional base organocatalysts do not appear suitable.

It is well known that the most effective organocatalytic strategy to achieve enantioselective alkylation of weakly acidic substrates is asymmetric phase-transfer catalysis.^15^ Although phthalide 3-carboxylic esters meet all the requirements, their application in phase-transfer catalysis has not yet been explored. In this article, we report the development of a novel phase-transfer-catalyzed alkylation of phthalide 3-carboxylic esters, demonstrating the utility of such a process in asymmetric synthesis.

## Results and Discussion

**Figure 3:**
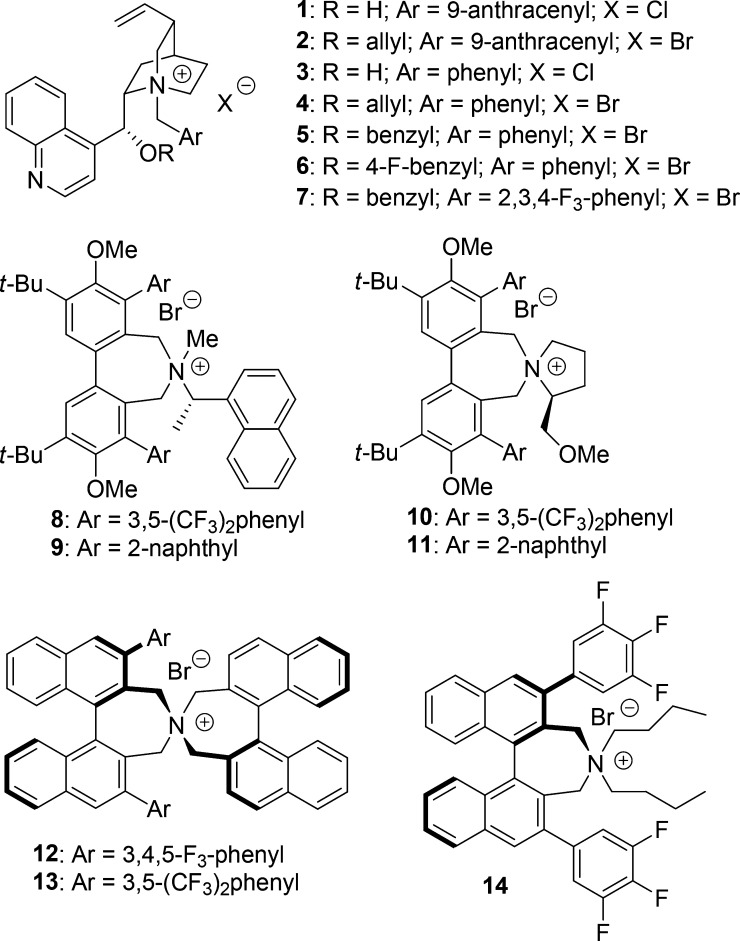
Phase-transfer catalysts screened in the benzylation of **15a**.

Our investigation began with a preliminary screening of chiral phase-transfer catalysts (Figure 3) in the reaction of substrate **15a** with benzyl bromide **16a** (Table 1). In the beginning, various cinchonidinium salts were surveyed in toluene/KOH 50% aq (Table 1, entries 1–7), but although the anticipated product **17aa** was obtained in good yields and very short reaction times in most cases, the enantioselectivities were disappointing, with ee values not exceeding 24%.^16^

**Table 1:**
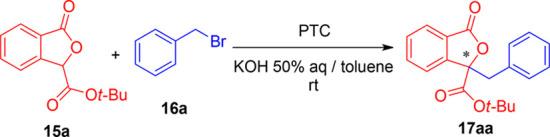
Screening of Catalysts^a^

entry	cat (mol %)	*t* (h)	yield (%)^b^	ee (%) (optical rotation)^c^
1	**1** (10)	0.5	99	20 (−)
2	**2** (10)	1	87	22 (−)
3	**3** (10)	3	41	2 (−)
4	**4** (10)	1	52	6 (−)
5	**5** (10)	0.5	50	18 (+)
6	**6** (10)	0.5	63	22 (+)
7	**7** (10)	1	70	24 (−)
8	**8** (10)	1	70	34 (−)
9	**9** (10)	1	64	16 (−)
10	**10** (10)	1	50	12 (−)
11	**11** (10)	19	70	4 (−)
12	**12** (5)	1	72	56 (+)
13	**13** (5)	0.25	81	70 (+)
14	**14** (5)	0.5	95	24 (+)
15	**13** (2)	0.25	62	43 (+)

a Reaction conditions: **15a** (0.05 mmol), **16a** (0.06 mmol), catalyst (*x* mmol), KOH 50% aq (0.3 mL), toluene (0.5 mL).

b Isolated yields.

c Determined by chiral HPLC.

Lygo’s chiral biphenyl azepinium salts **8–11**(17) also led smoothly to the desired product with low ee values (entries 8–11). A small improvement was observed with derivative **8**. Finally, a striking enhancement of enantioselectivity was achieved with Maruoka’s *N*-spiro *C*_2_-symmetric catalysts **12** and **13**, even when applied at lower (5 mol %) loadings (entries 12–13). A higher reaction rate and enantiomeric excess were achieved with the latter derivative. A poor result was instead observed with *N*,*N*-dibutyl ammonium salt **14** (entry 14). A smaller catalyst amount led to the decline of the enantiomeric excess (entry 15). Attempts to use the corresponding benzyl and ethyl phthalide esters under conditions described in Table 1, entry 13, resulted only in decomposition products.

The X-ray analysis of the major dextrorotatory enantiomer, produced in the reaction catalyzed by (*R*,*R*)-configured catalyst **13**, made it possible to determine its absolute configuration as (*R*).^18^

The effects of the aqueous base and the reaction medium were next studied (Table 2). Bases other than KOH required longer reaction times but led to improved ee values (entries 2–5). Cesium aqueous bases, especially Cs_2_CO_3_ (entry 3), led to the best combination of ee and yield in reasonable reaction times. Cs_2_CO_3_ 50% aq was therefore used in the following runs. Both the reaction rate and stereoselectivity were reduced in CH_2_Cl_2_ (entry 6), whereas good results were observed in ethereal solvents (entries 7–8). However, the best results were generally achieved in aromatic nonpolar solvents (entries 3 and 9–11). Both *p*- and *o*-xylene gave results almost identical to toluene (cf. entries 10–11 with entry 3), suggesting their use as alternative reaction media. However, toluene is easier to be removed. Lower ee values were obtained in more polar solvents such as chlorobenzene and fluorobenzene (entries 12–13). Conducting the reaction in toluene at 0 °C did not yield any enhancement of enantioselectivity (entry 14), whereas at −20 °C, no traces of product were detected, even after 24 h (entry 15).

**Table 2:**
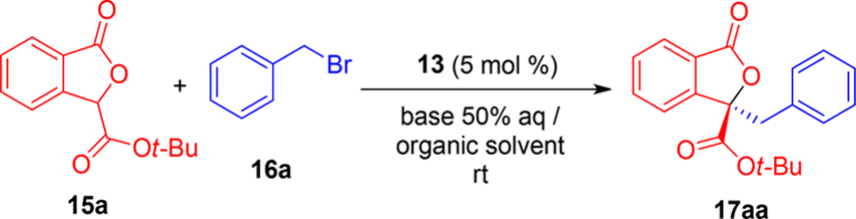
Effect of Base and Solvent^a^

entry	aq base	solvent	*t* (h)	yield (%)^b^	ee (%)^c^
1	KOH	toluene	0.25	81	70
2	CsOH	toluene	1	65	78
3	Cs_2_CO_3_	toluene	4	82	80
4	K_2_CO_3_	toluene	72	40	78
5	K_3_PO_4_	toluene	72	15	70
6	Cs_2_CO_3_	CH_2_Cl_2_	72	73	60
7	Cs_2_CO_3_	Et_2_O	3	78	73
8	Cs_2_CO_3_	MTBE	6	62	71
9	Cs_2_CO_3_	mesitylene	4	73	76
10	Cs_2_CO_3_	*p*-xylene	4	83	80
11	Cs_2_CO_3_	*o*-xylene	4	80	80
12	Cs_2_CO_3_	chlorobenzene	20	75	72
13	Cs_2_CO_3_	fluorobenzene	4	63	60
14^d^	Cs_2_CO_3_	toluene	20	88	78
15^e^	Cs_2_CO_3_	toluene	20		

a Reaction conditions: **15a** (0.05 mmol), **16a** (0.06 mmol), **13** (0.0025 mmol), aqueous base (50% w/w, 0.3 mL), toluene (0.5 mL).

b Isolated yields.

c Determined by chiral HPLC.

d Reaction performed at 0 °C.

e Reaction performed at −20 °C.

**Scheme 1:**
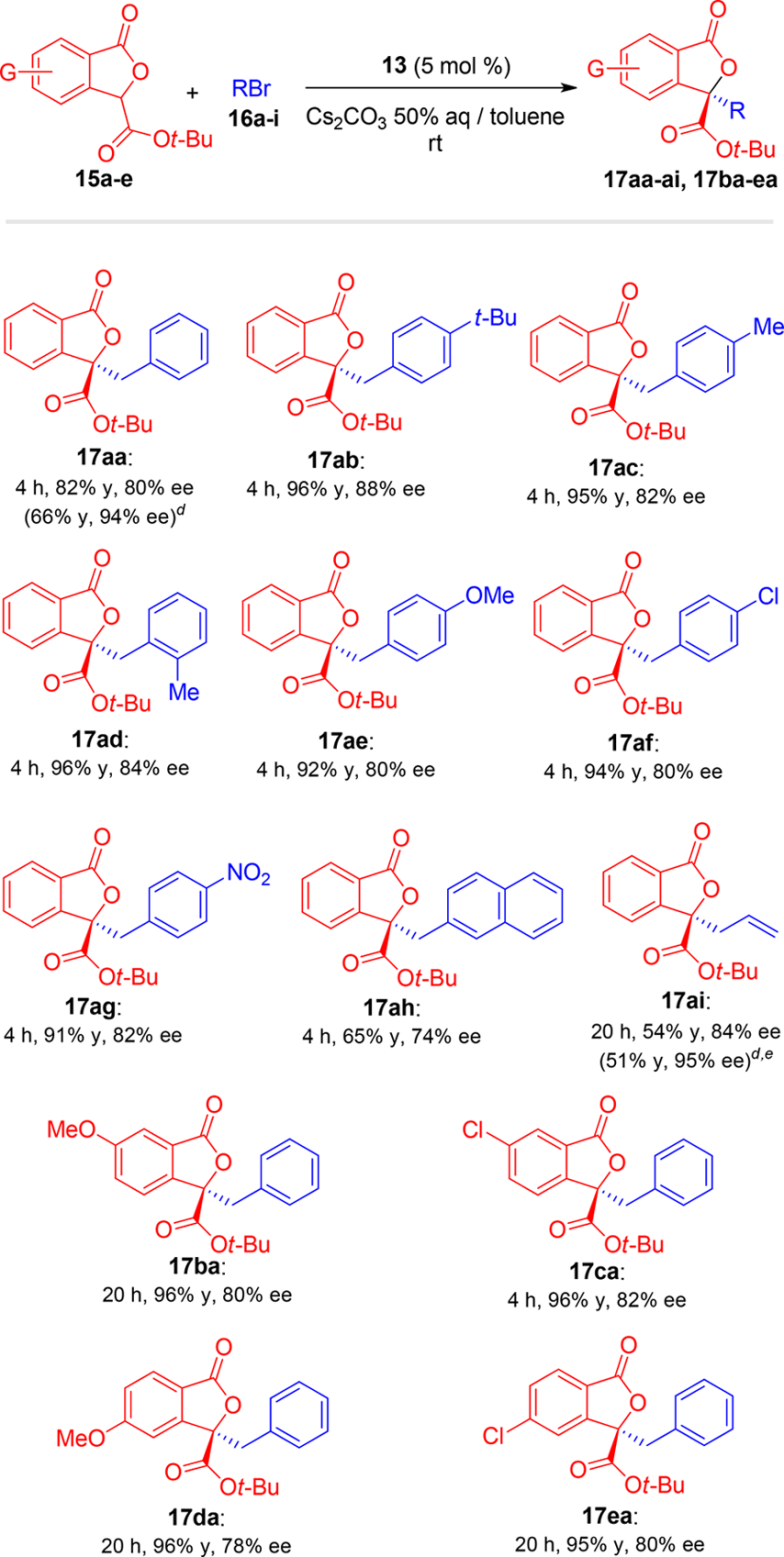
Scope of the Phase-Transfer-Catalyzed Alkylation of Phthalide Esters– The following reaction conditions were applied unless otherwise specified: **15** (0.10 mmol), **16** (0.12 mmol), **13** (0.005) mmol, Cs_2_CO_3_ 50% aq (0.6 mL), toluene (1.0 mL). Isolated yields. ee determined by chiral HPLC. Values in parenthesis refer to the product after recrystallization. Allyl bromide (0.50 mmol) was used.

With the optimized reaction conditions displayed in Table 2, entry 3, we undertook the study of process scope (Scheme 1). Gratefully, uniformly good yields (65–96%) and enantioselectivities (74–88% ee) were achieved with diversely substituted phthalide esters **15a**–**15e** and benzyl bromides **16a**–**16h**. Both electron-withdrawing and electron-releasing substituents were well tolerated at the 5- or 6-position of the phthalide (products **17ba**–**17ea**) as well as on the benzyl moiety (products **17ab**–**17ah**). The presence of an *ortho*-methyl group on the benzyl bromide partner (product **17ad**) did not affect the reactivity or the enantioselectivity. A good result was also achieved in the reaction with allyl bromide (**17ai**, 54% yield, 84% ee).^19^ It is worth noting that no traces of hydrolysis byproducts were detected in all the cases examined. In addition, we were delighted to find that products could be easily enantioenriched up to >90% ee through recrystallization from *n*-hexane. As examples, **17aa** was enantioenriched from 80 to 94% ee, and **17ai** was enantioenriched from 84 to 95% ee, with acceptable overall yields in both cases (66 and 51%, respectively).

During the scale-up optimization of this process we found that good yield and comparable enantioselectivity can be achieved at a 1 mmol scale by using only 2 mol % catalyst **13** (62%, 78% ee for product **17aa**).

**Figure 4:**
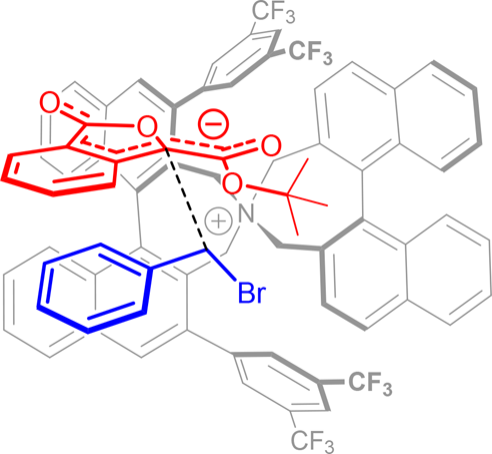
Tentative transition state model for the reaction between phthalide 3-carboxylic ester **15a** and benzyl bromide **16a**, catalyzed by (*R*,*R*)-**13**.

It is reasonable to assume that the observed enantioselectivity comes from a preferential orientation of the substrate anion, generated upon deprotonation of phthalide ester **15**, within the ion pair formed with the *N*-spiro *C*_2_-symmetric cation. Transition state models, based on DFT calculations, have been previously reported for the benzylation of *N*-(diphenylmethylene)glycine *tert*-butyl esters catalyzed by these Maruoka’s ammonium salts. In these models, coulombic interactions as well as nonclassical hydrogen bonds between both reactants and the two catalysts’ enantiotopic fluorinated aromatic groups play a key role in dictating the mutual orientation of the alkylating agent and the substrate anion.^20^ Thus, building on these computed catalyst conformation and interactions with the substrates, it is possible to tentatively sketch a transition state for the alkylation of *tert*-butyl phthalide 3-carboxylate **15a** with benzyl bromide **16a**, catalyzed by (*R*,*R*)-**13** (Figure 4). In this model, reaction occurs at the *Si*-face of the substrate anion, accounting for the high selectivity observed toward the (*R*)-**17aa** product.

**Scheme 2:**
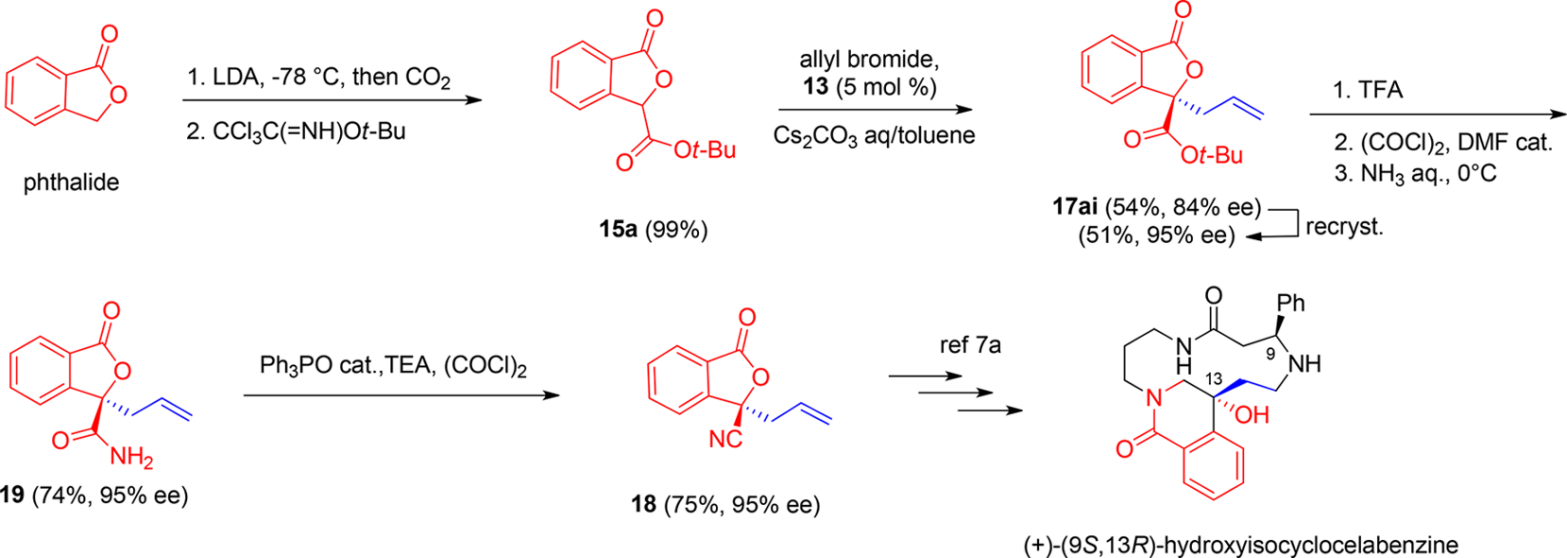
Formal Synthesis of (+)-(9*S*,13*R*)-Hydroxyisocyclocelabenzine

Then, to further stress the synthetic significance of the present methodology in gaining access to new enantioenriched phthalide compounds, we focused on the enantioselective preparation of nitrile (*R*)-**18**, a key intermediate for the synthesis of the naturally occurring spermidine alkaloid (+)-(9*S*,13*R*)-13-hydroxyisocyclocelabenzine (Scheme 2).^7a,21^ It should be noted that the control of the (*R*)-configuration in **18** is an essential requirement to achieve the (13*R*)-configuration in the dihydroisoquinolinone moiety of the target natural product. The original synthesis involved the preparation and use of **18** as a racemate, leading to the target natural product as a mixture of (9*S*,13*R*) and (9*S*,13*S*) diastereomers, which were separated only by tedious repeated flash chromatography.^7a^ Our first synthesis of (*R*)-**18** was accomplished in six steps starting from commercially available parent phthalide, with an overall 28% yield and 95% ee (Scheme 2). The enantioselective allylation of **15a** followed by fractional crystallization afforded **17ai** in 51% yield and 95% ee, as described before. Then, de-*tert*-butylation, synthesis of primary amide **19**, and subsequent dehydration^22^ afforded (*R*)-**18**, a formal precursor of (+)-(9*S*,13*R*)-13-hydroxyisocyclocelabenzine.^23^

## Conclusions

In conclusion, the first asymmetric γ-alkylation of phthalide 3-carboxylic esters has been herein described, affording 3,3-disubstituted products incorporating benzyl and allyl groups with generally high yields and good enantioselectivity. Excellent enantiomeric excesses could be achieved by recrystallization. The present and previous studies^10^ demonstrated that the carboxylic group can be readily manipulated giving access to functionalized 3,3-disubstituted phthalides that were previously unaccessible in enantioenriched form. For example, we developed the enantioselective synthesis of a precursor of (+)-(9*S*,13*R*)-13-hydroxyisocyclocelabenzine. Further synthetic applications of this methodology will be reported by us in due course.

## Experimental Section

### General Remarks

All the chemicals and solvents were purchased from commercial suppliers and used without further purification. Catalysts **1–4** and **12–14** are commercially available, whereas catalysts **5–7**(24) and **8–11**(17a) were prepared following the general procedure described in the literature. 5-Chloro-, 6-chloro-, 5-methoxy-, and 6-methoxyisobenzofuran-1(3*H*)-ones were prepared as described in the literature.^25^ Reactions were monitored by analytical thin-layer chromatography (TLC) on precoated silica gel plates (0.25 mm) and visualized by UV light or by spraying KMnO_4_/ethanol or ninhydrin/ethanol solutions and heating on a hot plate. Flash chromatography was performed on silica gel 60 (particle size: 0.040–0.063 mm). ^1^H and ^13^C NMR spectra were recorded on a Bruker Avance-600 MHz spectrometer and a Bruker Avance-400 MHz spectrometer at room temperature in CDCl_3_, respectively. All the NMR spectra were referenced to residual CHCl_3_ (7.26 ppm, ^1^H; 77.16 ppm, ^13^C). The following abbreviations are used to indicate the multiplicity in NMR spectra: s = singlet; d = doublet; dd = double doublet; t = triplet; bs = broad signal; and m = multiplet. Coupling constants (*J*) are quoted in Hertz. Optical rotations were measured on a Jasco P-2000 digital polarimeter using WI (tungsten-halogen) lamp (λ = 589 nm). Enantiomeric excesses were determined using a CHIRALPAK AS-H column (ϕ 0.46 cm × 25 cm) on a JASCO PU-4180 instrument equipped with a photodiode array detector MD-4015 set at 220 nm. High-resolution mass spectra (HRMS) were acquired using a Bruker solariX XR Fourier transform ion cyclotron resonance mass spectrometer (Bruker Daltonik GmbH, Bremen, Germany) equipped with a 7 T refrigerated actively shielded superconducting magnet and with a LTQ Orbitrap XL Thermo Scientific. The samples were ionized in positive-ion mode using a MALDI or ESI ionization source.

### Synthesis of Substrates

#### *tert*-Butyl 3-Oxo-1,3-dihydroisobenzofuran-1-carboxylate (**15a**)

LDA was freshly prepared by adding a 2.5 M butyllithium solution in hexanes (11 mL, 27.5 mmol) to an anhydrous solution of 0.5 M isopropylamine in THF (55 mL, 27.5 mmol) (28.0 mL) at −78 ° C under a nitrogen atmosphere. The mixture was stirred for 30 min at the same temperature, and then a solution of isobenzofuran-1(3*H*)-one (2.49 g, 18.6 mmol) in anhydrous THF (3.8 mL) was slowly added. The resulting mixture was stirred for 50 min at −78 °C under a nitrogen atmosphere. Then, the reaction vessel was saturated with carbon dioxide (three freeze–pump–thaw cycles followed by connection with a carbon dioxide balloon), and stirring was kept at −78 °C for 2 h. Once reaction was complete, NH_4_Cl saturated aqueous solution (15 mL) was added dropwise, and then THF was removed under reduced pressure. The mixture was basified with Na_2_CO_3_ saturated aqueous solution until pH 9 and washed with AcOEt (2 × 20 mL). The aqueous phase was acidified with concd HCl solution until pH 1, and the product was extracted with AcOEt (3 × 50 mL). The combined organic phases were dried over Na_2_SO_4_ and concentrated under reduced pressure. The resulting crude phthalide 3-carboxylic acid (3.31 g, 18.6 mmol) was dissolved in anhydrous CH_2_Cl_2_ (70 mL), and *tert*-butyl 2,2,2-trichloroacetimidate (3.3 mL, 18.6 mmol) was added. The reaction mixture was stirred for 48 h under a nitrogen atmosphere, then diluted with CH_2_Cl_2_, and centrifuged. The supernatant solution was concentrated under reduced pressure, and the crude residue was purified by flash chromatography (silica gel; petroleum ether/ethyl acetate, 95:5 to 80:20), affording **15a** as a white solid (4.36 g, 99% yield). The characterization data matched those previously reported.^12a^^1^H NMR (400 MHz, CDCl_3_) δ 7.92 (d, *J* = 7.6 Hz, 1H), 7.71 (t, *J* = 7.6 Hz, 1H), 7.64 (d, *J* = 7.6, 1H), 7.58 (t, *J* = 7.6 Hz, 1H), 5.76 (s, 1H), 1.48 (s, 9H).

#### *tert*-Butyl 5-Methoxy-3-oxo-1,3-dihydroisobenzofuran-1-carboxylate (**15b**)

A mixture of 3-methoxybenzoic acid (0.76 g, 5.0 mmol), glyoxylic acid monohydrate (0.92 g, 10 mmol), concd H_2_SO_4_ (0.55 mL, 10 mmol), and glacial acetic acid (20.0 mL) was stirred at 80 °C. After 48 h, the reaction mixture was cooled to room temperature and extracted with AcOEt (3 × 50 mL). The combined organic phases were dried over Na_2_SO_4_ and concentrated under reduced pressure, affording crude phthalide 3-carboxylic acid (0.56 g, 2.7 mmol). This solid was dissolved in anhydrous CH_2_Cl_2_ (10 mL) and treated with *tert*-butyl 2,2,2-trichloroacetimidate (0.59 g, 2.7 mmol) under a nitrogen atmosphere. After stirring for 48 h, the reaction mixture was diluted with CH_2_Cl_2_ and centrifuged. The supernatant solution was concentrated under reduced pressure, and the crude residue was purified by flash chromatography (silica gel; petroleum ether/ethyl acetate, 95:5 to 80:20), affording **15b** as a white solid (0.30 g, 23% yield). mp 89–90 °C. ^1^H NMR (400 MHz, CDCl_3_) δ 7.49 (d, *J* = 8.4 Hz, 1H), 7.30 (bs, 1H), 7.23 (d, *J* = 8.4, 1H), 5.68 (s, 1H), 3.85 (s, 3H), 1.46 (s, 9H). ^13^C{^1^H} NMR (100 MHz, CDCl_3_) δ 169.7, 165.6, 161.3, 136.8, 126.4, 123.2, 123.2, 107.7, 83.7, 77.4, 55.8, 27.8. HRMS (MALDI) *m*/*z*: [M + Na^+^] calcd for C_14_H_16_NaO_5_^+^, 287.0890; found, 287.0892.

#### *tert*-Butyl 5-Chloro-3-oxo-1,3-dihydroisobenzofuran-1-carboxylate (**15c**)

Compound **15c** was prepared following the procedure described for **15a**, starting from 5-chloroisobenzofuran-1(3*H*)-one (3.14 g, 18.6 mmol). Purification of the crude product by flash chromatography (silica gel; petroleum ether/ethyl acetate, 95:5 to 80:20) afforded **15c** as a white solid. Yield: 1.85 g (37%). mp 87–88 °C. ^1^H NMR (400 MHz, CDCl_3_) δ 7.80 (d, *J* = 1.4 Hz, 1H), 7.64 (dd, *J* = 8.2, 1.9 Hz, 1H) 7.57, (d, *J* = 8.2 Hz, 1H), 5.73 (s, 1H), 1.45 (s, 9H). ^13^C{^1^H} NMR (100 MHz, CDCl_3_) δ 168.2, 164.9, 142.7, 136.4, 134.8, 126.8, 125.7, 123.9, 84.4, 77.5, 27.8. HRMS (MALDI) *m*/*z*: [M + H^+^] calcd for C_13_H_14_ClO_4_^+^, 269.0575; found, 269.0586.

#### *tert*-Butyl 6-Methoxy-3-oxo-1,3-dihydroisobenzofuran-1-carboxylate (**15d**)

Compound **15d** was prepared following the procedure described for **15a**, starting from 6-methoxyisobenzofuran-1(3*H*)-one (3.05 g, 18.6 mmol). Purification of the crude product by flash chromatography (silica gel; petroleum ether/ethyl acetate, 95:5 to 80:20) afforded **15d** as a white solid. Yield: 2.36 g (48%). mp 82–81 °C. ^1^H NMR (600 MHz, CDCl_3_) δ 7.81 (d, *J* = 8.6 Hz, 1H), 7.09–7.06 (m, 2H), 5.68 (s, 1H), 3.90 (s, 3H), 1.49 (s, 9H). ^13^C{^1^H} NMR (150 MHz, CDCl_3_) δ 169.4, 165.6, 164.9, 147.3, 127.4, 117.3, 117.2, 106.6, 84.0, 77.2, 55.9, 27.9. HRMS (MALDI) *m*/z: [M + H^+^] calcd for C_14_H_17_O_5_^+^, 265.1071; found, 265.1076.

#### *tert*-Butyl 6-Chloro-3-oxo-1,3-dihydroisobenzofuran-1-carboxylate (**15e**)

Compound **15e** was prepared following the procedure described for **15a**, starting from 6-chloroisobenzofuran-1(3*H*)-one (3.14 g, 18.6 mmol). Purification of the crude product by flash chromatography (silica gel; petroleum ether/ethyl acetate, 95:5 to 80:20) afforded **15e** as a white solid. Yield: 1.80 g (36%). mp 94–95 °C. ^1^H NMR (400 MHz, CDCl_3_) δ 7.83 (d, *J* = 8.2 Hz, 1H), 7.62 (d, *J* = 1.2 Hz, 1H), 7.55 (dd, *J* = 8.2, 1.2 Hz, 1H), 5.72 (s, 1H), 1.49 (s, 9H). ^13^C{^1^H} NMR (100 MHz, CDCl_3_) δ 168.4, 164.8, 146.1, 141.1, 130.8, 127.0, 123.6, 123.0, 84.5, 77.1, 27.9. HRMS (MALDI) *m*/*z*: [M + H^+^] calcd for C_13_H_14_ClO_4_^+^, 269.0575; found, 269.0582.

#### Ethyl 3-Oxo-1,3-dihydroisobenzofuran-1-carboxylate (**15f**)

To a solution of 3-oxo-1,3-dihydroisobenzofuran-1-carboxylic acid, prepared as described above (0.2 g, 1.1 mmol), in anhydrous ethanol (80 mL, 1.4 mmol), thionyl chloride (96 μL, 1.3 mmol) was added. The resulting solution was stirred for 2 h at room temperature under a nitrogen atmosphere. Next, the excess alcohol was evaporated, and water (10 mL) was added to the residue. The mixture was extracted with AcOEt (3 × 20 mL), and the combined organic phases were dried over Na_2_SO_4_, filtered, and concentrated under reduced pressure, affording **15f** that was used without further purification (0.22 g, 96% yield). The characterization data of compounds **15f** matched those previously reported.^26^^1^H NMR (400 MHz, CDCl_3_) δ 7.92 (d, *J* = 7.6 Hz, 1H), 7.75–7.64 (m, 2H), 7.59 (t, *J* = 7.7 Hz, 1H), 5.87 (s, 1H), 4.28 (m, 2H), 1.30 (m, 3H).

#### Benzyl 3-Oxo-1,3-dihydroisobenzofuran-1-carboxylate (**15g**)

Compound **15g** was prepared following the procedure described for **15f**, employing benzyl alcohol (140 mL, 1.4 mmol) that was used without further purification. Yield: 0.26 g (90% yield). The characterization data of compounds **15g** matched those previously reported.^27^^1^H NMR (400 MHz, CDCl_3_) δ 7.93 (d, *J* = 7.5 Hz, 1H), 7.68 (m, 1H), 7.63–7.58 (m, 2H), 7.44–7.28 (m, 5H), 5.92 (s, 1H), 5.28 (d, *J* = 12.1 Hz, 1H), 5.22 (d, *J* = 12.1 Hz, 1H).

#### General Procedure for the Enantioselective Alkylation of Phthalide 3-Carboxylic *tert*-Butyl Esters **15a**–**15e**

To a solution of phthalide ester **15** (1.0 equiv, 0.1 mmol) in toluene (1 mL), contained in a 4 mL vial, (*R,R*)-**13** (0.05 equiv, 0.005 mmol), alkyl bromide **16** (1.2 equiv, 0.12 mmol), and 50 % aqueous Cs_2_CO_3_ (0.6 mL) were added. The reaction mixture was vigorously stirred (900 rpm) at room temperature for the time specified. After completion (TLC), it was diluted with 1 M HCl (1 mL) and extracted with CH_2_Cl_2_ (3 × 5 mL). The combined organic phases were dried over Na_2_SO_4_ and concentrated under reduced pressure. The crude residue was purified by flash chromatography, affording products **17** as white solids.

#### *tert*-Butyl (*R*)-1-Benzyl-3-oxo-1,3-dihydroisobenzofuran-1-carboxylate (**17aa**)

White solid after flash chromatography (silica gel; petroleum ether/ethyl acetate, 98:2 to 80:20). Yield: 26.5 mg (82%). mp 88–89 °C. [α]_D_^22^ +31.4 (*c* 0.20, CHCl_3_). ^1^H NMR (400 MHz, CDCl_3_) δ 7.75 (d, *J* = 7.5 Hz, 1H), 7.73–7.66 (2H, m), 7.50 (1H, m), 7.20–7.12 (5H, m), 3.64 (1H, d, *J* = 14.3), 3.35 (1H, d, *J* = 14.3), 1.37 (9H, s). ^13^C{^1^H} NMR (100 MHz, CDCl_3_) δ 169.1, 167.2, 147.7, 134.1, 133.4, 130.4, 129.9, 128.1, 127.2, 125.7, 125.6, 122.5, 87.6, 83.8, 43.1, 27.7. The % ee was determined by chiral HPLC (CHIRALPAK AS-H column, *n*-hexane/*i*-PrOH = 90:10, 0.5 mLmin^–1^): τ_minor_ = 16.4 min, τ_major_ = 21.4 min (80% ee). HRMS (MALDI) *m*/*z*: [M + H^+^] calcd for C_20_H_21_O_4_^+^, 325.1434; found, 325.1437.

#### *tert*-Butyl (*R*)-1-(4-(*tert*-Butyl)benzyl)-3-oxo-1,3-dihydroisobenzofuran-1-carboxylate (**17ab**)

White solid after flash chromatography (silica gel; petroleum ether/ethyl acetate, 98:2 to 80:20). Yield: 36.5 mg (96%). mp 99–100 °C. [α]_D20_ +63.5 (*c* 0.20, CHCl_3_). ^1^H NMR (400 MHz, CDCl_3_) δ 7.78 (d, *J* = 7.6 Hz, 1H), 7.75–7.65 (m, 2H), 7.56–7.47 (m, 1H), 7.20 (d, *J* = 8.2 Hz, 2H), 7.11 (d, *J* = 8.2 Hz, 2H), 3.62 (d, *J* = 14.3 Hz, 1H), 3.28 (d, *J* = 14.3 Hz, 1H), 1.35 (s, 9H), 1.24 (s, 9H). ^13^C{^1^H} NMR (100 MHz, CDCl_3_) δ 169.1, 167.1, 150.0, 147.8, 134.1, 130.4, 130.0, 129.8, 125.7, 125.6, 124.9, 122.5, 87.8, 83.6, 42.8, 34.3, 31.2, 27.6. The % ee was determined by chiral HPLC (CHIRALPAK AS-H column, *n*-hexane/*i*-PrOH = 90:10, 0.3 mLmin^–1^): τ_minor_ = 28.1 min, τ_major_ = 31.4 min (88% ee). HRMS (MALDI) *m*/*z*: [M + Na^+^] calcd for C_24_H_28_NaO_4_^+^, 403.1880; found, 403.1882.

#### *tert*-Butyl (*R*)-1-(4-Methylbenzyl)-3-oxo-1,3-dihydroisobenzofuran-1-carboxylate (**17ac**)

White solid after flash chromatography (silica gel; petroleum ether/ethyl acetate, 98:2 to 80:20). Yield: 32.1 mg, (95%). mp 93–94 °C. [α]_D20_ +24.4 (*c* 0.20, CHCl_3_). ^1^H NMR (400 MHz, CDCl_3_) δ 7.75 (d, *J* = 7.6 Hz, 1H), 7.72–7.64 (m, 2H), 7.56–7.45 (m, 1H), 7.01 (d, *J* = 7.9 Hz, 2H), 6.96 (d, *J* = 7.9 Hz, 2H), 3.60 (d, *J* = 14.3 Hz, 1H), 3.32 (d, *J* = 14.3 Hz, 1H), 2.23 (s, 3H), 1.39 (s, 9H). ^13^C{^1^H} NMR (100 MHz, CDCl_3_) δ 169.1, 167.2, 147.6, 136.7, 134.0, 130.2, 130.1, 129.8, 128.7, 125.7, 125.6, 122.5, 87.7, 83.7, 42.6, 27.7, 21.0. The % ee was determined by chiral HPLC (CHIRALPAK AS-H column, *n*-hexane/*i*-PrOH = 90:10, 0.5 mLmin^–1^): τ_minor_ = 22.3 min, τ_major_ = 32.0 min (82% ee). HRMS (MALDI) *m*/*z*: [M + K^+^] calcd for C_21_H_22_KO_4_^+^, 377.1150; found, 377.1163.

#### *tert*-Butyl (*R*)-1-(2-Methylbenzyl)-3-oxo-1,3-dihydroisobenzofuran-1-carboxylate (**17ad**)

White solid after flash chromatography (silica gel; petroleum ether/ethyl acetate, 98:2 to 80:20). Yield: 32.5 mg (96%). mp 90–91 °C. [α]_D20_ +40.8 (*c* 0.40, CHCl_3_). ^1^H NMR (400 MHz, CDCl_3_) δ 7.81–7.66 (m, 3H), 7.57–7.49 (m, 1H), 7.11–7.01 (m, 3H), 6.95 (m, 1H), 3.75 (d, *J* = 14.7 Hz, 1H), 3.35 (d, *J* = 14.7 Hz, 1H), 2.33 (s, 3H), 1.38 (s, 9H). ^13^C{^1^H} NMR (100 MHz, CDCl_3_) δ 168.9, 167.4, 147.9, 137.6, 134.0, 131.9, 130.4 (2C), 129.9, 127.2, 125.6 (2C), 125.4, 122.6, 88.1, 83.6, 39.4, 27.7, 20.1. The % ee was determined by chiral HPLC (CHIRALPAK AS-H column, *n*-hexane/*i*-PrOH = 90:10, 0.5 mLmin^–1^): τ_minor_ = 15.0 min, τ_major_ = 17.8 min (84% ee). HRMS (MALDI) *m*/*z*: [M + K^+^] calcd for C_21_H_22_NaO_4_^+^, 361.1410; found, 361.1421.

#### *tert*-Butyl (*R*)-1-(4-Methoxybenzyl)-3-oxo-1,3-dihydroisobenzofuran-1-carboxylate (**17ae**)

White solid after flash chromatography (silica gel, petroleum ether/ethyl acetate 98:2 to 80:20). Yield: 32.6 mg (92%). mp 97–98 °C. [α]_D20_ +11.9 (*c* 0.80, CHCl_3_). ^1^H NMR (600 MHz, CDCl_3_) δ 7.75 (d, *J* = 7.7 Hz, 1H), 7.71–7.67 (m, 2H), 7.50 (m, 1H), 7.05 (d, *J* = 8.2 Hz), 6.69 (d, *J* = 8.2 Hz), 3.73 (s, 3H), 3.58 (d, *J* = 14.3 Hz), 3.30 (d, *J* = 14.3 Hz), 1.39 (s, 9H). ^13^C{^1^H} NMR (150 MHz, CDCl_3_) δ 169.1, 167.3, 158.7, 147.7, 134.1, 131.5, 129.8, 125.9, 125.6, 125.3, 122.5, 113.5, 87.8, 83.7, 55.1, 42.3, 27.8. The % ee was determined by chiral HPLC (CHIRALPAK AS-H column, *n*-hexane/*i*-PrOH = 90:10, 0.5 mLmin^–1^): τ_minor_ = 26.8 min, τ_major_ = 42.9 min (80% ee). HRMS (ESI) *m*/*z*: [M + Na^+^] calcd for C_21_H_22_NaO_5_^+^, 377.1359; found, 377.1359.

#### *tert*-Butyl (*R*)-1-(4-Chlorobenzyl)-3-oxo-1,3-dihydroisobenzofuran-1-carboxylate (**17af**)

White solid after flash chromatography (silica gel; petroleum ether/ethyl acetate, 98:2 to 80:20). Yield: 33.7 mg (94%). mp 110–111 °C. [α]_D20_ +14.4 (*c* 0.20, CHCl_3_). ^1^H NMR (400 MHz, CDCl_3_) δ 7.82–7.74 (m, 1H), 7.74–7.66 (m, 2H), 7.57–7.48 (m, 1H), 7.15 (d, *J* = 8.3 Hz, 2H), 7.09 (d, *J* = 8.3 Hz, 2H), 3.62 (d, *J* = 14.3 Hz, 1H), 3.31 (d, *J* = 14.3 Hz, 1H), 1.38 (s, 9H). ^13^C{^1^H} NMR (100 MHz, CDCl_3_) δ 168.8, 167.0, 147.3, 134.2, 133.2, 131.8, 131.7, 130.0, 128.2, 125.8, 125.6, 122.3, 87.3, 83.9, 42.2, 27.7. The % ee was determined by chiral HPLC (CHIRALPAK AS-H column, *n*-hexane/*i*-PrOH = 90:10, 0.5 mLmin^–1^): τ_minor_ = 21.3 min, τ_major_ = 28.8 min (80% ee). HRMS (MALDI) *m*/*z*: [M + K^+^] calcd for C_20_H_19_ClKO_4_^+^, 397.0603; found, 397.0609.

#### *tert*-Butyl (*R*)-1-(4-Nitrobenzyl)-3-oxo-1,3-dihydroisobenzofuran-1-carboxylate (**17ag**)

White solid after flash chromatography (silica gel; petroleum ether/ethyl acetate, 98:2 to 80:20). Yield: 33.6 mg (91%). mp 96–97 °C. [α]_D20_ +37.4 (*c* 0.80, CHCl_3_). ^1^H NMR (400 MHz, CDCl_3_) δ 8.05 (d, *J* = 8.6 Hz, 2H), 7.82–7.69 (m, 3H), 7.60–7.49 (m, 1H), 7.56 (d, *J* = 8.6 Hz, 2H), 3.79 (d, *J* = 14.2 Hz, 1H), 3.43 (d, *J* = 14.2 Hz, 1H), 1.37 (s, 9H). ^13^C{^1^H} NMR (100 MHz, CDCl_3_) δ 168.6, 166.6, 147.1, 147.0, 141.1, 134.5, 131.3, 130.32, 126.0, 125.4, 123.2, 122.1, 86.7, 84.3, 42.4, 27.6. The % ee was determined by chiral HPLC (CHIRALPAK AS-H column, *n*-hexane/*i*-PrOH = 80:20, 0.5 mLmin^–1^): τ_minor_ = 35.2 min, τ_major_ = 45.7 min (82% ee). HRMS (MALDI) *m*/*z*: [M + K^+^] calcd for C_20_H_19_NKO_6_^+^, 408.0844; found, 408.0844.

#### *tert*-Butyl (*R*)-1-(Naphthalen-2-ylmethyl)-3-oxo-1,3-dihydroisobenzofuran-1-carboxylate (**17ah**)

White solid after flash chromatography (silica gel; petroleum ether/ethyl acetate, 98:2 to 80:20). Yield: 24.3 mg (65%). mp 128–129 °C. [α]_D20_ +37.1 (*c* 0.20, CHCl_3_). ^1^H NMR (400 MHz, CDCl_3_) δ 7.80–7.68 (m, 5H), 7.66 (d, *J* = 8.4 Hz, 1H), 7.63 (s, 1H), 7.53–7.46 (m, 1H), 7.44–7.38 (m, 2H), 7.30 (d, *J* = 8.4 Hz, 1H), 3.82 (d, *J* = 14.3 Hz, 1H), 3.51 (d, *J* = 14.3 Hz, 1H), 1.36 (s, 9H). ^13^C{^1^H} NMR (100 MHz, CDCl_3_) δ 169.0, 167.1, 147.6, 134.1, 133.0, 132.4, 131.0, 129.9, 129.3, 128.3, 127.6 (2C), 127.4, 125.9, 125.7, 125.7 (2C), 122.5, 87.7, 83.8, 43.2, 27.7. The % ee was determined by chiral HPLC (CHIRALPAK AS-H column, *n*-hexane/*i*-PrOH = 90:10, 0.5 mLmin^–1^): τ_minor_ = 26.2 min, τ_major_ = 36.7 min (74% ee). HRMS (MALDI) *m*/*z*: [M + K^+^] calcd for C_24_H_22_KO_4_^+^, 413.1150; found, 413.1157.

#### *tert*-Butyl (*R*)-1-Allyl-3-oxo-1,3-dihydroisobenzofuran-1-carboxylate (**17ai**)

White solid after flash chromatography (silica gel; petroleum ether/ethyl acetate, 98:2 to 80:20). Yield: 14.8 mg (54%). mp 90–91 °C. [α]_D20_ +44.3 (*c* 1.0, CHCl_3_). ^1^H NMR (400 MHz, CDCl_3_) δ 7.88 (d, *J* = 7.6 Hz, 1H), 7.73–7.66 (m, 1H), 7.64–7.52 (m, 2H), 5.62 (m, 1H), 5.20–5.05 (m, 2H), 3.10 (dd, *J* = 14.4, 7.7 Hz, 1H), 2.77 (dd, *J* = 14.4, 6.5 Hz, 1H), 1.43 (s, 9H). ^13^C{^1^H} NMR (100 MHz, CDCl_3_) δ 169.2, 167.0, 147.7, 134.3, 129.9, 129.8, 125.7, 125.6, 122.3, 120.7, 87.1, 83.7, 41.3, 27.7. The % ee was determined by chiral HPLC (CHIRALPAK AS-H column, *n*-hexane/*i*-PrOH = 90:10, 0.5 mLmin^–1^): τ_minor_ = 18.2 min, τ_major_ = 23.5 min (84% ee). HRMS (MALDI) *m*/*z*: [M + K^+^] calcd for C_16_H_18_KO_4_^+^, 313.0837; found, 313.0842.

#### *tert*-Butyl (*R*)-1-Benzyl-5-methoxy-3-oxo-1,3-dihydroisobenzofuran-1-carboxylate (**17ba**)

White solid after flash chromatography (silica gel; petroleum ether/ethyl acetate, 98:2 to 80:20). Yield: 34.0 mg (96%). mp 109–110 °C. [α]_D20_ +70.4 (*c* 0.80, CHCl_3_). ^1^H NMR (400 MHz, CDCl_3_) δ 7.57 (d, *J* = 8.4 Hz, 1H), 7.23 (dd, *J* = 8.4, 2.3 Hz, 1H), 7.20–7.10 (m, 6H), 3.83 (s, 3H), 3.60 (d, *J* = 14.3 Hz, 1H), 3.33 (d, *J* = 14.3 Hz, 1H), 1.37 (s, 9H). ^13^C{^1^H} NMR (100 MHz, CDCl_3_) δ 169.0, 167.3, 161.1, 140.0, 133.4, 130.4, 128.01, 127.2, 127.1, 123.4, 122.9, 107.3, 87.4, 83.6, 55.7, 43.0, 27.7. The % ee was determined by chiral HPLC (CHIRALPAK AS-H column, *n*-hexane/*i*-PrOH = 98:2, 1.0 mLmin^–1^): τ_minor_ = 29.1 min, τ_major_ = 32.3 min (80% ee). HRMS (MALDI) *m*/*z*: [M + K^+^] calcd for C_21_H_22_KO_5_^+^, 393.1099; found, 393.1110.

#### *tert*-Butyl (*R*)-1-Benzyl-5-chloro-3-oxo-1,3-dihydroisobenzofuran-1-carboxylate (**17ca**)

White solid after flash chromatography (silica gel; petroleum ether/ethyl acetate, 98:2 to 80:20). Yield: 34.4 mg (96%). mp 98–99 °C. [α]_D20_ +16.7 (*c* 0.68, CHCl_3_). ^1^H NMR (600 MHz, CDCl_3_) δ 7.70 (m, 1H), 7.65–7.63 (m, 2H), 7.20–7.16 (m, 3H), 7.14–7.11 (m, 2H), 3.62 (d, *J* = 14.4 Hz, 1H), 3.36 (d, *J* = 14.4 Hz, 1H), 1.39 (s, 9H). ^13^C{^1^H} NMR (150 MHz, CDCl_3_) δ 167.5, 166.8, 145.8, 136.3, 134.4, 133.0, 130.4, 128.2, 127.6, 127.4, 125.5, 123.9, 87.6, 84.1, 43.0, 27.7. The % ee was determined by chiral HPLC (CHIRALPAK AS-H column, *n*-hexane/*i*-PrOH = 90:10, 0.5 mLmin^–1^): τ_major_ = 12.1 min, τ_minor_ = 14.1 min (82% ee). HRMS (MALDI) *m*/*z*: [M + K^+^] calcd for C_20_H_19_ClKO_4_^+^, 397.0603; found, 397.0604.

#### *tert*-Butyl (*R*)-1-Benzyl-6-methoxy-3-oxo-1,3-dihydroisobenzofuran-1-carboxylate (**17da**)

White solid after flash chromatography (silica gel; petroleum ether/ethyl acetate, 98:2 to 80:20). Yield: 34.0 mg (96%). mp 88–89 °C. [α]_D20_ +5.6 (*c* 0.80, CHCl_3_). ^1^H NMR (600 MHz, CDCl_3_) δ 7.66 (d, *J* = 8.7 Hz, 1H), 7.21–7.16 (m, 5H), 7.13 (d, *J* = 2.1 Hz, 1H), 7.01 (dd, *J* = 8.7, 2.1 Hz, 1H), 3.93 (s, 3H), 3.61 (d, *J* = 13.8 Hz, 1H), 3.32 (d, *J* = 13.8 Hz, 1H), 1.37 (s, 9H).^13^C{^1^H} NMR (150 MHz, CDCl_3_) δ 168.8, 167.4, 164.7, 150.5, 133.6, 130.5, 128.1, 127.2, 127.1, 118.2, 117.1, 106.8, 87.0, 83.7, 55.9, 43.4, 27.8. The % ee was determined by chiral HPLC (CHIRALPAK AS-H column, *n*-hexane/*i*-PrOH = 90:10, 1.0 mLmin^–1^): τ_minor_ = 29.9 min, τ_major_ = 61.4 min (78% ee). HRMS (MALDI) *m*/*z*: [M + H^+^] calcd for C_21_H_23_O_5_^+^, 355.1540; found, 355.1543.

#### *tert*-Butyl (*R*)-1-Benzyl-6-chloro-3-oxo-1,3-dihydroisobenzofuran-1-carboxylate (**17ea**)

White solid after flash chromatography (silica gel; petroleum ether/ethyl acetate, 98:2 to 80:20). Yield: 34.1 mg (95%). mp 95–96 °C. [α]_D20_ +32.2 (*c* 0.85, CHCl_3_). ^1^H NMR (600 MHz, CDCl_3_) δ 7.70 (d, *J* = 1.6 Hz, 1H), 7.68 (d, *J* = 8.0 Hz, 1H), 7.48 (dd, *J* = 8.0, 1.6 Hz, 1H), 7.20–7.17 (m, 3H), 7.16–7.13 (m, 2H), 3.62 (d, *J* = 13.6 Hz, 1H), 3.33 (d, *J* = 13.6 Hz, 1H), 1.39 (s, 9H). ^13^C{^1^H} NMR (150 MHz, CDCl_3_) δ 167.8, 166.7, 149.3, 140.8, 132.9, 130.6, 130.4, 128.2, 127.4, 126.7, 124.3, 123.1, 87.2, 84.3, 43.2, 27.7. The % ee was determined by chiral HPLC (CHIRALPAK AS-H column, *n*-hexane/*i*-PrOH = 90:10, 0.5 mLmin^–1^): τ_minor_ = 13.2 min, τ_major_ = 18.4 min (80% ee). HRMS (MALDI) *m*/*z*: [M + K^+^] calcd for C_20_H_19_ClKO_4_^+^, 397.0603; found, 397.0603.

### Scaled-up Procedure for the Enantioselective Alkylation of Phthalide Ester **15a**

To a solution of phthalide ester **15a** (234 mg, 1.00 mmol) in toluene (10 mL), contained in a 50 mL round-bottom flask, (*R,R*)-**13** (22 mg, 0.020 mmol), benzyl bromide (205 mg, 1.20 mmol), and 50% aqueous Cs_2_CO_3_ (6.6 mL) were added. The reaction mixture was vigorously stirred (900 rpm) at room temperature for 20 h followed by addition of 1 M HCl (20 mL) and extraction with CH_2_Cl_2_ (3 × 50 mL). The combined organic phases were dried over Na_2_SO_4_ and concentrated under reduced pressure. The crude residue was purified by flash chromatography (silica gel; petroleum ether/ethyl acetate, 98:2 to 80:20), affording **17aa** as a white solid (201 mg, 62% yield). The % ee was determined by chiral HPLC, as described above (78% ee).

### Recrystallization of Compound **17aa**

Compound **17aa** (26.5 mg, 82% yield, 80% ee) was dissolved in hot hexane (1 mL), and the resulting solution was cooled down at 4 °C. After 20 h, crystals of racemate were formed. The supernatant solution was separated and concentrated under reduced pressure, affording enantioenriched **17aa** (21.9 mg, 66% yield). [α]_D20_ = +37.2 (*c* 0.20, CHCl_3_). The % ee was determined by chiral HPLC, as described above (94% ee).

### Recrystallization of Compound **17ai**

Compound **17ai** (14.8 mg, 54% yield, 84% ee) was dissolved in hot hexane (1 mL), and the resulting solution was cooled down at 4 °C. After 20 h, crystals of racemate were formed. The supernatant solution was separated and concentrated under reduced pressure, affording enantioenriched **17ai** (14.0 mg, 51% yield). [α]_D20_ +55.6 (*c* 1.0, CHCl_3_). The % ee was determined by chiral HPLC, as described above (95% ee).

### Formal Synthesis of (+)-(9*S*,13*R*)-13-Hydroxyisocyclocelabenzine

#### (*R*)-1-Allyl-3-oxo-1,3-dihydroisobenzofuran-1-carboxamide (**19**)

To a solution of **17ai** (26.1 mg, 0.12 mmol, 95% ee after recrystallization, see above) in anhydrous CH_2_Cl_2_ (0.65 mL), trifluoroacetic acid (0.12 mL) was added dropwise. After stirring for 8 h, the mixture was concentrated under reduced pressure, affording carboxylic acid **25** as a white solid (28.0 mg), which was used in the next step without further purification.

Carboxylic acid **25** was dissolved in dioxane (0.1 mL), and aqueous ammonia (0.2 mL) was added dropwise at 0 °C. Once the addition was complete, the mixture was allowed to warm to room temperature, and H_2_O (0.2 mL) was added. The resulting reaction mixture was stirred for 2 h and then extracted with CH_2_Cl_2_ (3 × 5 mL). The combined organic phases were dried over Na_2_SO_4_ and concentrated under reduced pressure. The crude residue was passed through a silica gel short path by eluting with ethyl acetate, affording **19** as a colorless oil (21.0 mg, 74% yield). [α]_D20_ +93.2 (*c* 1.0, CHCl_3_). ^1^H NMR (400 MHz, CDCl_3_) δ 7.86–7.82 (m, 2H), 7.71 (t, *J* = 7.4 Hz, 1H), 7.56 (t, *J* = 7.4 Hz, 1H), 6.57 (bs, 1H), 5.84 (bs, 1H), 5.54 (m, 1H), 5.18–5.01 (m, 2H), 3.11 (dd, *J* = 14.2, 7.4 Hz, 1H), 2.80 (dd, *J* = 14.2, 6.8 Hz, 1H). ^13^C{^1^H} NMR (100 MHz, CDCl_3_) δ 170.9, 170.0, 148.0, 135.0, 130.0, 129.4, 125.5, 124.4, 123.5, 121.2, 87.9, 42.0. The % ee was determined by chiral HPLC (CHIRALPAK AS-H column, *n*-hexane/*i*-PrOH = 90:10, 0.5 mLmin^–1^): τ_minor_ = 18.6 min, τ_major_ = 46.6 min (95% ee). HRMS (ESI) *m*/*z*: [M + H^+^] calcd for C_12_H_12_O_3_N^+^, 218.0812; found, 218.0814.

#### (*R*)-1-Allyl-3-oxo-1,3-dihydroisobenzofuran-1-arbonitrile (**18**)

To a solution of **19** (19.3 mg, 0.097 mmol) in anhydrous CH_3_CN (1.0 mL), Ph_3_PO (2.7 mg, 9.7 μmol) and NEt_3_ (40 μL, 29 mg, 0.29 mmol) were added, and then (COCl)_2_ (17 μL, 25 mg, 0.19 mmol) was injected dropwise. After stirring for 2 h, the reaction mixture was diluted with CH_2_Cl_2_, filtered on a small pad of celite, and then concentrated under reduced pressure. The crude residue was purified by flash chromatography (silica gel; petroleum ether/ethyl acetate, 90:10 to 60:40), affording **18** as a white solid (14.5 mg, 75% yield). mp 77–78 °C. [α]_D20_ −11.1 (*c* 1.0, CHCl_3_). ^1^H NMR (400 MHz, CDCl_3_) δ 7.96 (d, *J* = 7.9 Hz, 1H), 7.82 (m, 1H), 7.71–7.64 (m, 2H), 5.72 (m, 1H), 5.33–5.27 (m, 2H), 3.03–2.90 (m, 2H). ^13^C NMR (100 MHz, CDCl_3_) δ 167.1, 145.4, 135.4, 131.3, 127.3, 126.5, 124.7, 123.4, 122.3, 115.8, 77.0, 43.4. The % ee was determined by chiral HPLC (CHIRALPAK AS-H column, *n*-hexane/*i*-PrOH = 90:10, 0.5 mLmin^–1^): τ_minor_ = 29.2 min, τ_major_ = 32.2 min (95% ee). HRMS (ESI) *m*/*z*: [M + H^+^] calcd for C_12_H_10_O_2_N^+^, 200.0706; found, 200.0706.

## Supporting Information Available

Table of optimization, synthesis of ethyl and benzyl substrates, copies of NMR spectra, HPLC traces, and crystallographic data for **17aa** (PDF)
Crystallographic data for **17aa** (CIF)

## Supplementary Material

jo0c00880_si_001.pdf

jo0c00880_si_002.cif
