# Supplementary material for: An Entry to Enantioenriched 3,3-Disubstituted Phthalides through Asymmetric Phase-Transfer-Catalyzed γ-Alkylation
Source: J Org Chem. 2020 May 14;85(11):7476–84. doi: 10.1021/acs.joc.0c00880 (PMC8007094; doi:10.1021/acs.joc.0c00880)
Supplement: Supplementary file 1 — jo0c00880_si_001.pdf [file jo0c00880_si_001.pdf]

Supporting Information

An Entry to Enantioenriched 3,3-Disubstituted Phthalides through Asymmetric Phase-Transfer Catalyzed γ-Alkylation

Marina Sicignano,† Rosaria Schettini,† Giovanni Pierri,† Maria Leda Marino,† Irene Izzo,† Francesco De Riccardis,† Luca Bernardi, § and Giorgio Della Sala†*

† Dipartimento di Chimica e Biologia "A. Zambelli", Università degli Studi di Salerno, Via Giovanni Paolo II 132, 84084 Fisciano, SA, Italy.

§ Department of Industrial Chemistry "Toso Montanari" & INSTM RU Bologna, Alma Mater Studiorum University of Bologna, Viale del Risorgimento 4, 40136 Bologna, Italy.

CONTENTS

Optimization of reaction conditions S2

X-ray crystallography S3

Copies of ^{1}H and ^{13}C NMR spectra S5

Copies of HPLC traces S26

Copies of HPLC traces after recrystallization S40

Optimization of reaction conditions

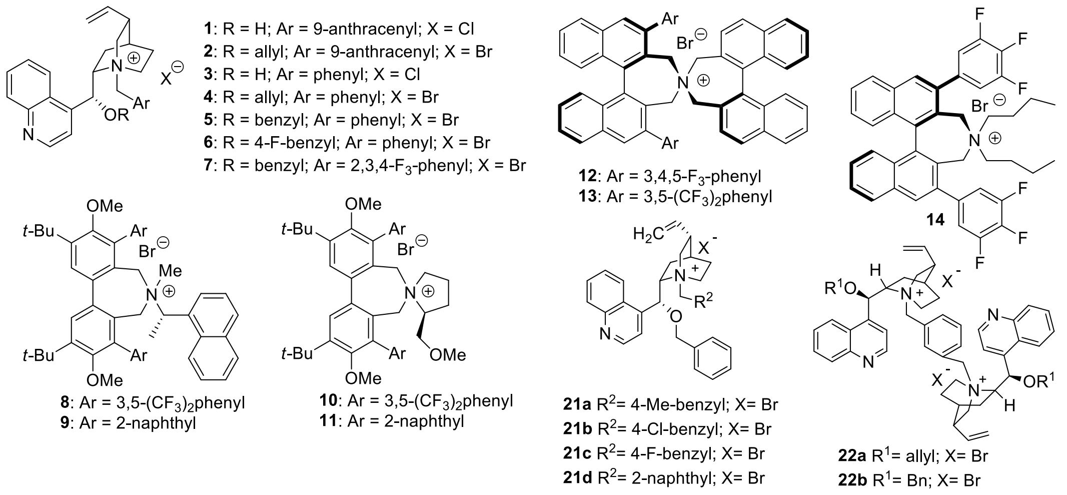

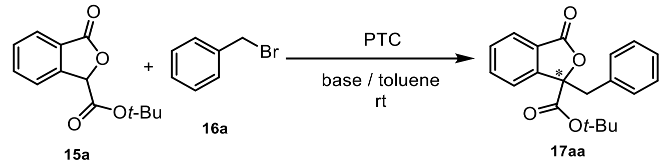

entry	catalyst	base	t (h)	yield (%)[a]	ee (%)
12	**1**	CsOH aq. 50%	5	63	22 (*S*)
13	**2**	CsOH aq. 50%	3	87	22 (*S*)
14	**3**	CsOH aq. 50%	5	25	20 (*R*)
15	**4**	CsOH aq. 50%	18	88	4 (*R*)
16	**5**	CsOH aq. 50%	4	45	30 (*R*)
17	**6**	CsOH aq. 50%	2	40	14 (*R*)
18	**22a**	CsOH aq. 50%	24	88	24 (*S*)
19	**22b**	CsOH aq. 50%	4	37	16 (*S*)
20	**21a**	CsOH aq. 50%	0.5	60	18 (*R*)
21	**21b**	CsOH aq. 50%	0.5	64	20 (*R*)
22	**21c**	CsOH aq. 50%	0.5	58	20 (*R*)
23	**21d**	CsOH aq. 50%	0.5	80	28 (*R*)
24	**8**	CsOH aq. 50%	168	75	28 (*S*)
25	**9**	CsOH aq. 50%	120	26	2 (*S*)
26	**10**	CsOH aq. 50%	36	64	18 (*S*)
27	**11**	CsOH aq. 50%	32	67	20 (*S*)
28	**13**	CsOH (s)	168	82	26 (*R*)
29	**13**	KOH (s)	24	-	-
30	**13**	Cs_2CO_3 (s)	24	-	-

Table S1. Table of optimization for PTC alkylation of **15a** with benzyl bromide

X-ray crystallography

The compound **17aa** (9 mg) was dissolved in hot hexane (1.0 mL) and the resulting solution was cooled down at 4 °C. After 19 hours, chiral crystals suitable for X-ray diffraction analysis were obtained. A colorless prismatic single crystal of 0.56 mm × 0.38 mm × 0.27 mm was selected and mounted on a cryoloop with paratone oil and measured at room temperature with a Bruker D8 QUEST diffractometer equipped with a PHOTON II detector using Cu*Kα* radiation (λ= 1.54178 Å). Data Indexing was performed using APEX3.[3] Data integration and reduction were performed using SAINT.[3] Absorption correction was performed by multi-scan method in SADABS.[3] The structures were solved using SHELXS-97[4] and refined by means of full matrix least-squares based on F^2 using the program SHELXL.[5] Non-hydrogen atoms were refined anisotropically, hydrogen atoms were positioned geometrically and included in structure factors calculations but not refined. ORTEP diagrams were drawn using OLEX2.[6] The chirality on carbon atom C7 (R) was successfully assigned by anomalous-dispersion effects in diffraction measurements on the crystal (Flack parameter 0.07(5)).

	**17aa**
**T (K)**	296
**Formula**	$C_{20}H_{20}O_4$
**Formula weight**	324.36
**System**	Orthorhombic
**Space group**	$P2_12_12_1$
a **(Å)**	6.0173(2)
b **(Å)**	16.8850(5)
c **(Å)**	17.2658(5)
α **(°)**	90
β **(°)**	90
γ **(°)**	90
**V (Å^3)**	1754.24(9)
**Z**	4
**Dx (g cm^{-3})**	1.228
**λ (Å)**	1.54178
**μ (mm^{-1})**	0.690
**F_{000}**	688
**R1 (I > 2σI)**	0.0343(3157)
**$_wR_2$**	0.0898(3280)
**N. of param.**	220
**GooF**	1.058
**ρ_{min}, ρ_{max} (eÅ^{-3})**	-0.13, 0.12

Table S2. Crystallographic data for compound

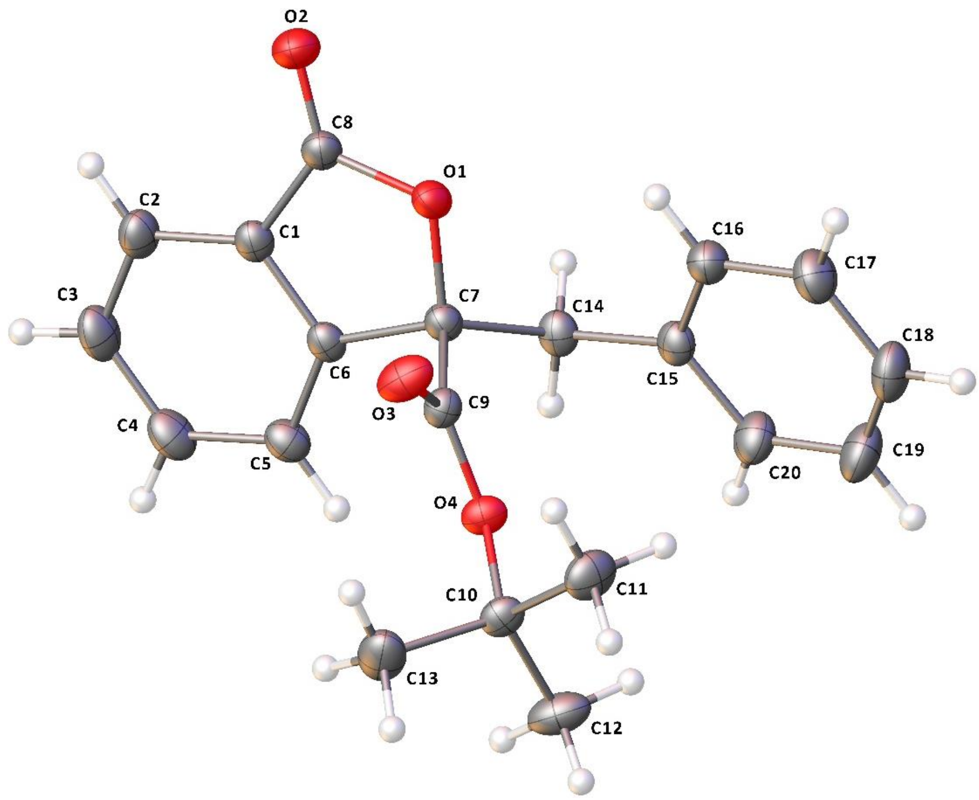

Figure S1 ORTEP diagrams for compound **17aa**. Atom types: C grey, O red, H white. Ellipsoids are drawn at 20% probability level.

References

1. Tatsugi, J.; Hara, T.; Izawa, Y. *Chem. Lett.* **1997**, 177.

2. Ishibashi, M.; Wagner, A.; Mioskowski, C.; Sylvain, C.; *PCT Int. Appl.*, **2001**, WO 2001072730, A1 20011004.

3. Bruker (**2015**). APEX3, SAINT and SADABS. Bruker AXS Inc, Madison, Wisconsin, USA.

4. Sheldrick, G. M. *Acta Cryst.* **2008**, *A64*, 112.

5. Sheldrick, G. M. *Acta Cryst*. **2015**, C71, 3.

6. Dolomanov, O. V.; Bourhis, L. J.; Gildea, R. J.; Howard, J. A. K; Puschmann, H. *J. Appl. Cryst*. **2009**, 339.

Copies of ^{1}H and ^{13}C NMR spectra

Compound 15a

^{1}H NMR (400 MHz, $CDCl_3$)

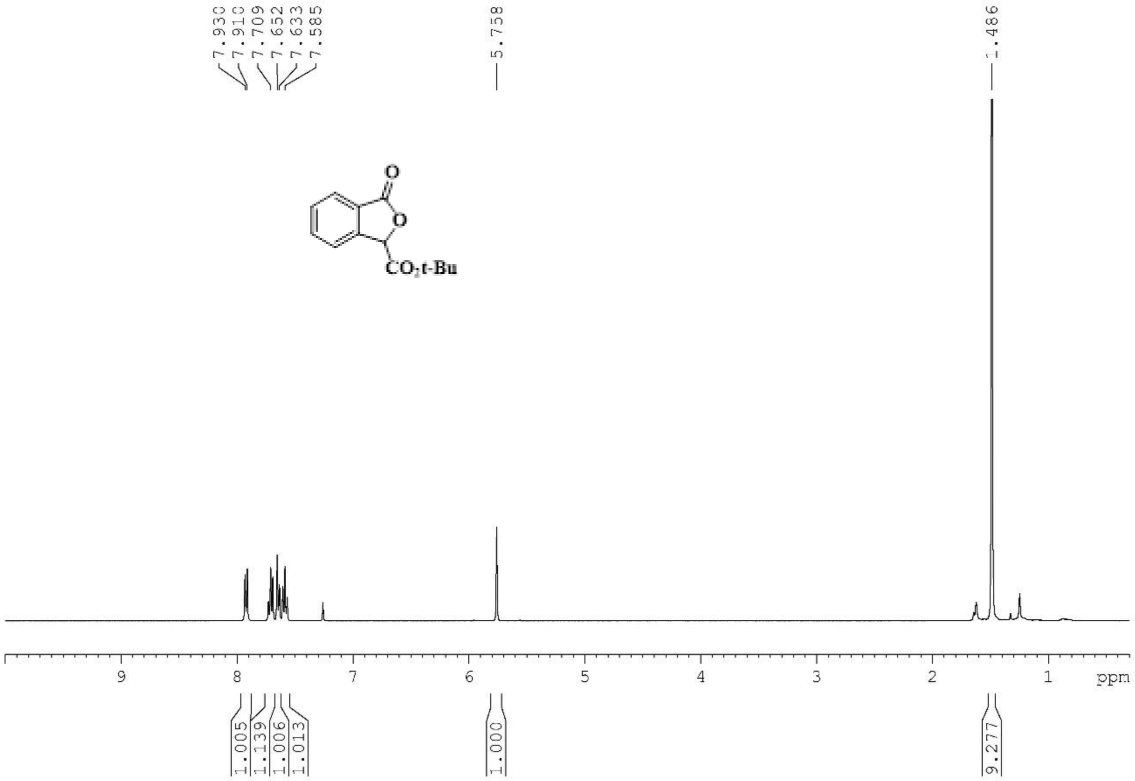

Compound 15b

^{1}H NMR (400 MHz, $CDCl_3$)

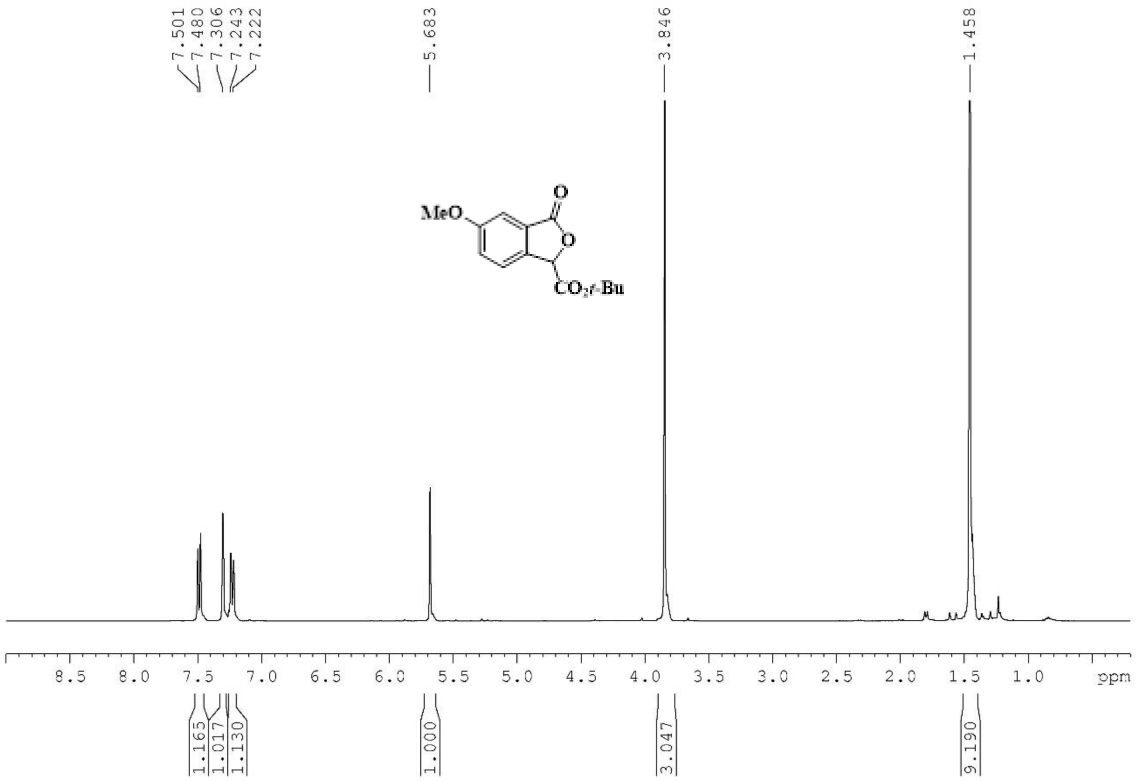

$^{13}C\{^1H\}$ NMR (100 MHz, $CDCl_3$)

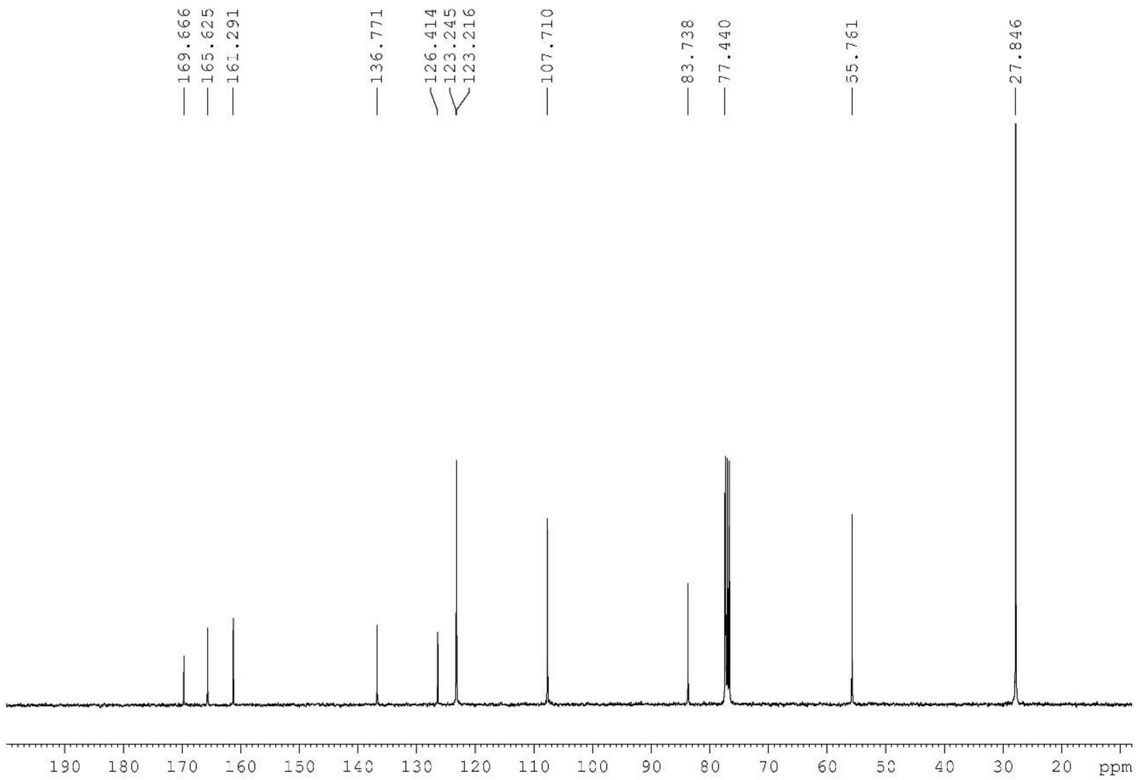

Compound 15c

^{1}H NMR (400 MHz, $CDCl_3$)

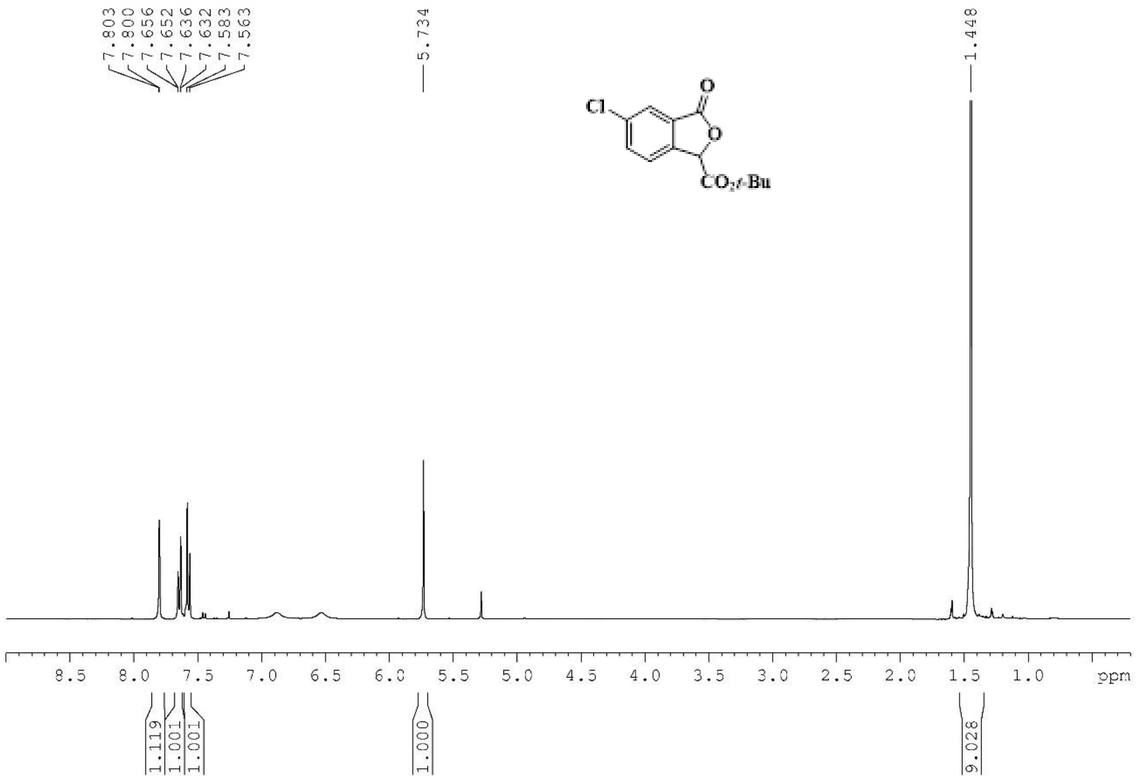

$^{13}C\{^{1}H\}$ NMR (100 MHz, $CDCl_3$)

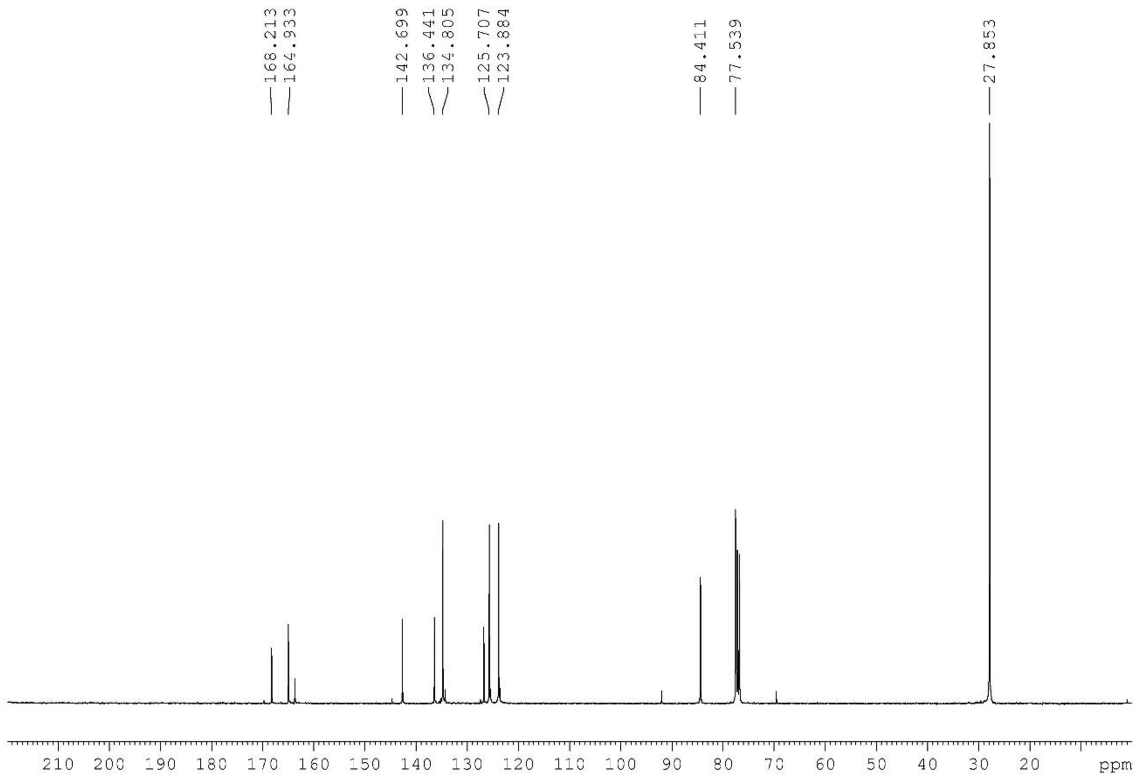

Compound 15d

^{1}H NMR (600 MHz, $CDCl_3$)

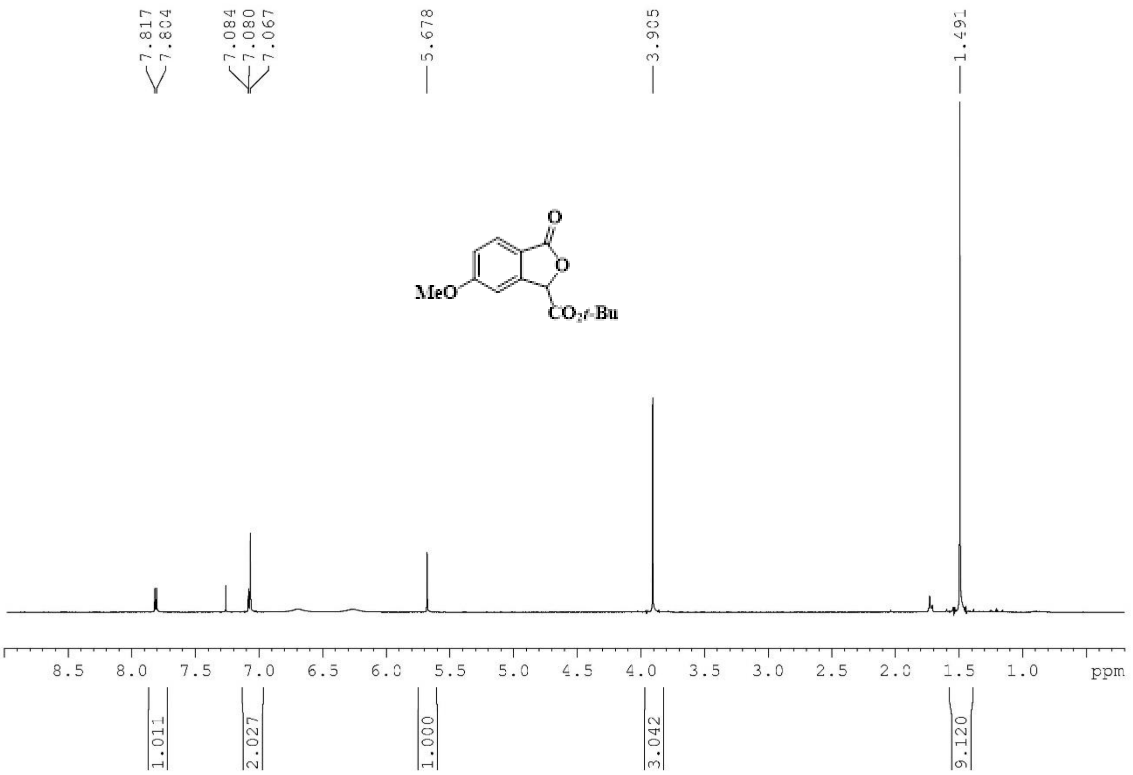

$^{13}C\{^{1}H\}$ NMR (150 MHz, $CDCl_3$)

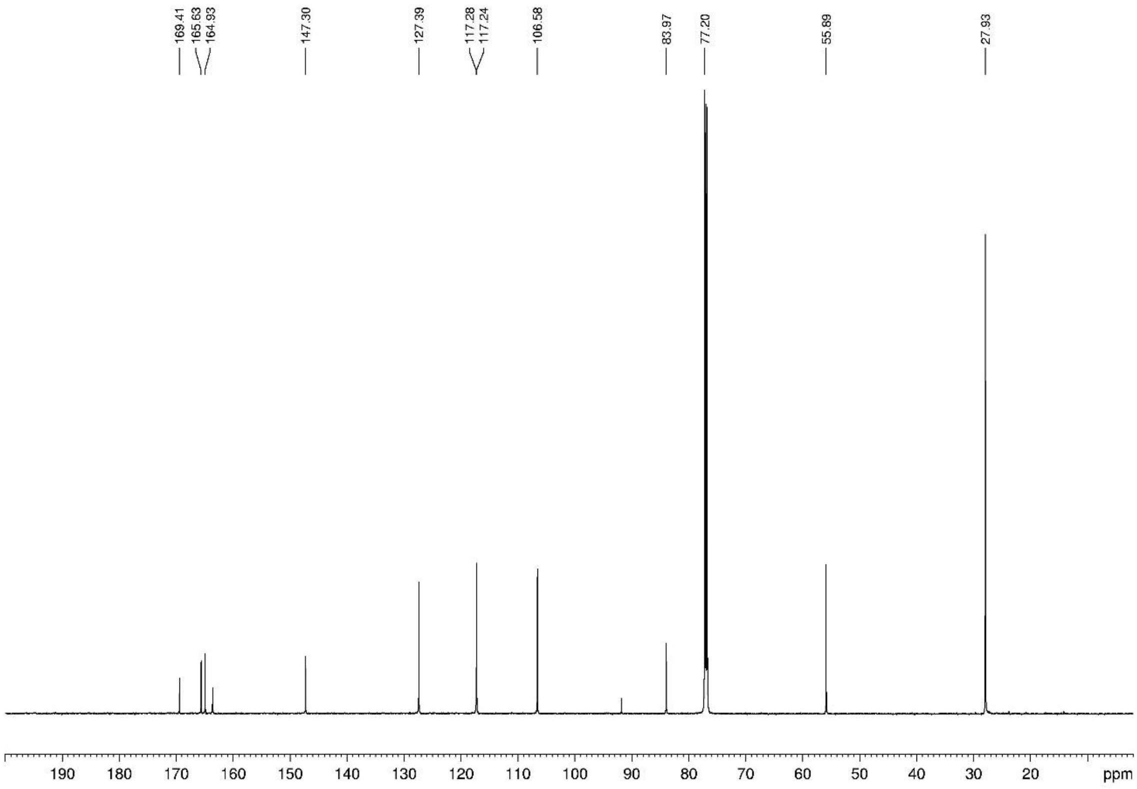

Compound 15e

^{1}H NMR (400 MHz, $CDCl_3$)

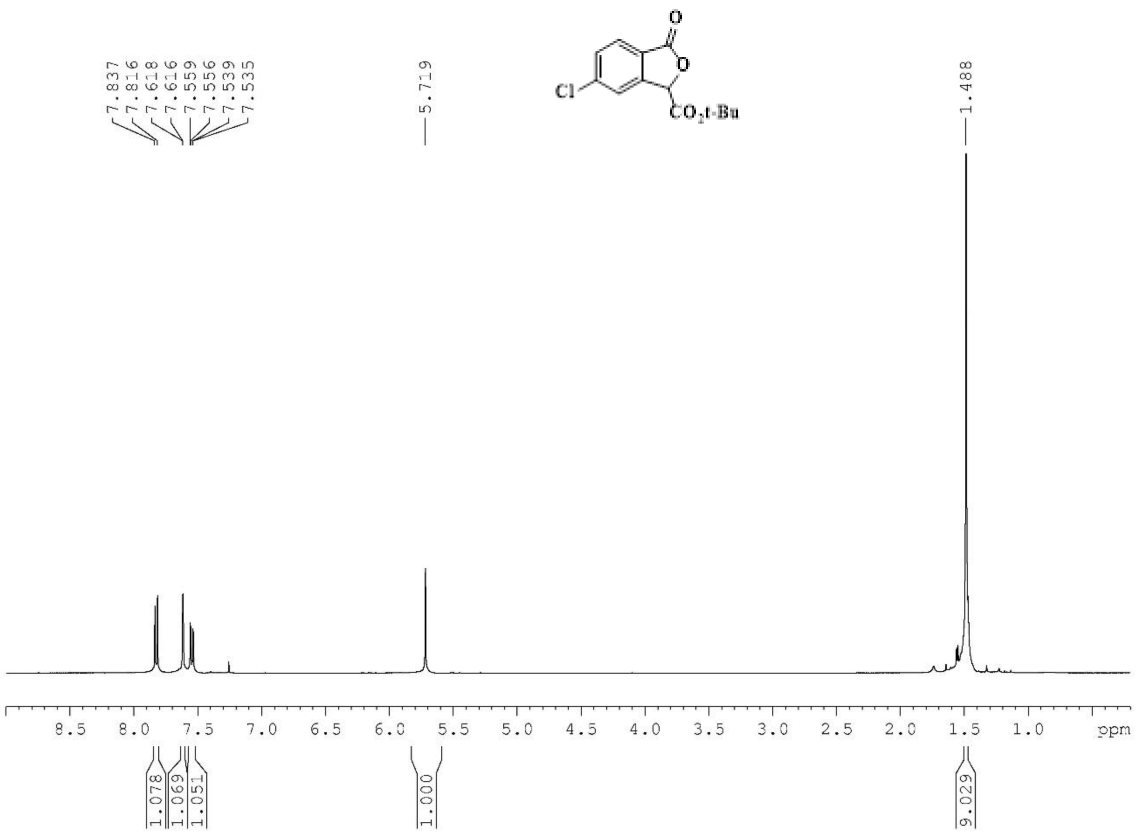

^{13}C{^{1}H} NMR (100 MHz, $CDCl_3$)

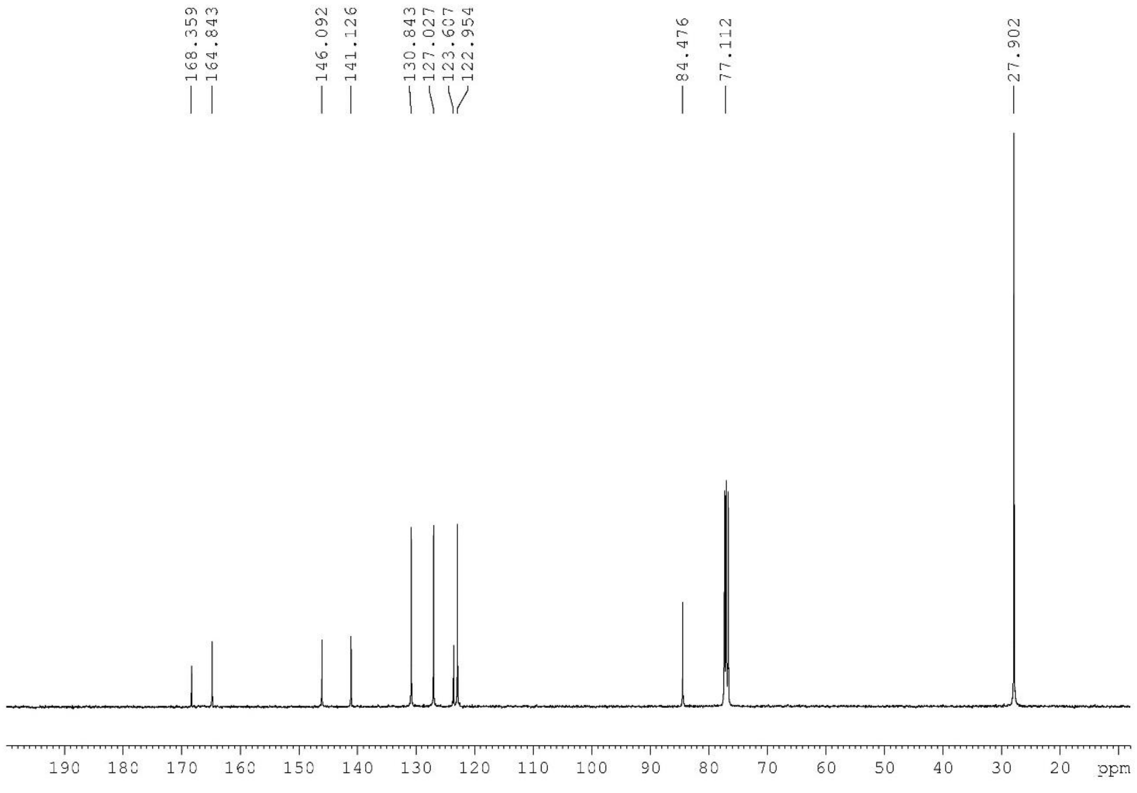

Compound 15f

^{1}H NMR (400 MHz, $CDCl_3$)

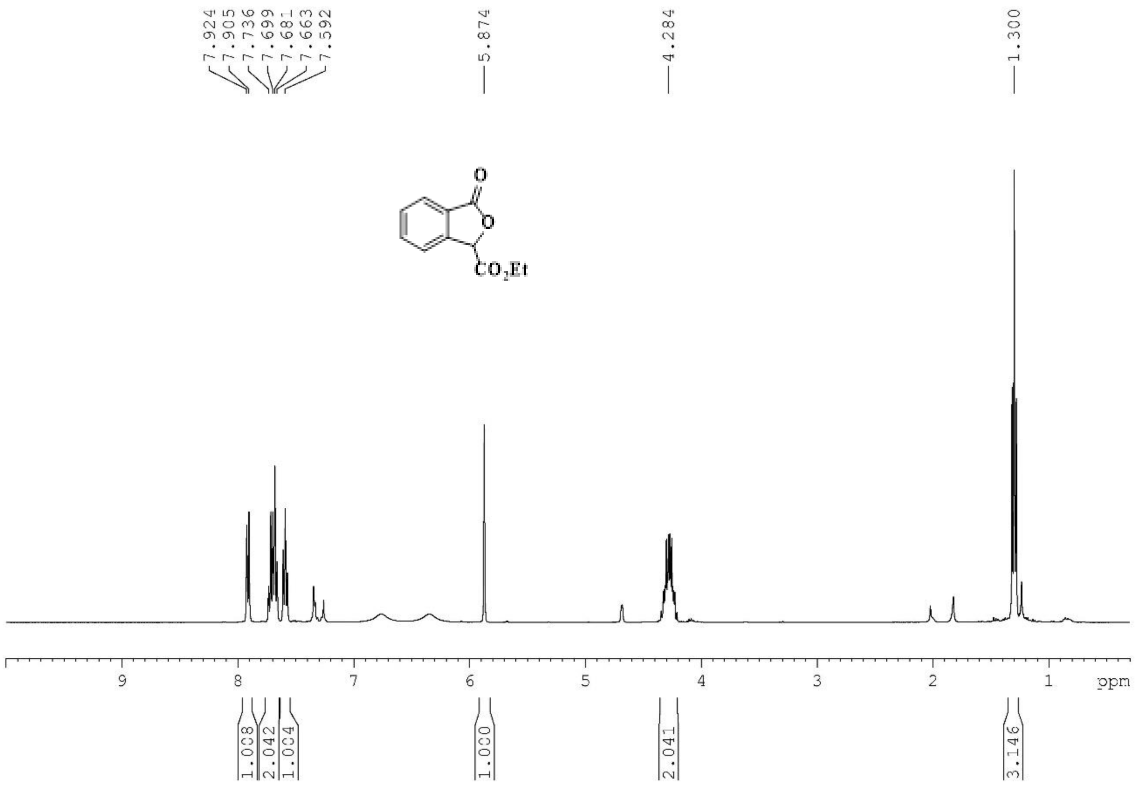

Compound 15g

^{1}H NMR (400 MHz, $CDCl_3$)

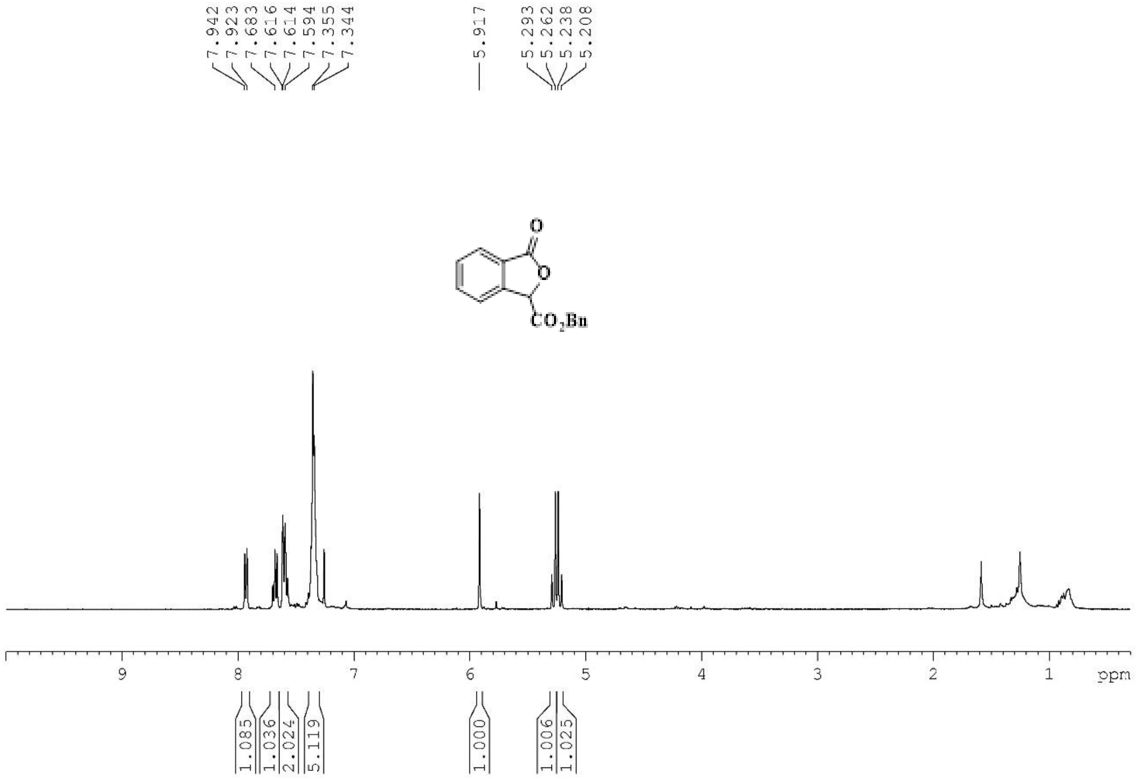

Compound 17aa

^{1}H NMR (400 MHz, $CDCl_3$)

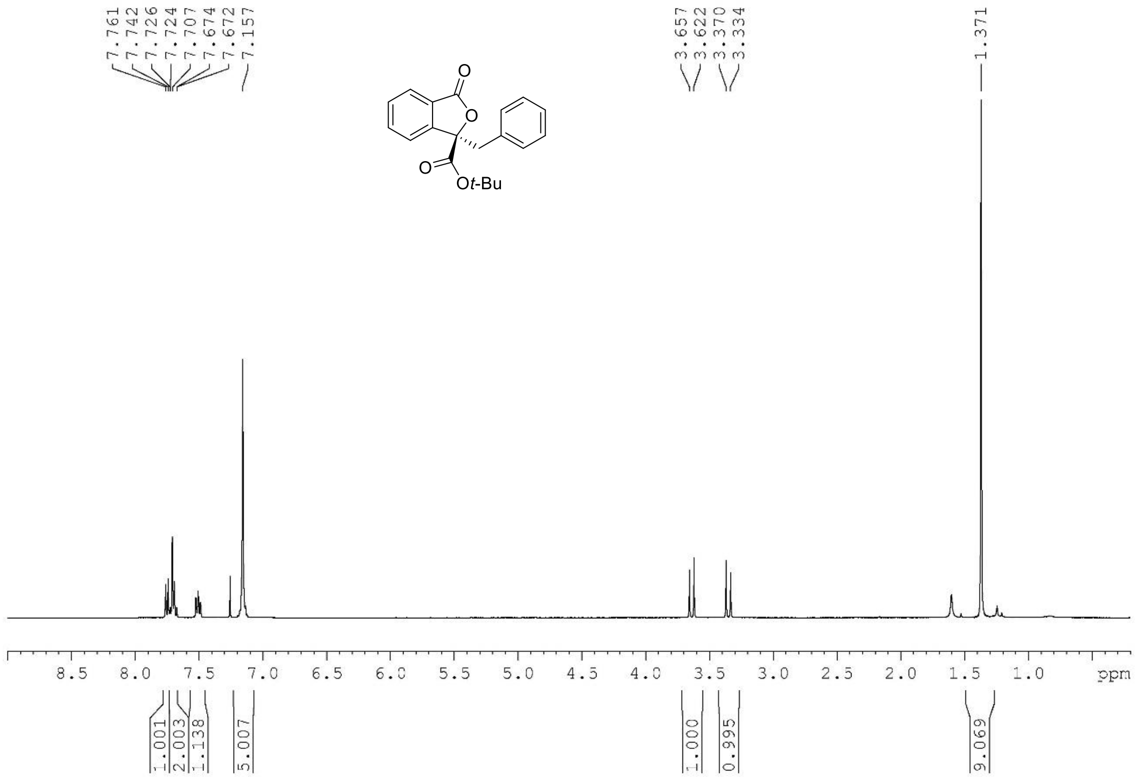

$^{13}C\{^{1}H\}$ NMR (100 MHz, $CDCl_3$)

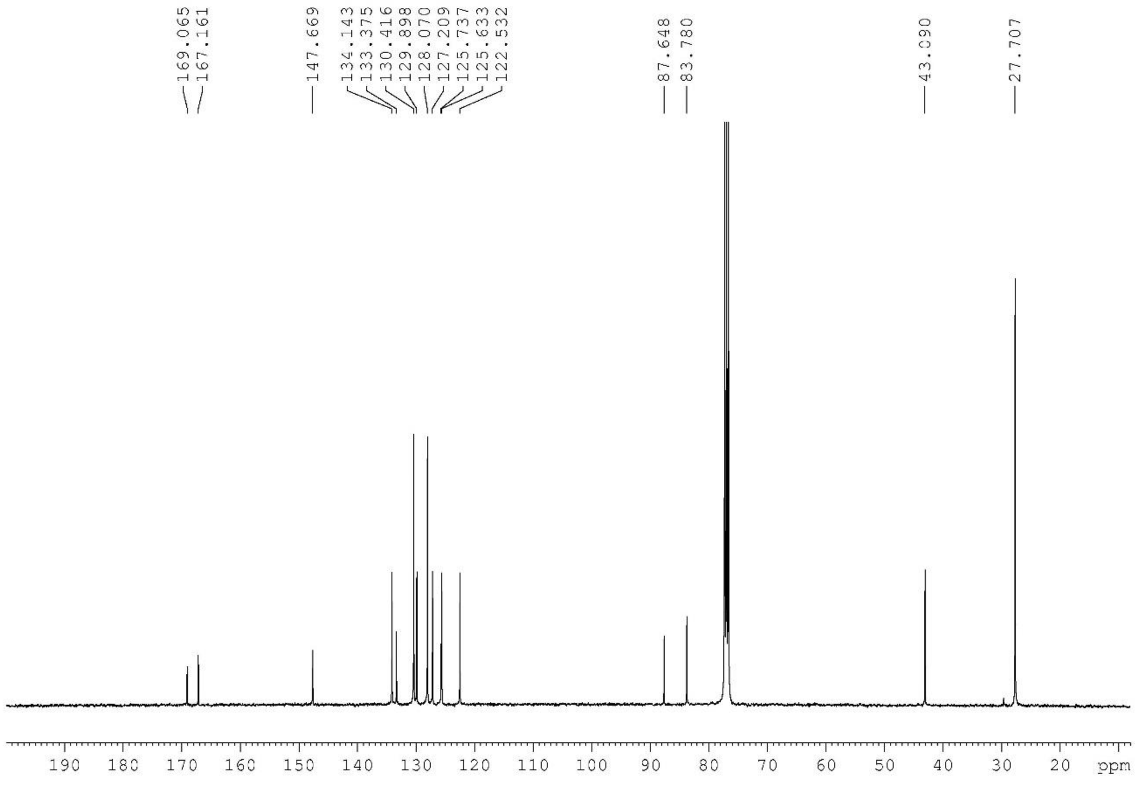

Compound 17ab

^{1}H NMR (400 MHz, $CDCl_3$)

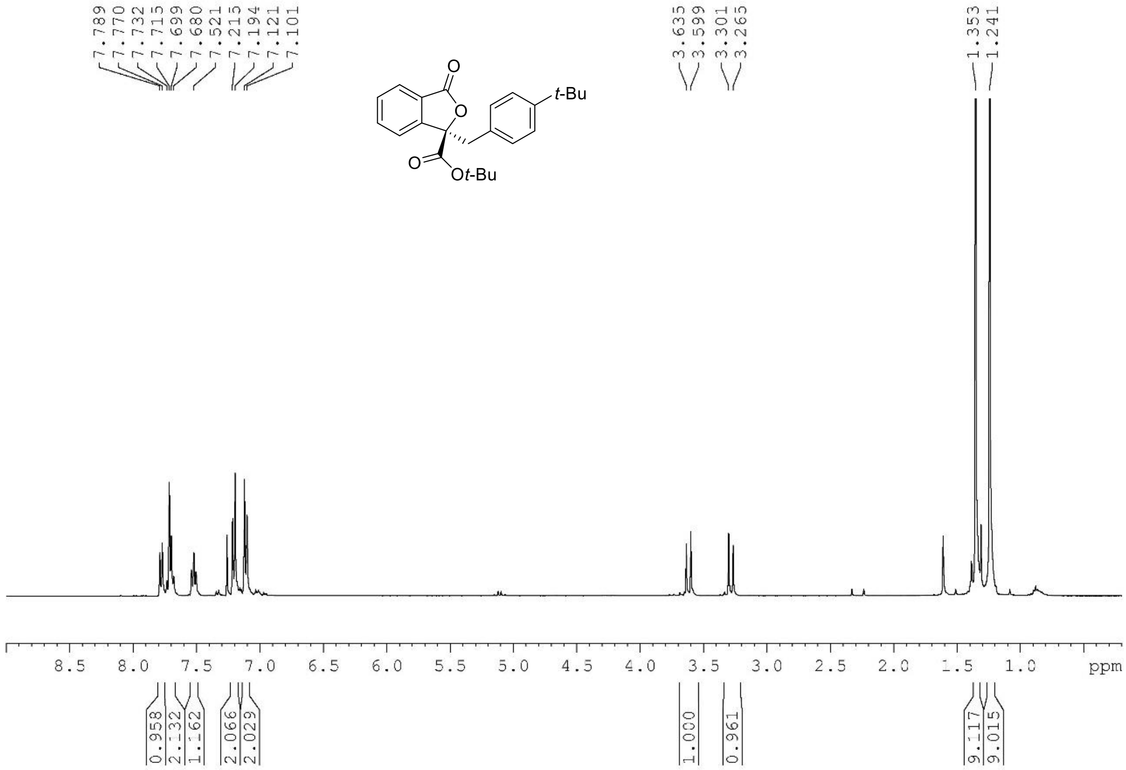

$^{13}C\{^{1}H\}$ NMR (100 MHz, $CDCl_3$)

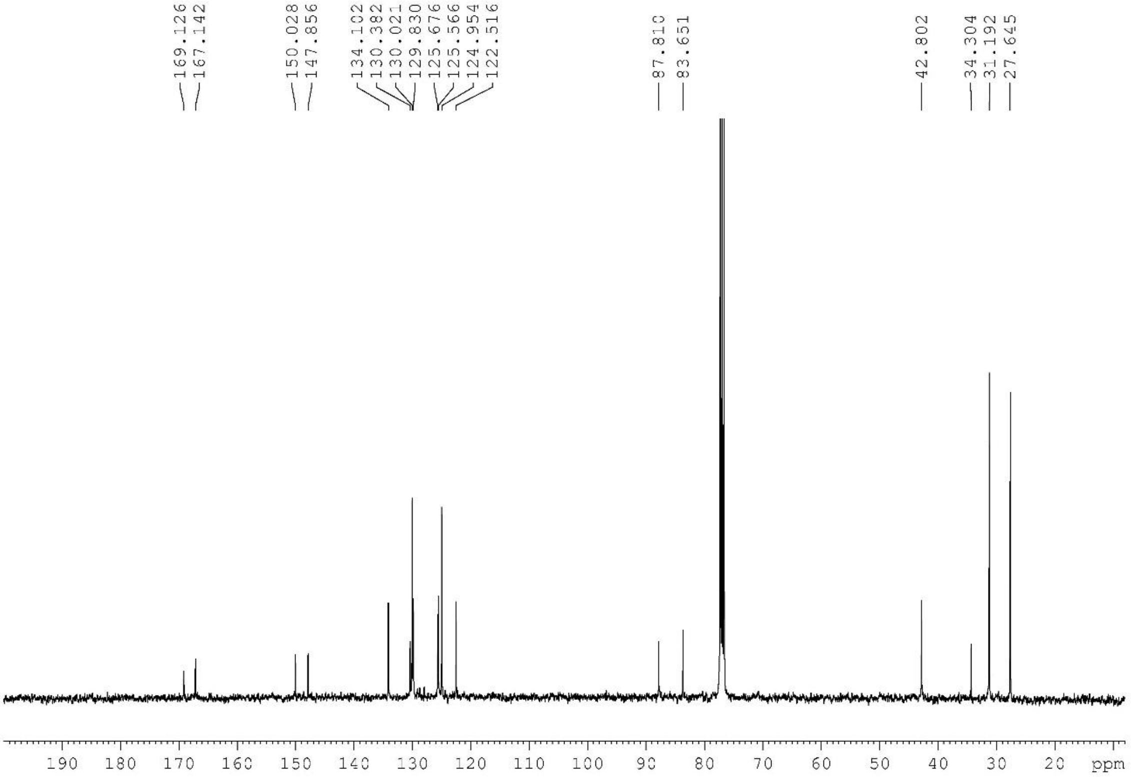

Compound 17ac

^{1}H NMR (400 MHz, $CDCl_3$)

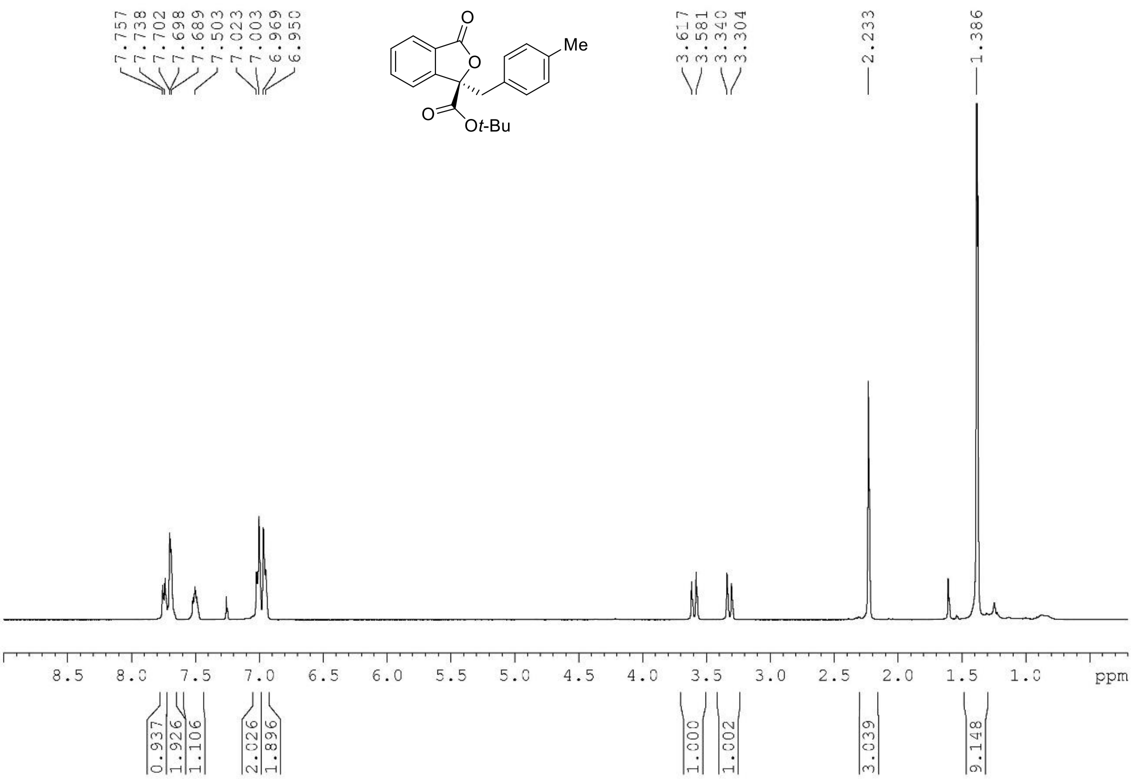

$^{13}C\{^1H\}$ NMR (100 MHz, $CDCl_3$)

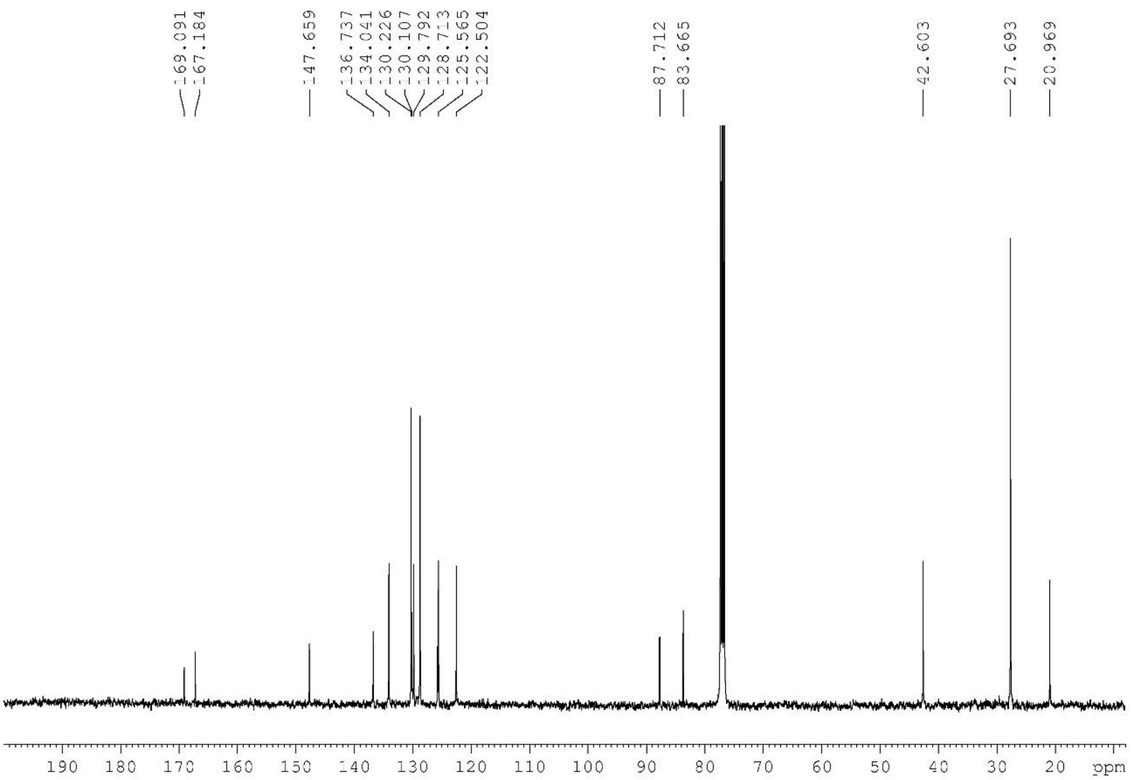

Compound 17ad

^{1}H NMR (400 MHz, $CDCl_3$)

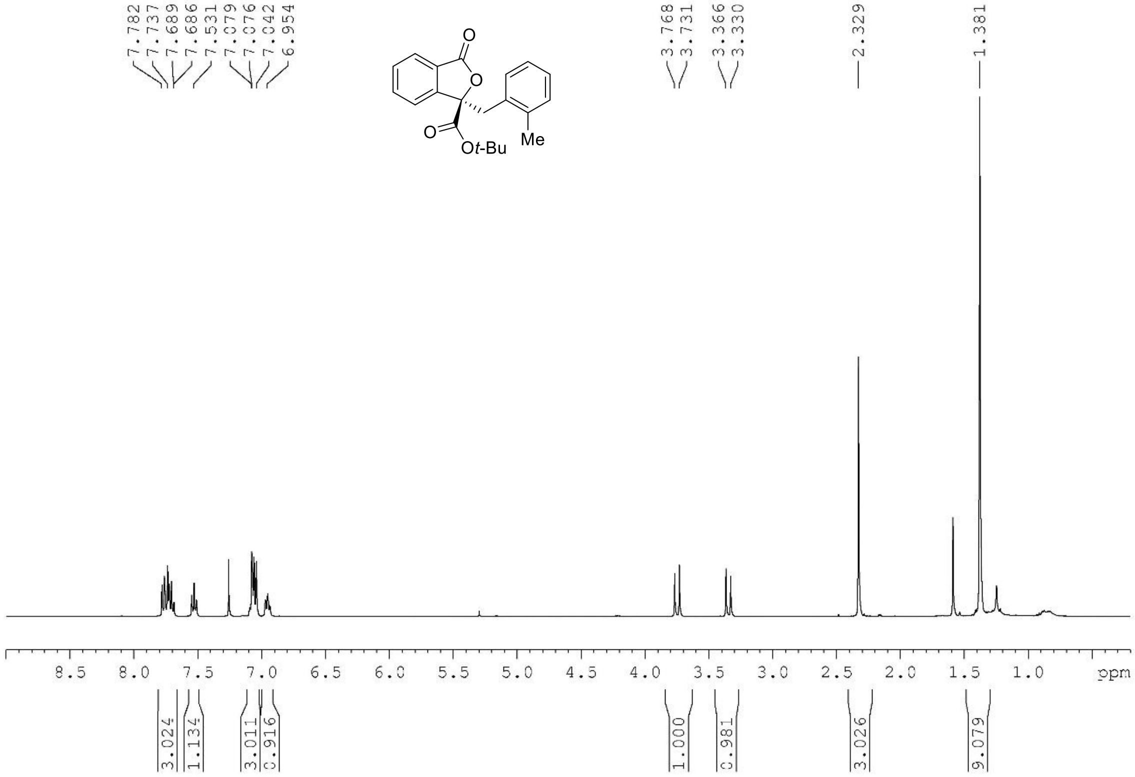

$^{13}C\{^{1}H\}$ NMR (100 MHz, $CDCl_3$)

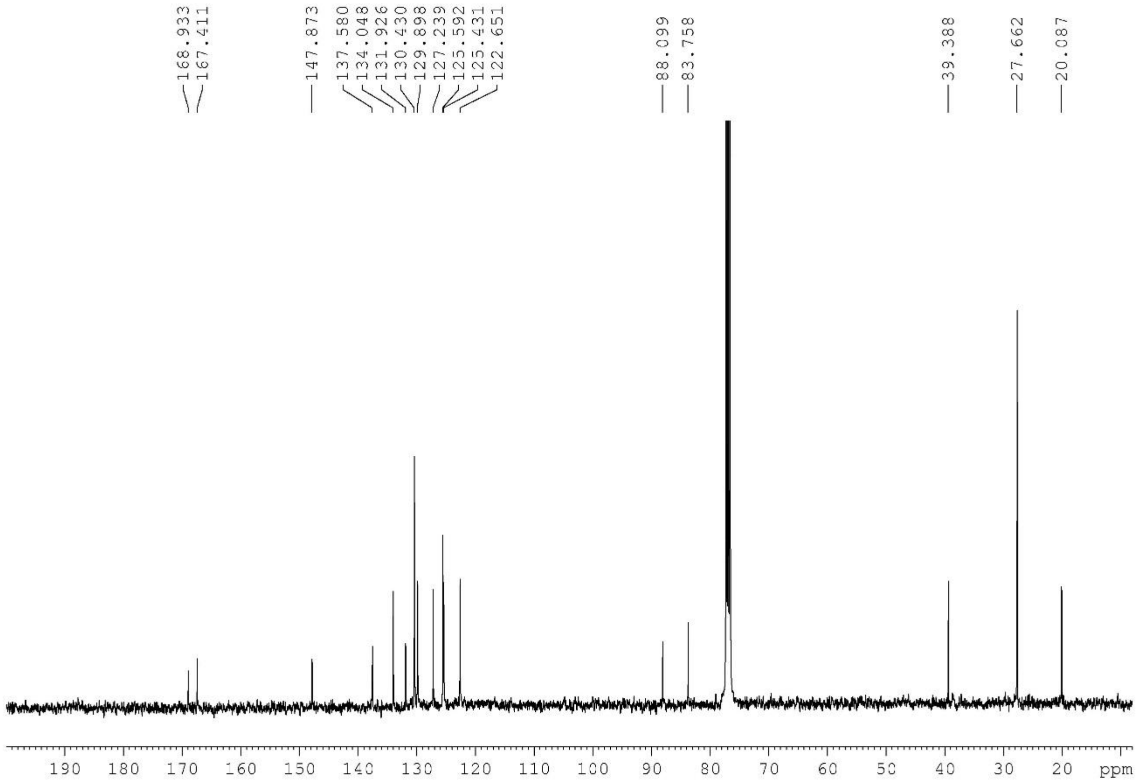

Compound 17ae

^{1}H NMR (600 MHz, $CDCl_3$)

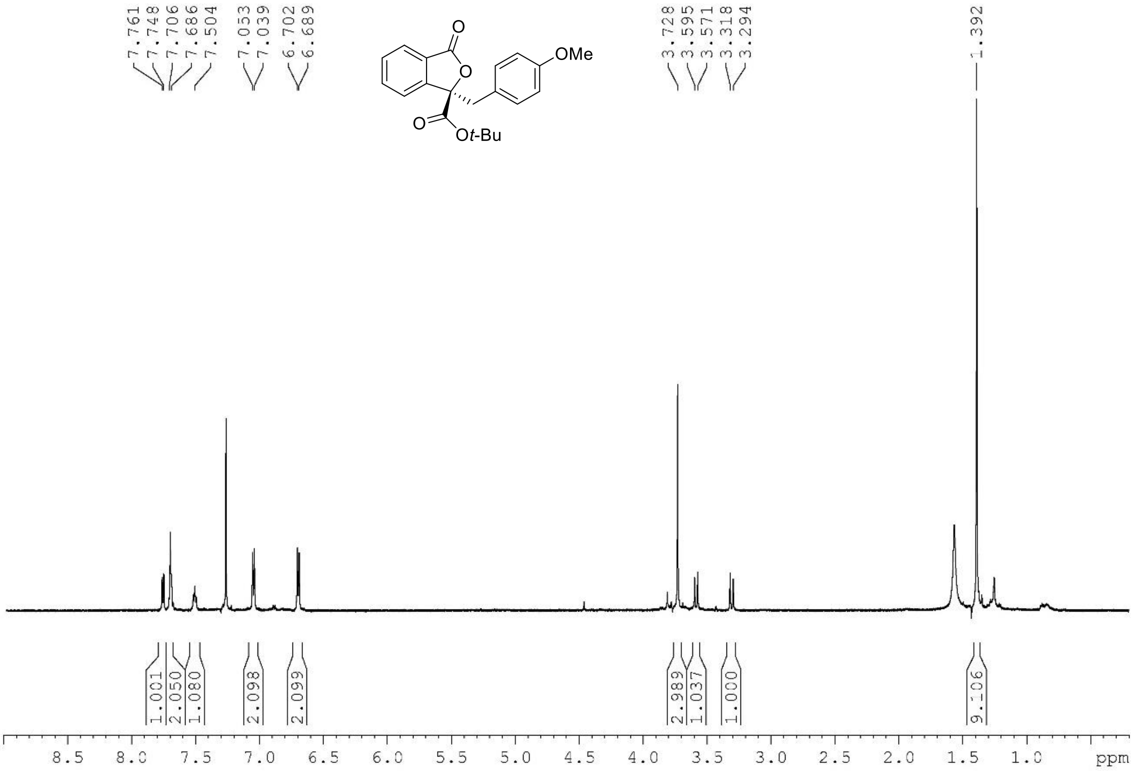

^{13}C{^{1}H} NMR (150 MHz, $CDCl_3$)

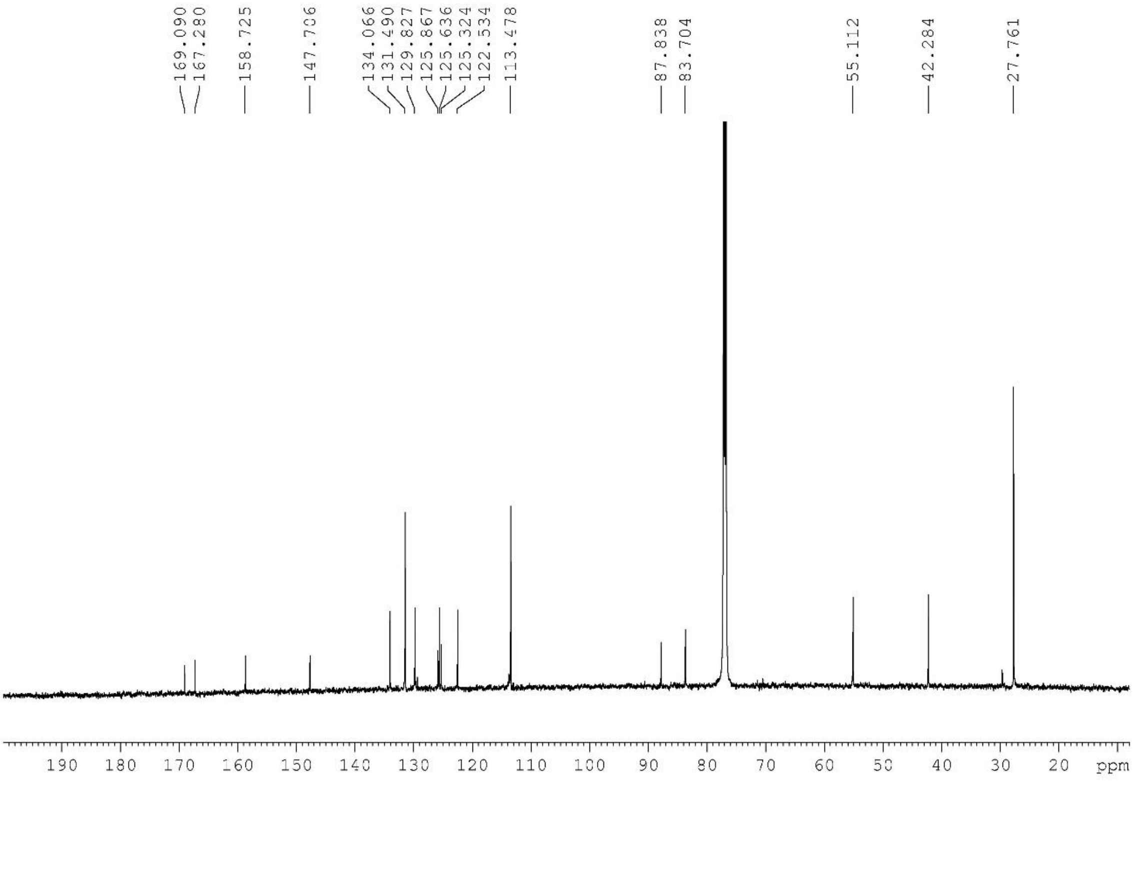

Compound 17af

^{1}H NMR (400 MHz, $CDCl_3$)

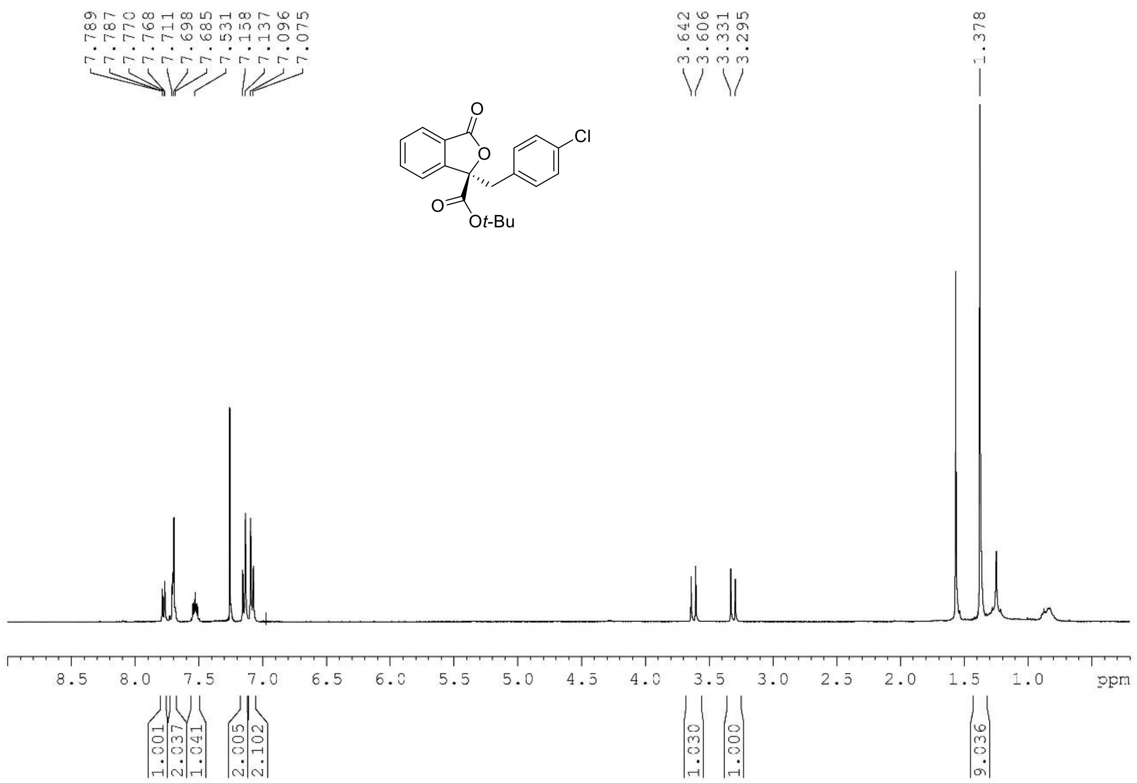

$^{13}C\{^1H\}$ NMR (100 MHz, $CDCl_3$)

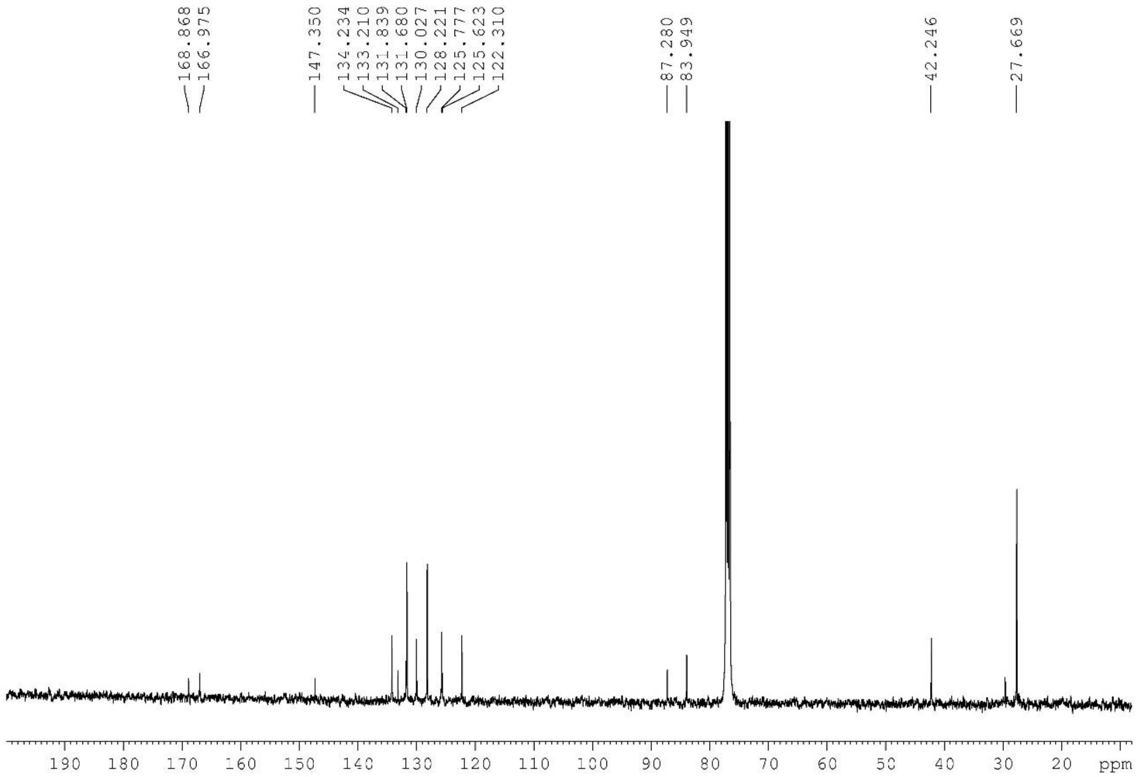

Compound 17ag

^{1}H NMR (400 MHz, $CDCl_3$)

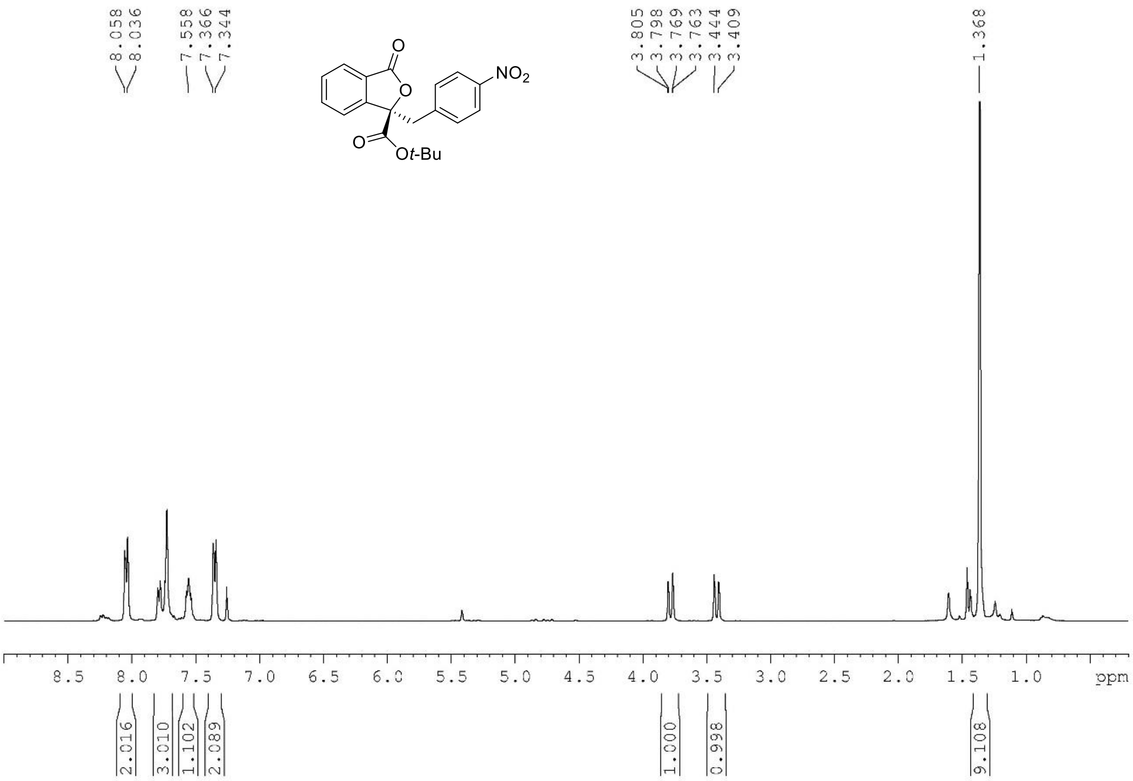

^{13}C{^{1}H} NMR (100 MHz, $CDCl_3$)

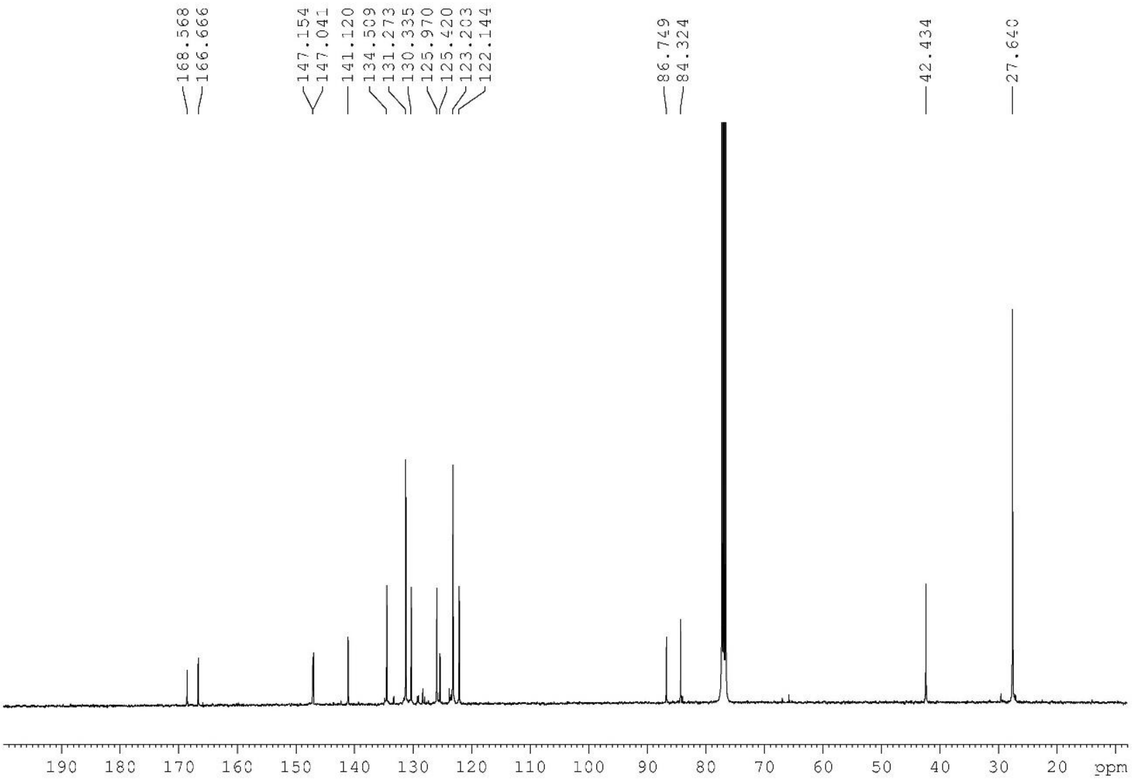

Compound 17ah

^{1}H NMR (400 MHz, $CDCl_3$)

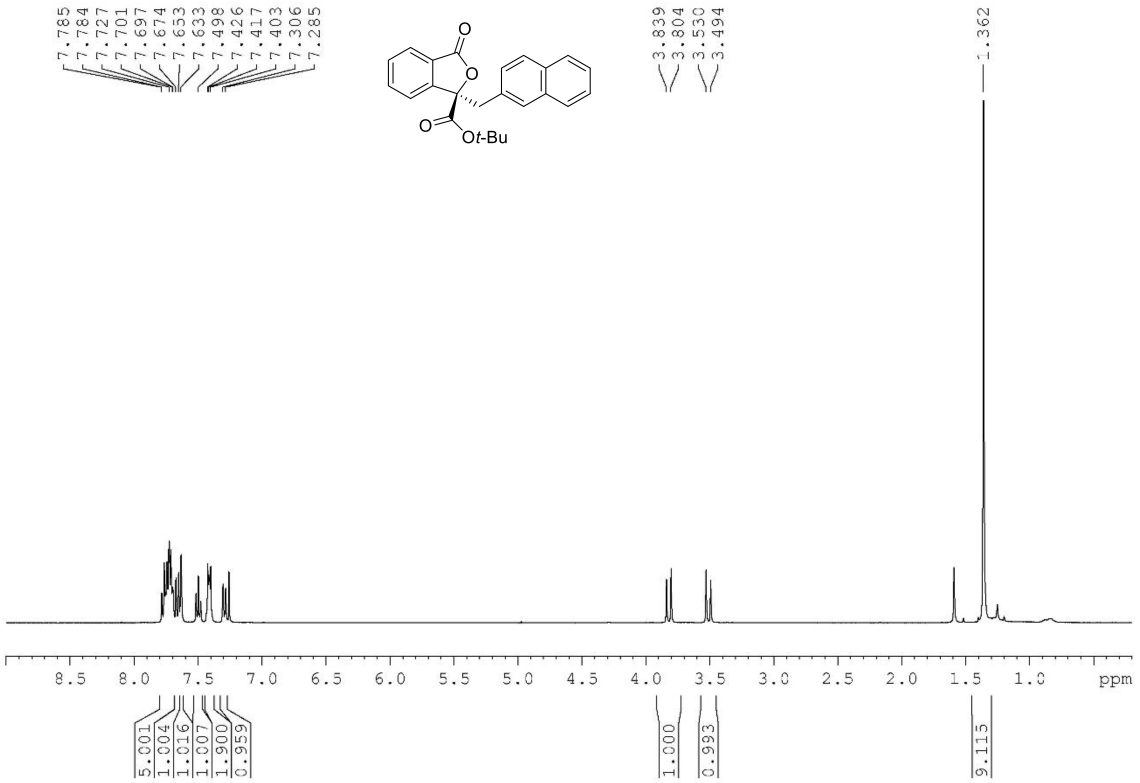

^{13}C{^{1}H} NMR (100 MHz, $CDCl_3$)

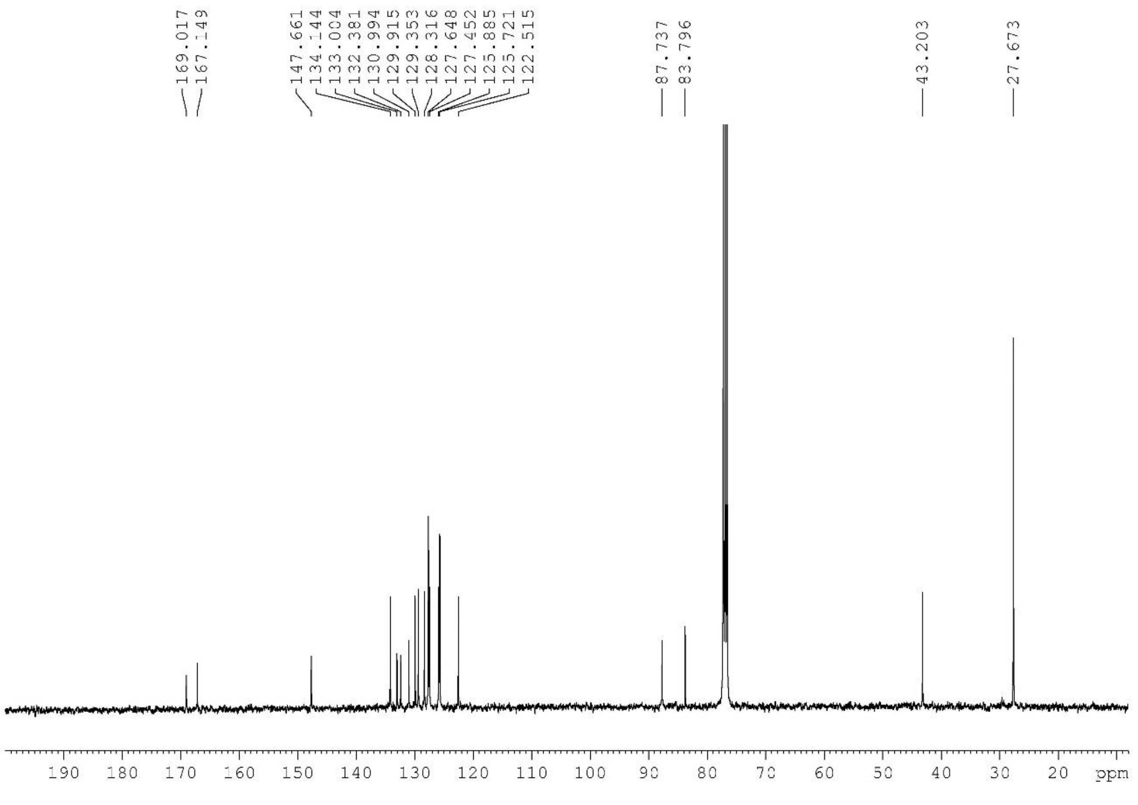

Compound 17ai

^{1}H NMR (400 MHz, $CDCl_3$)

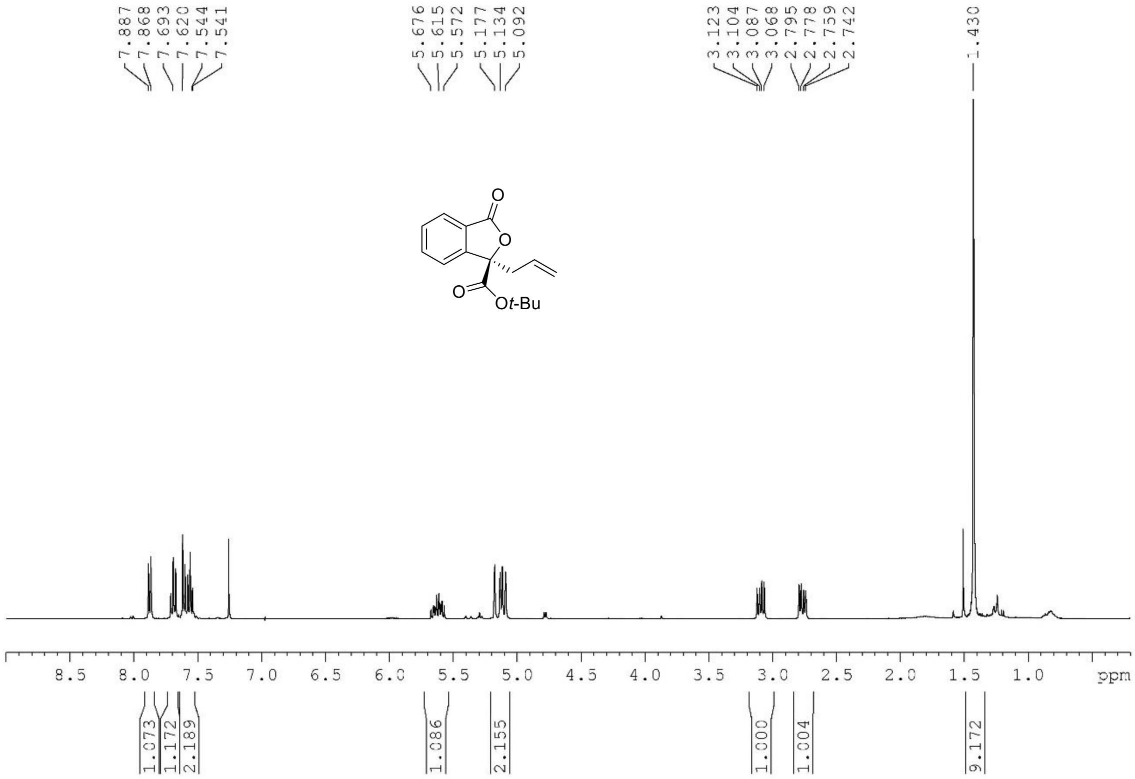

$^{13}C\{^1H\}$ NMR (100 MHz, $CDCl_3$)

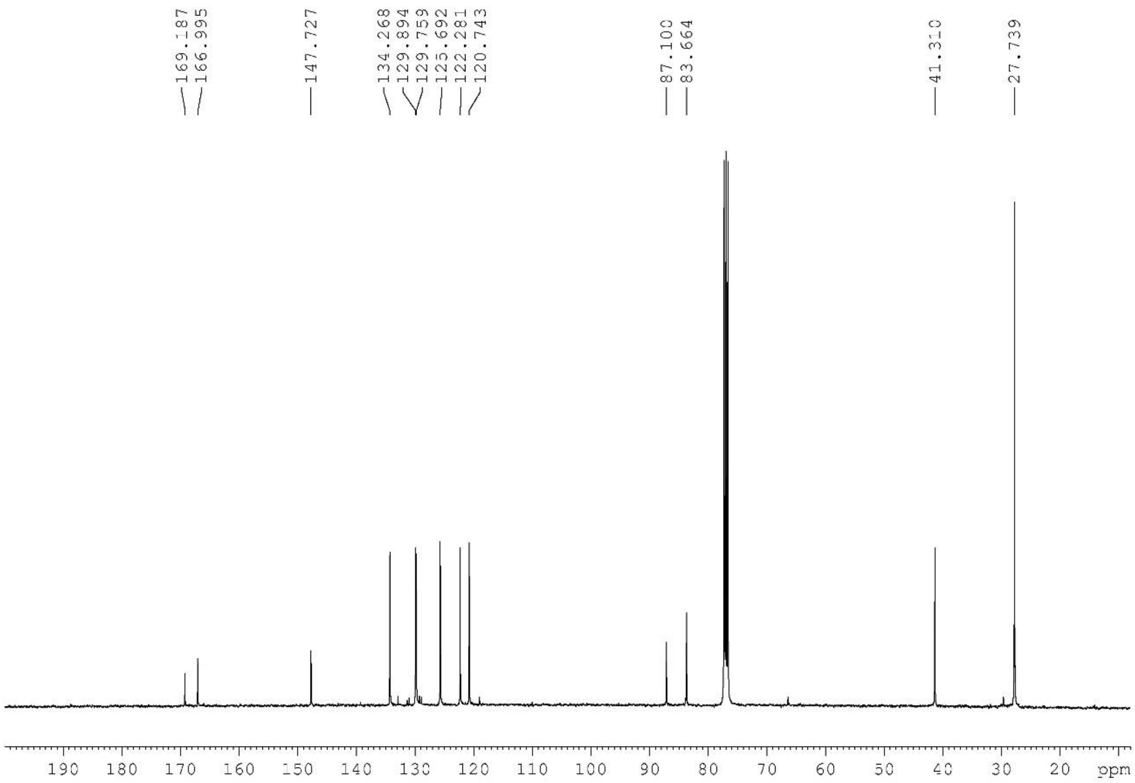

Compound 17ba

^{1}H NMR (400 MHz, $CDCl_3$)

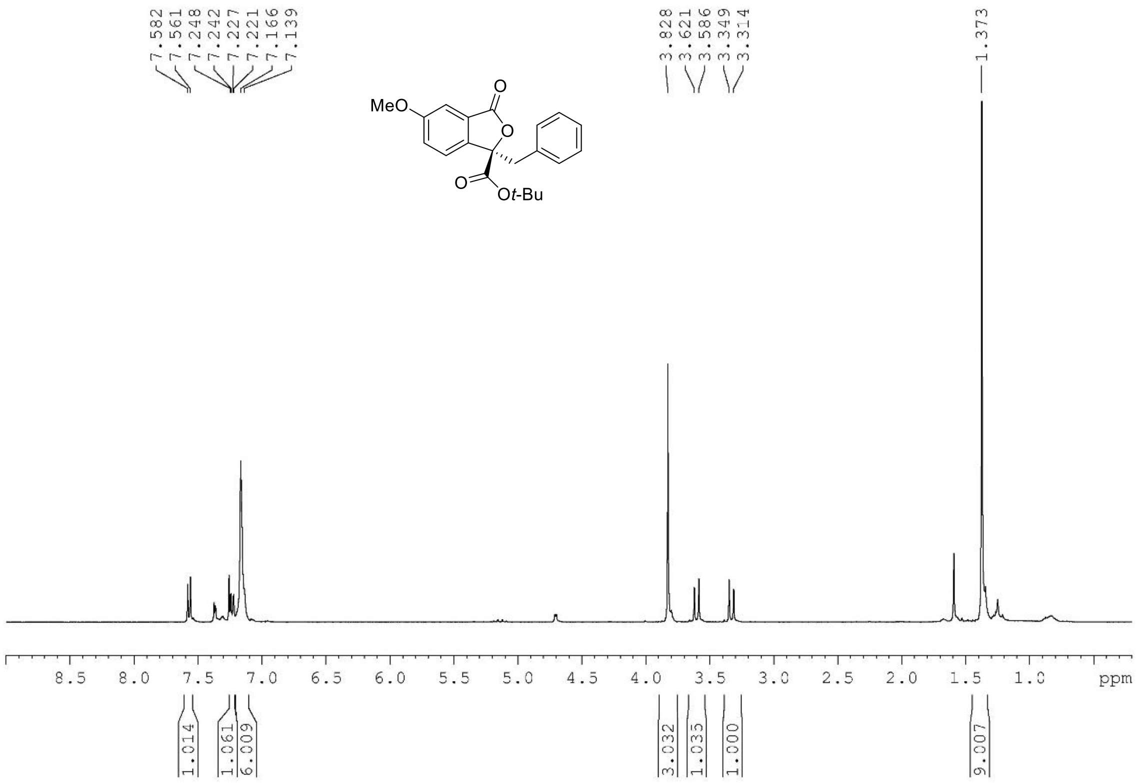

$^{13}C\{^1H\}$ NMR (100 MHz, $CDCl_3$)

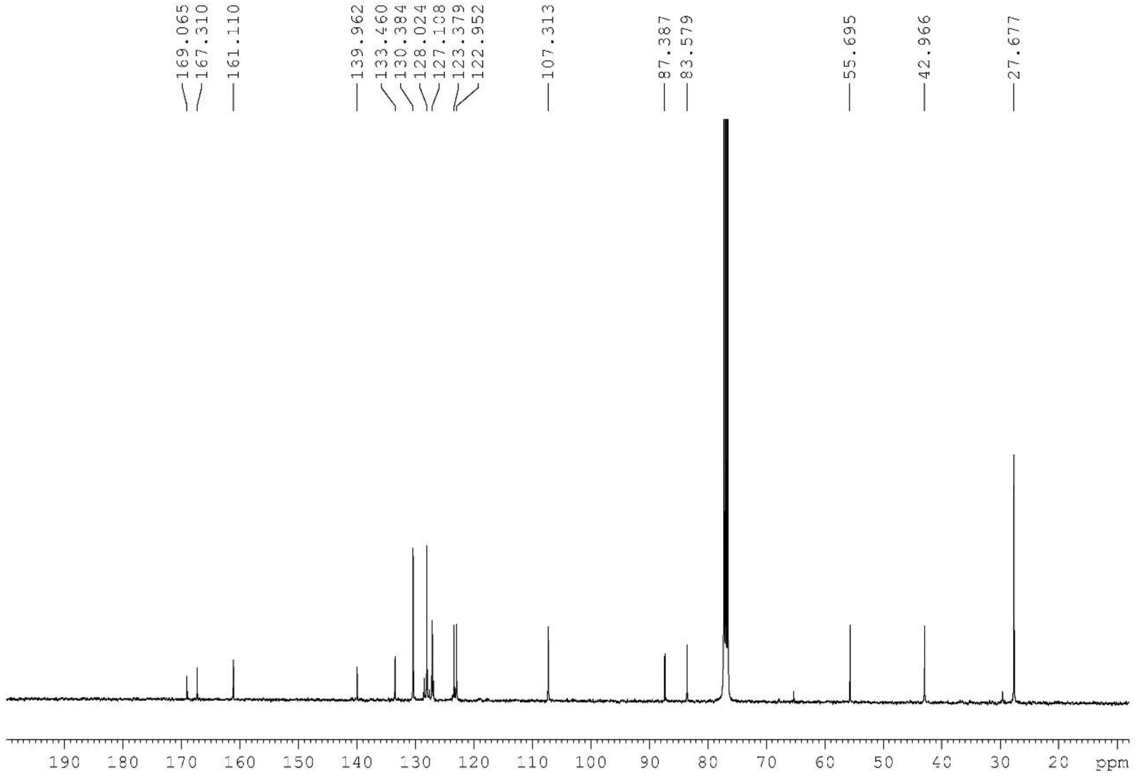

Compound 17ca

^{1}H NMR (600 MHz, $CDCl_3$)

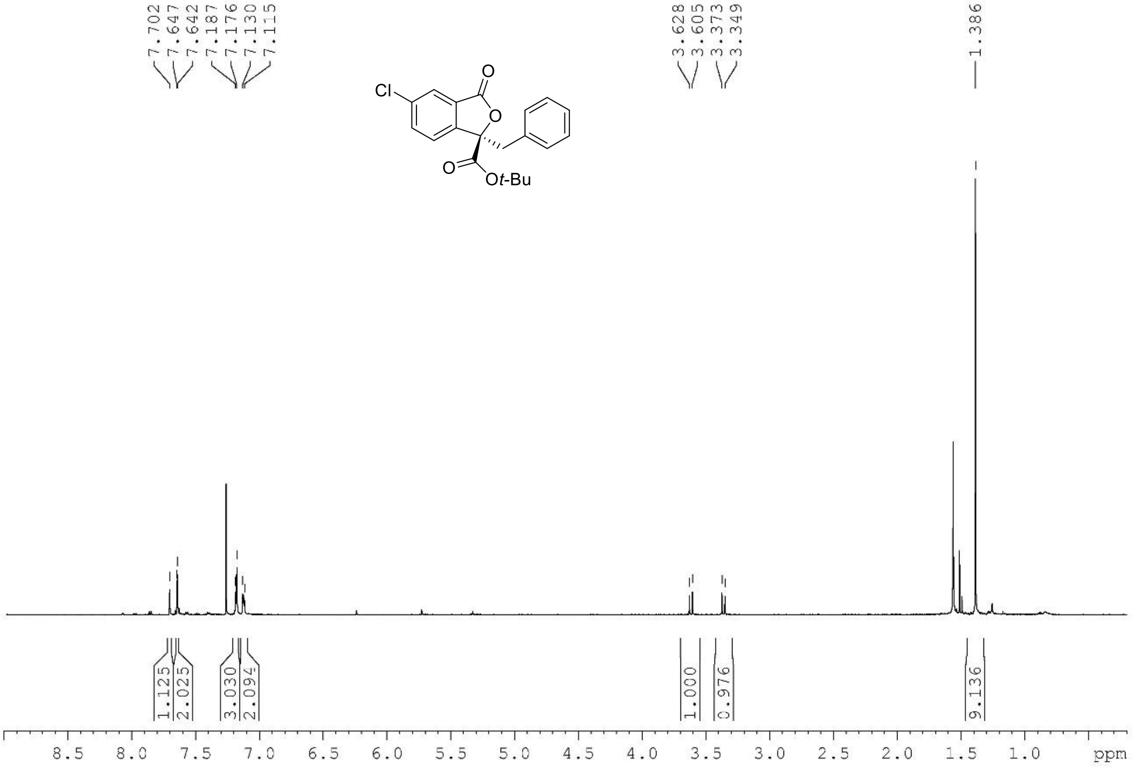

$^{13}C\{^{1}H\}$ NMR (150 MHz, $CDCl_3$)

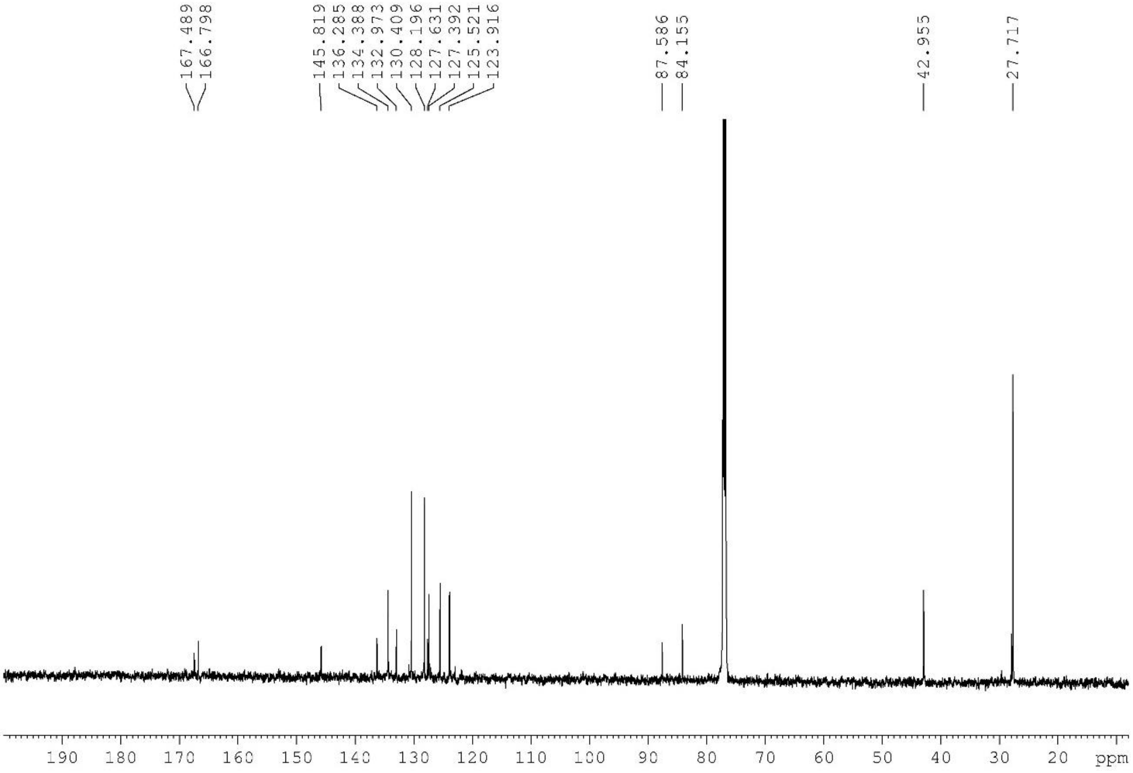

Compound 17da

^{1}H NMR (600 MHz, $CDCl_3$)

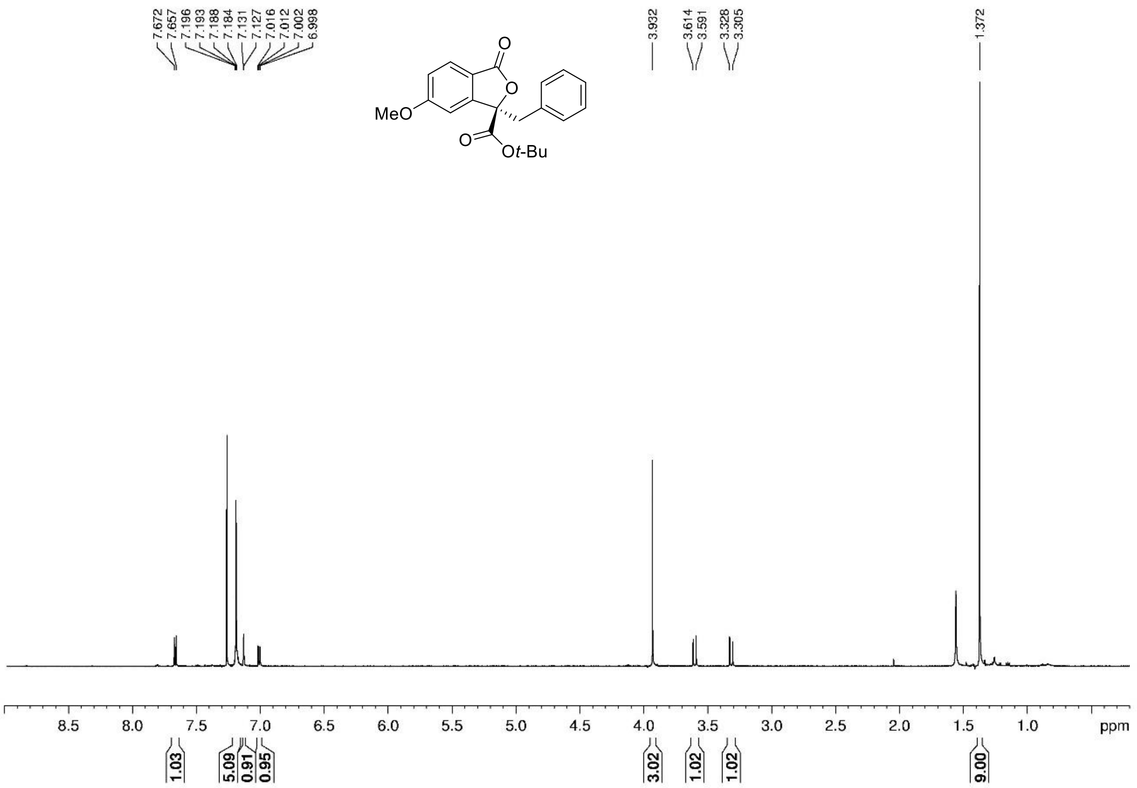

$^{13}C\{^1H\}$ NMR (150 MHz, $CDCl_3$)

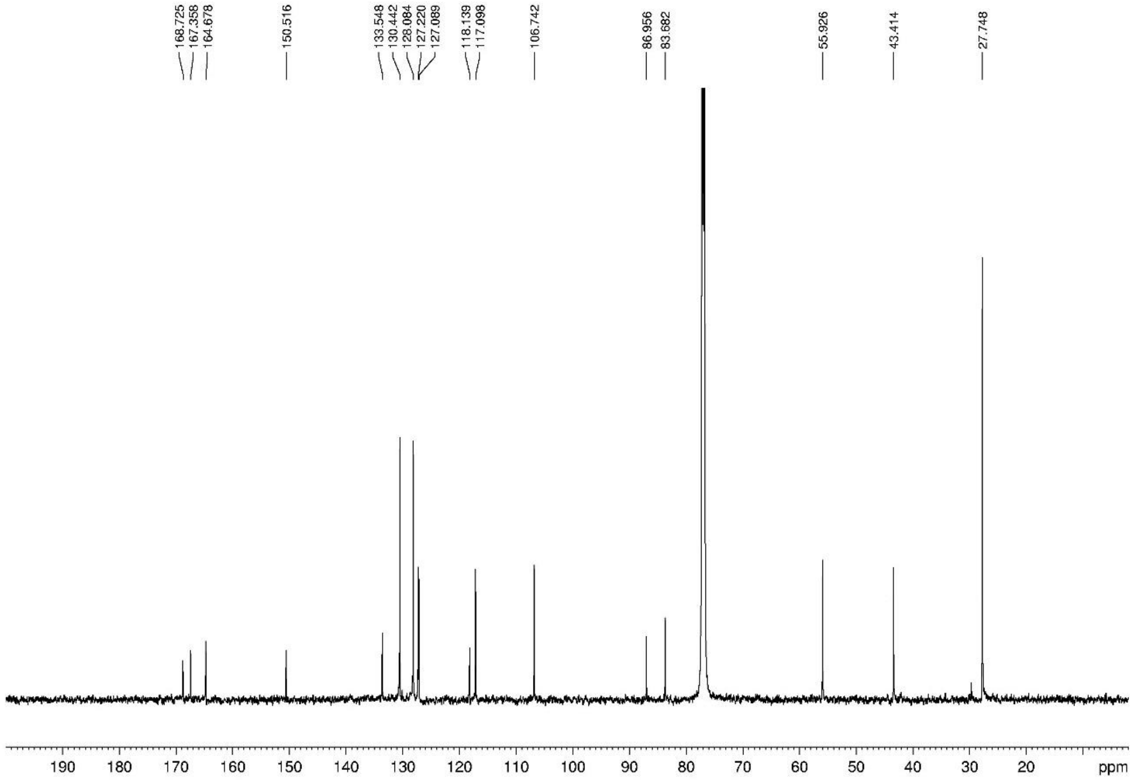

Compound 17ea

^{1}H NMR (600 MHz, $CDCl_3$)

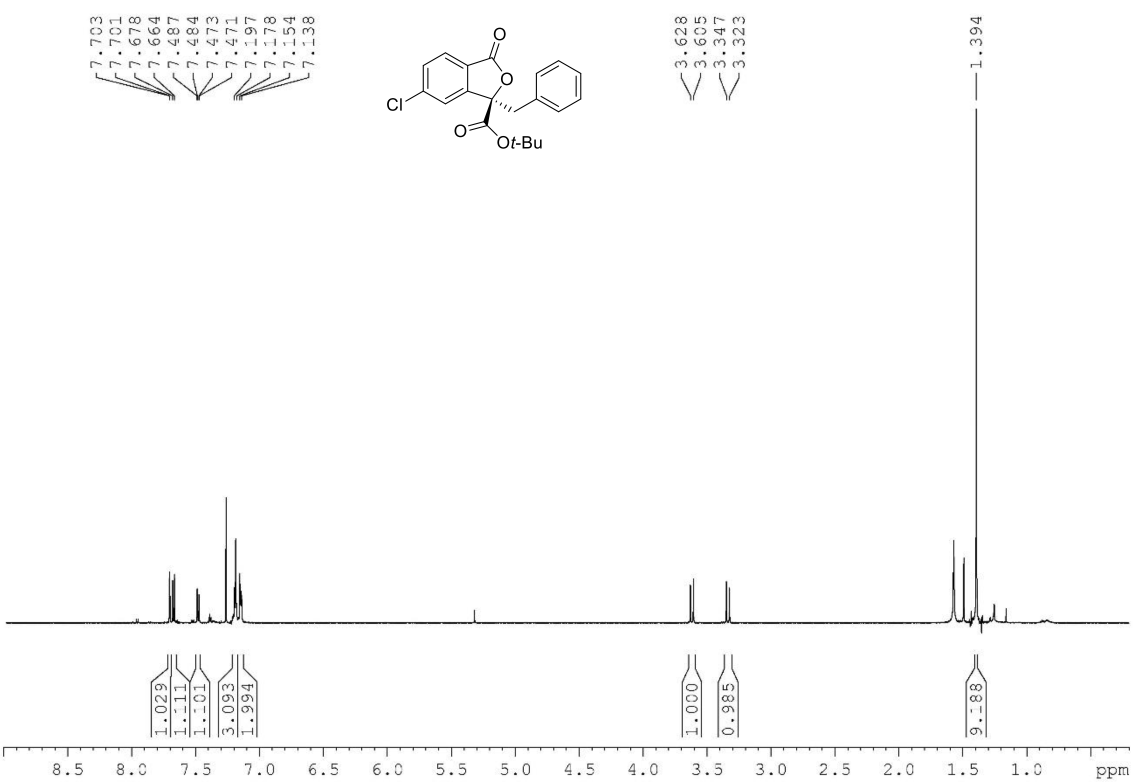

$^{13}C\{^{1}H\}$ NMR (150 MHz, $CDCl_3$)

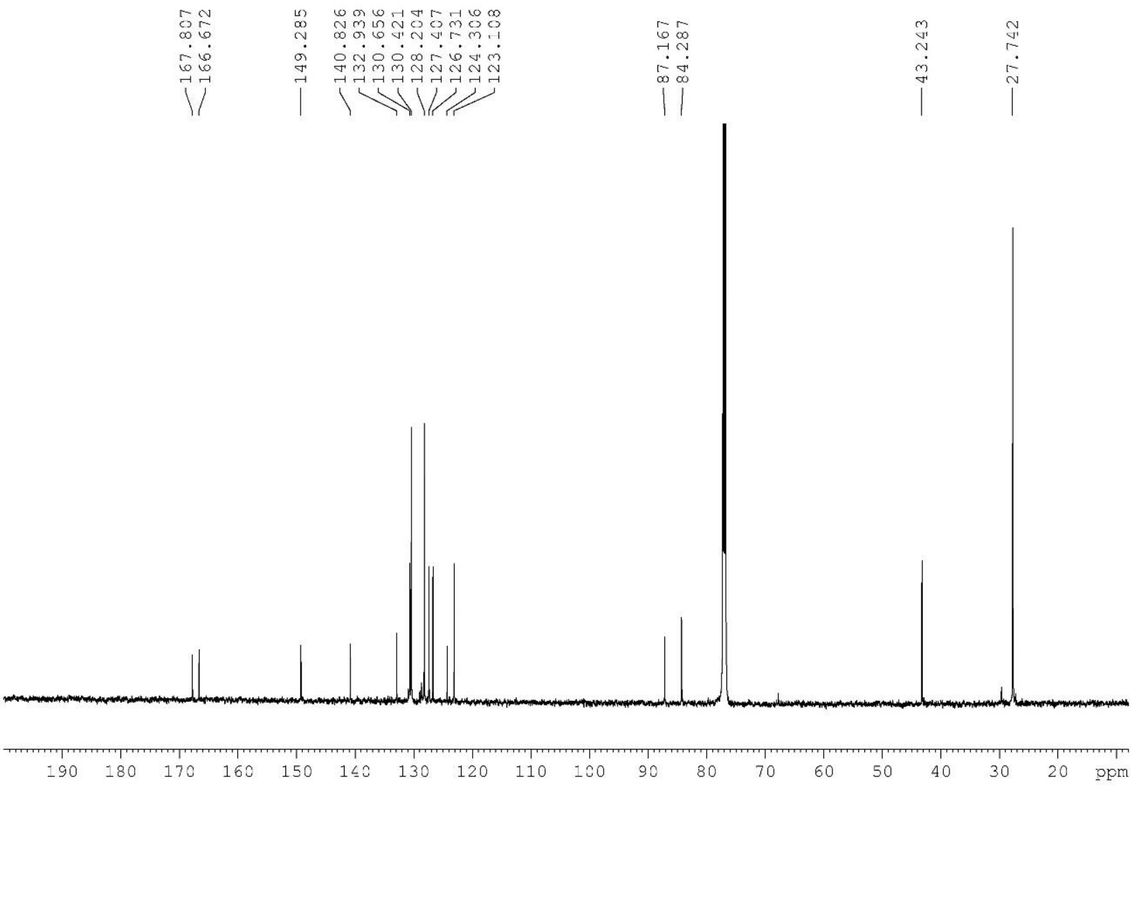

Compound 18

^{1}H NMR (400 MHz, $CDCl_3$)

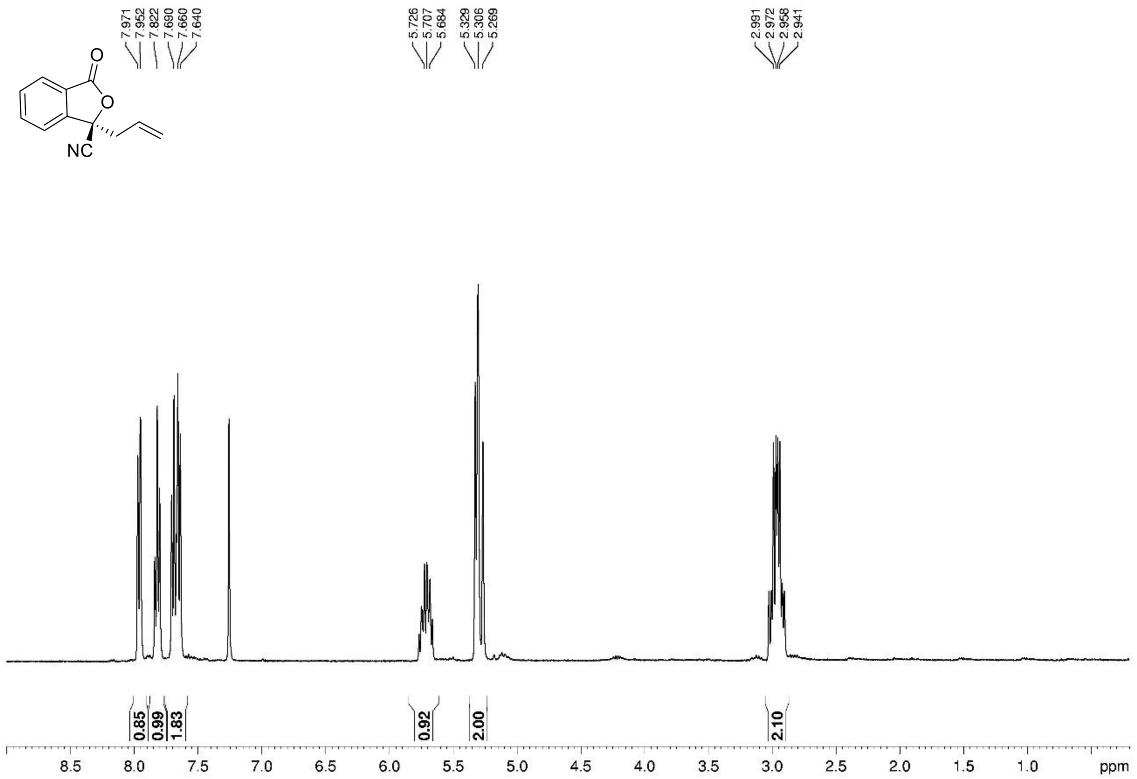

^{13}C NMR (100 MHz, $CDCl_3$)

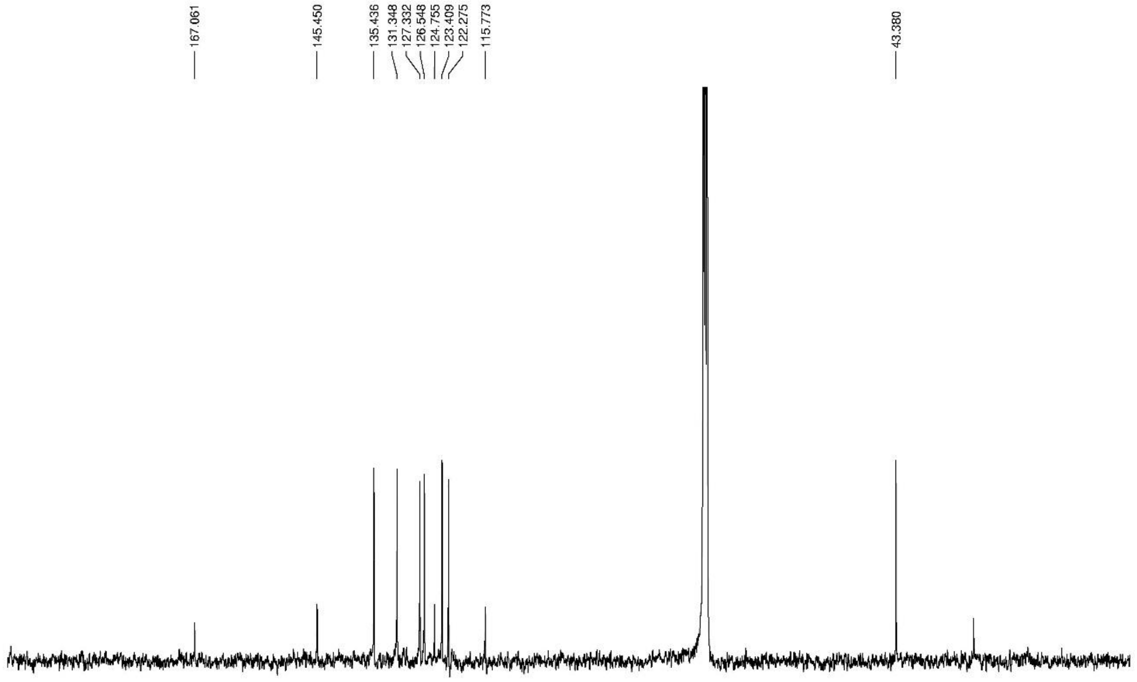

Compound 19

^{1}H NMR (400 MHz, $CDCl_3$)

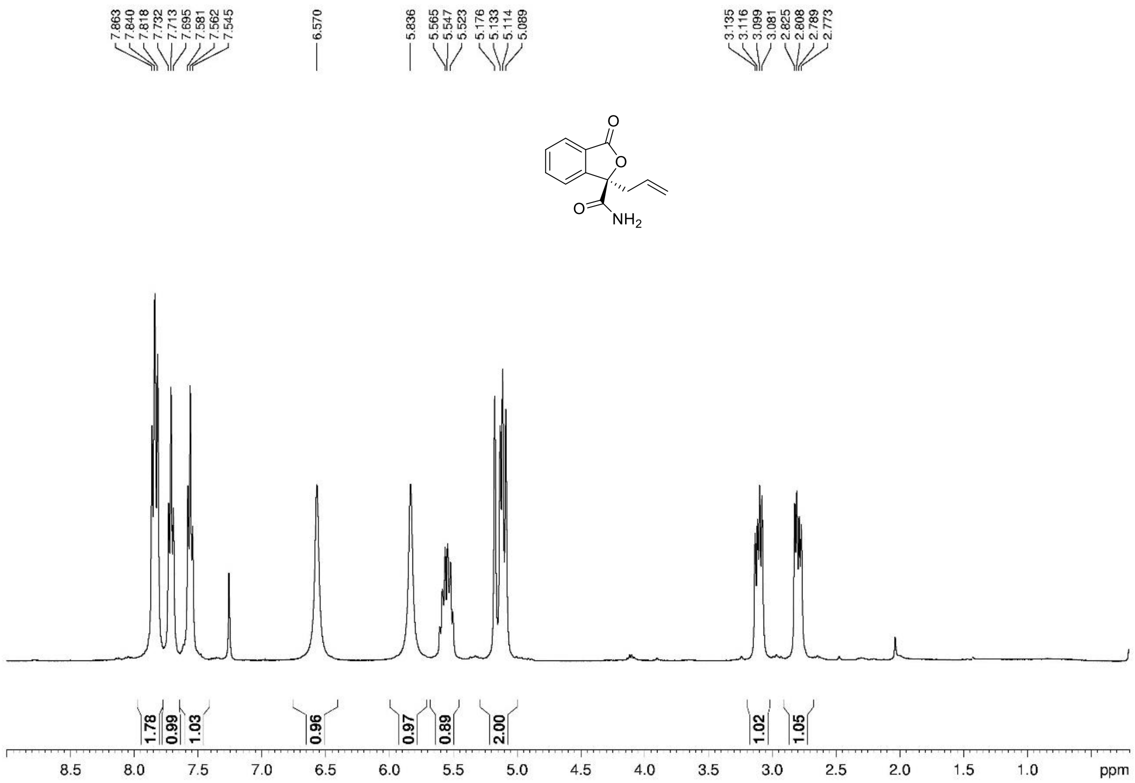

$^{13}C\{^{1}H\}$ NMR (100 MHz, $CDCl_3$)

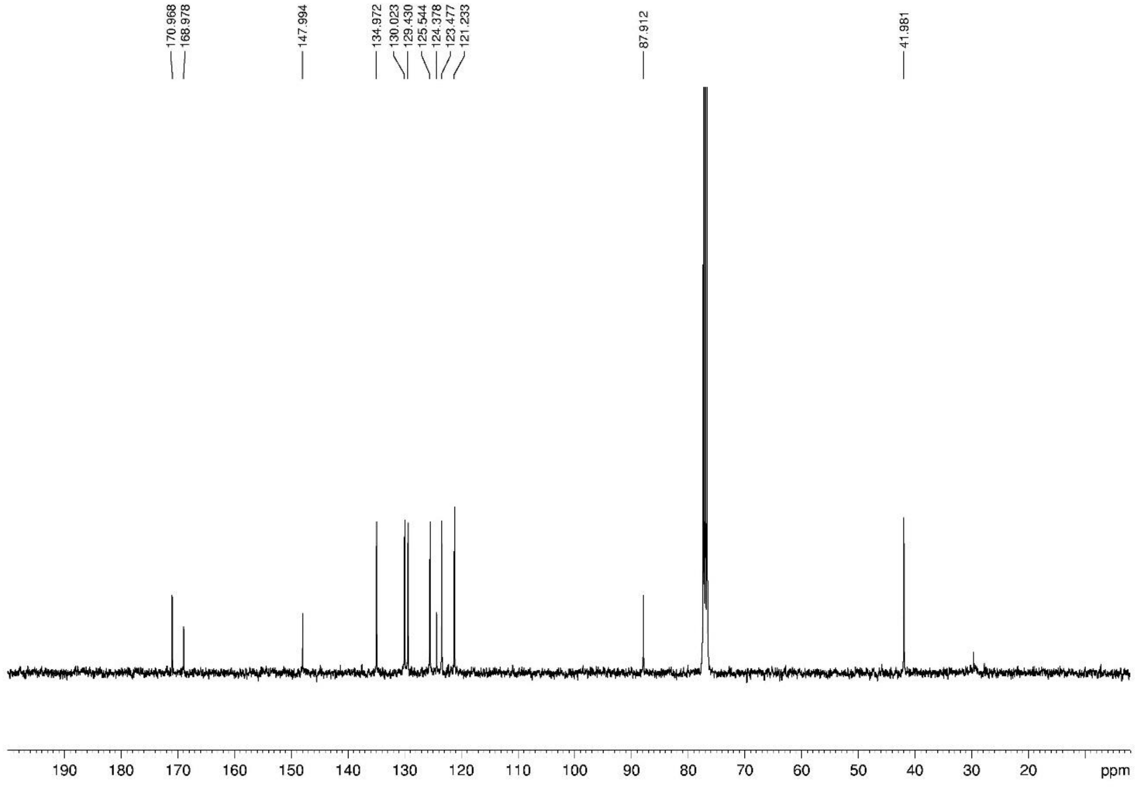

Copies of HPLC traces

Compound 17aa

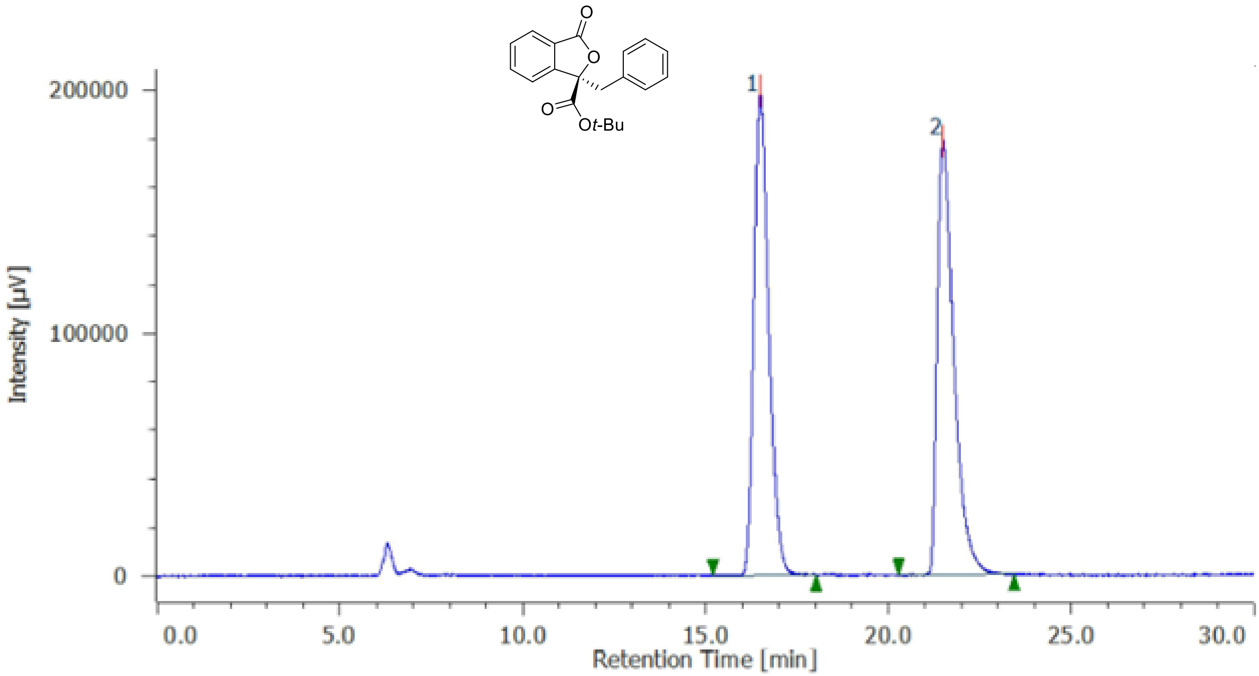

#	Peak Name	CH	tR [min]	Area [μV·sec]	Height [μV]	Area%	Height%	Quantity	NTP	Resolution	Symmetry Factor	Warning
1	Unknown	9	16.492	5583481	198382	49.569	52.626	N/A	8062	6.506	1.324	
2	Unknown	9	21.494	5680584	178585	50.431	47.374	N/A	11346	N/A	1.811	

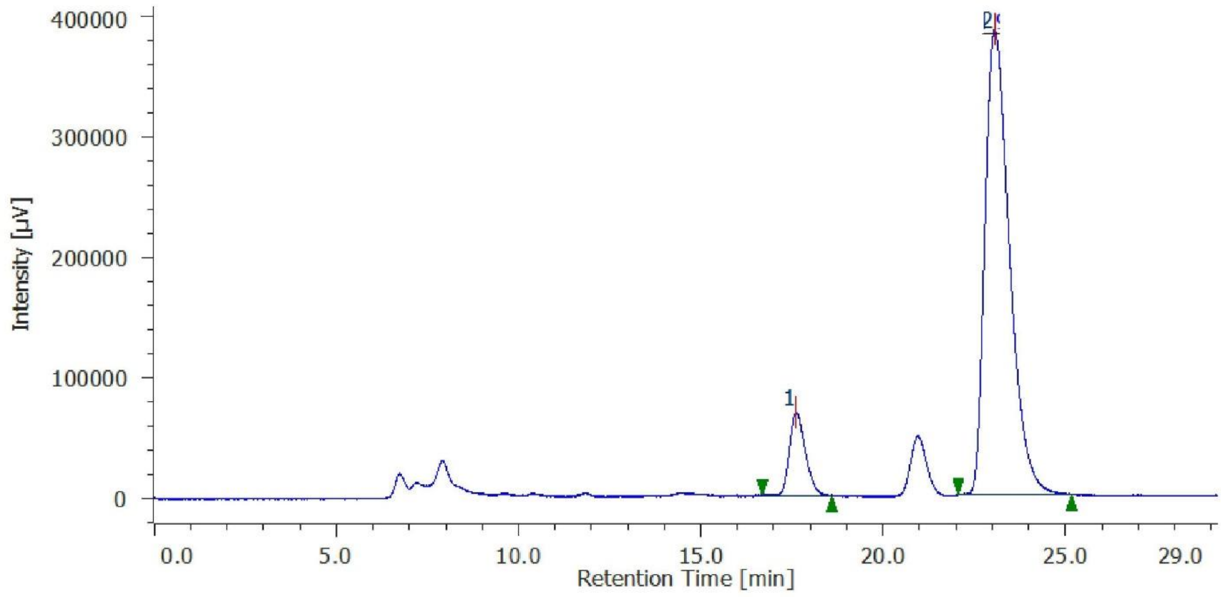

#	Peak Name	CH	tR [min]	Area [μV·sec]	Height [μV]	Area%	Height%	Quantity	NTP	Resolution	Symmetry Factor	Warning
1	Unknown	9	17.618	2083311	69463	10.332	15.248	N/A	7950	5.426	1.274	
2	Unknown	9	23.060	18080367	386085	89.668	84.752	N/A	5708	N/A	1.502	

Compound 17ab

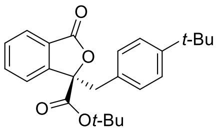

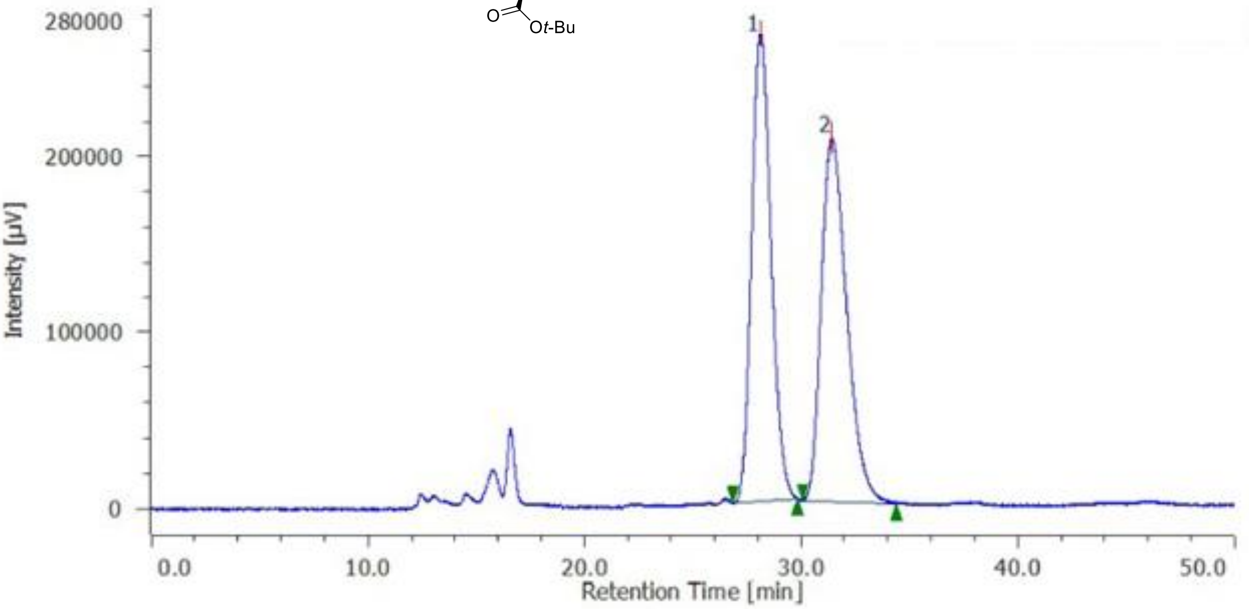

#	Peak Name	CH	tR [min]	Area [µV·sec]	Height [µV]	Area%	Height%	Quantity	NTP	Resolution	Symmetry Factor	Warning
1	Unknown	9	28.114	16569920	266288	49.585	56.357	N/A	4656	1.737	1.186	
2	Unknown	9	31.400	16847612	206215	50.415	43.643	N/A	3428	N/A	1.391	

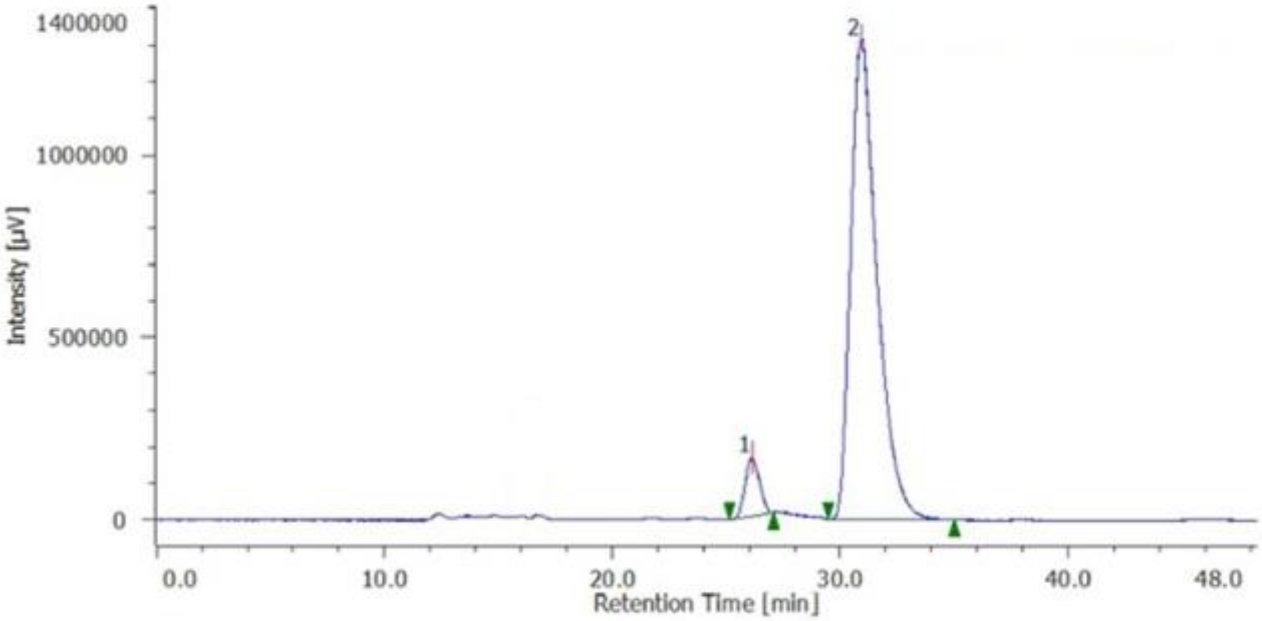

#	Peak Name	CH	tR [min]	Area [µV·sec]	Height [µV]	Area%	Height%	Quantity	NTP	Resolution	Symmetry Factor	Warning
1	Unknown	9	26.113	7095073	159599	6.297	10.694	N/A	7332	2.944	1.069	
2	Unknown	9	30.888	105585853	1332751	93.703	89.306	N/A	3695	N/A	1.549	

Compound 17ac

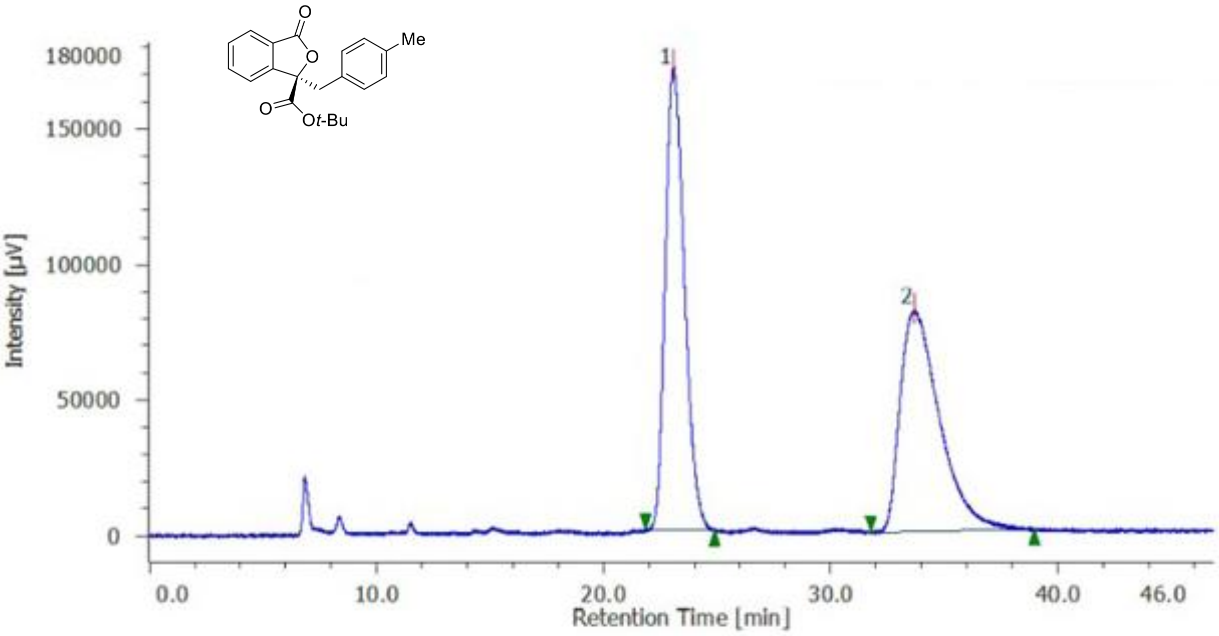

#	Peak Name	CH	tR [min]	Area [µV·sec]	Height [µV]	Area%	Height%	Quantity	NTP	Resolution	Symmetry Factor	Warning
1	Unknown	9	23.066	9955092	170871	50.452	67.623	N/A	3519	4.570	1.277	
2	Unknown	9	33.670	9776812	81812	49.548	32.377	N/A	1890	N/A	1.788	

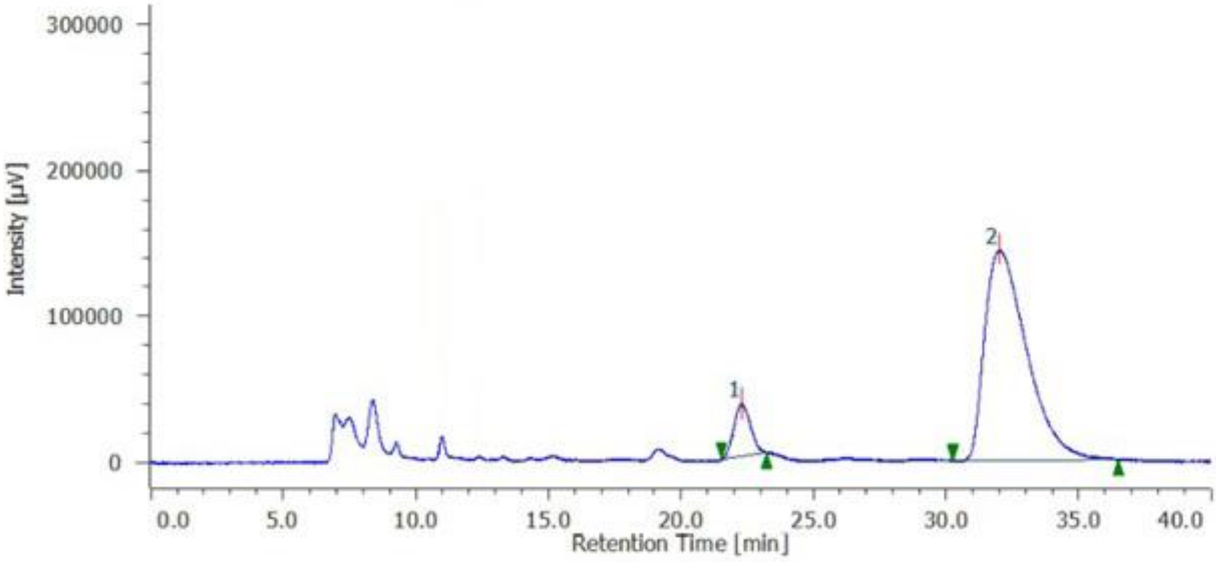

#	Peak Name	CH	tR [min]	Area [µV·sec]	Height [µV]	Area%	Height%	Quantity	NTP	Resolution	Symmetry Factor	Warning
1	Unknown	9	22.286	1546939	36102	8.942	20.012	N/A	5773	4.797	1.125	
2	Unknown	9	32.011	15752213	144304	91.058	79.988	N/A	1960	N/A	1.774	

Compound 17ad

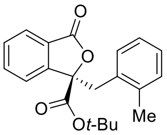

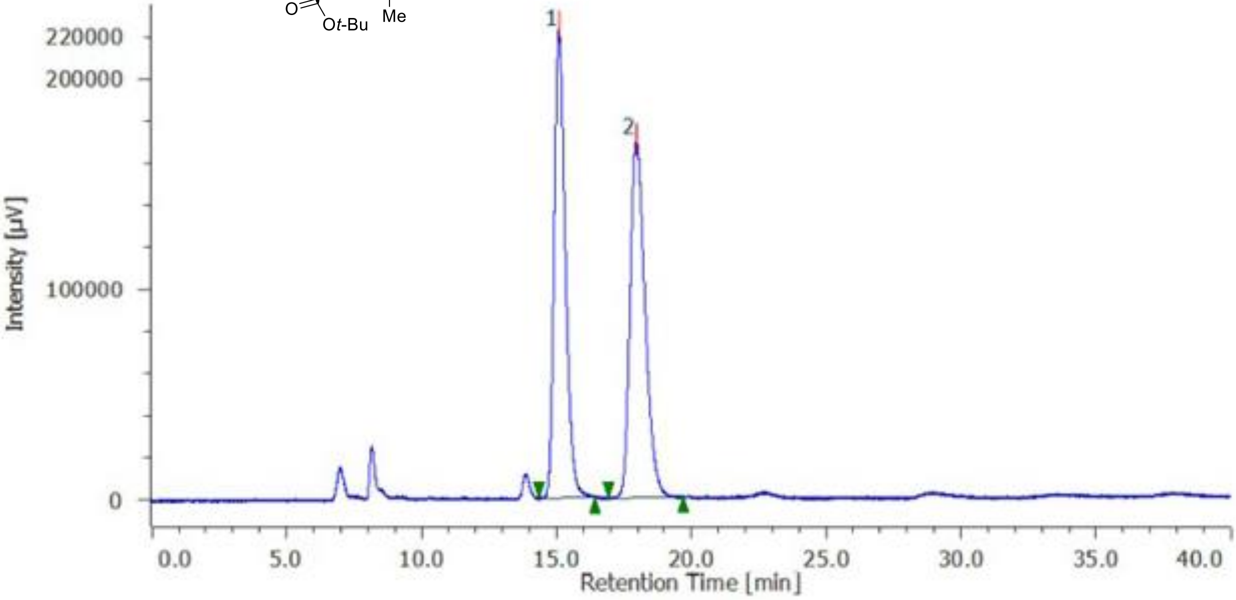

#	Peak Name	CH	tR [min]	Area [µV·sec]	Height [µV]	Area%	Height%	Quantity	NTP	Resolution	Symmetry Factor	Warning
1	Unknown	9	15.086	6392701	223027	49.569	56.779	N/A	6558	3.293	1.312	
2	Unknown	9	17.952	6503974	169770	50.431	43.221	N/A	5154	N/A	1.332	

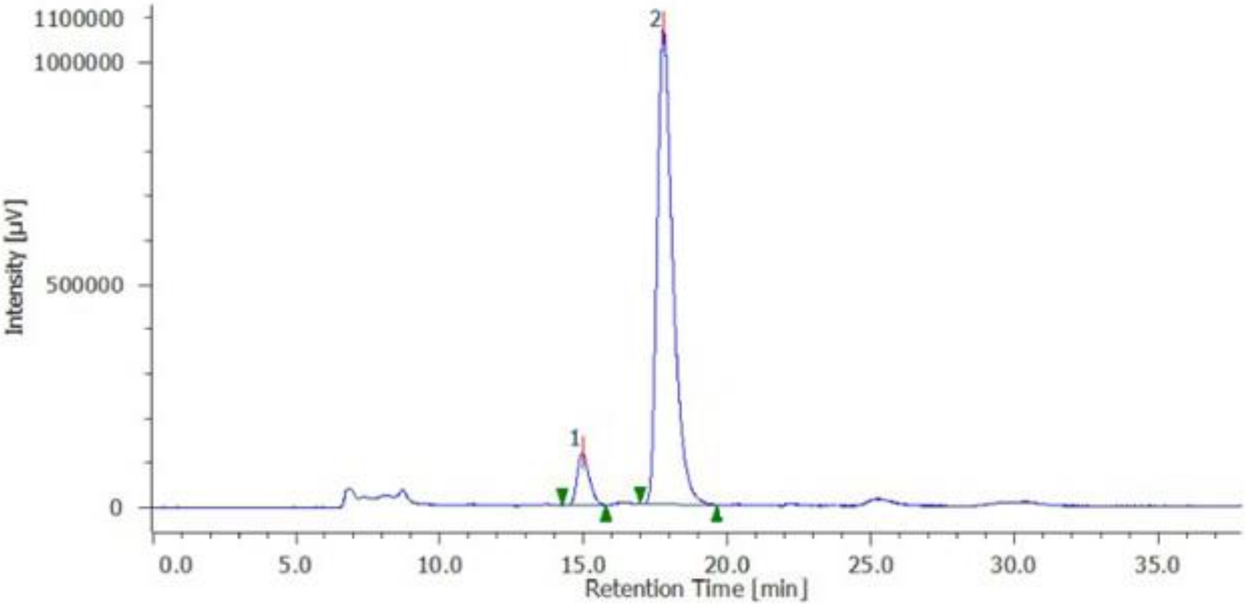

#	Peak Name	CH	tR [min]	Area [µV·sec]	Height [µV]	Area%	Height%	Quantity	NTP	Resolution	Symmetry Factor	Warning
1	Unknown	9	14.960	3453803	118676	7.861	9.997	N/A	6084	3.268	1.275	
2	Unknown	9	17.777	40479894	1068393	92.139	90.003	N/A	5467	N/A	1.514	

Compound 17ae

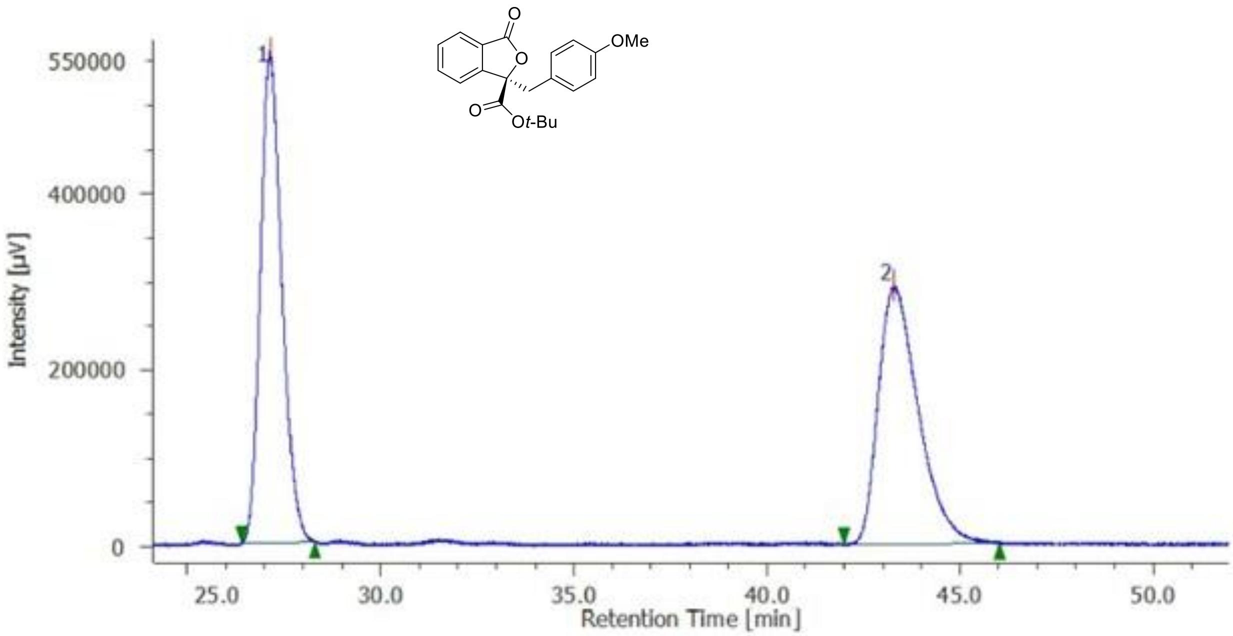

#	Peak Name	CH	tR [min]	Area [µV·sec]	Height [µV]	Area%	Height%	Quantity	NTP	Resolution	Symmetry Factor	Warning
1	Unknown	9	27.148	21434095	557572	50.516	65.561	N/A	11621	11.264	1.207	
2	Unknown	9	43.280	20996311	292893	49.484	34.439	N/A	8621	N/A	1.513	

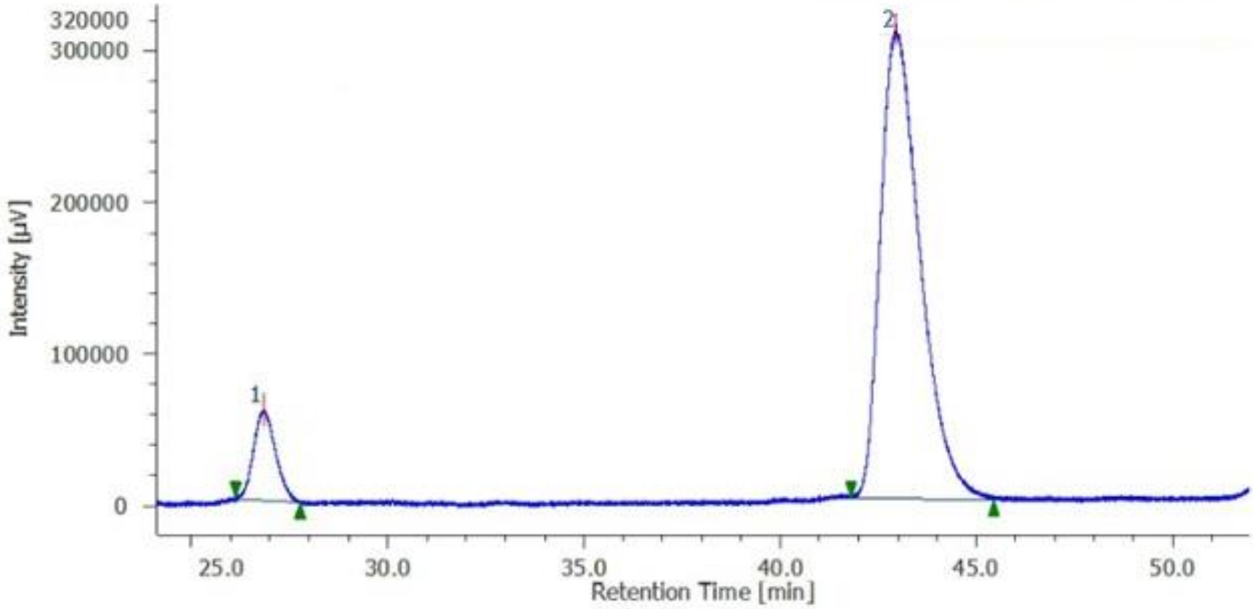

#	Peak Name	CH	tR [min]	Area [µV·sec]	Height [µV]	Area%	Height%	Quantity	NTP	Resolution	Symmetry Factor	Warning
1	Unknown	9	26.825	2303167	59829	9.597	16.183	N/A	10715	11.271	1.246	
2	Unknown	9	42.946	21696838	309880	90.403	83.817	N/A	8797	N/A	1.508	

Compound 17af

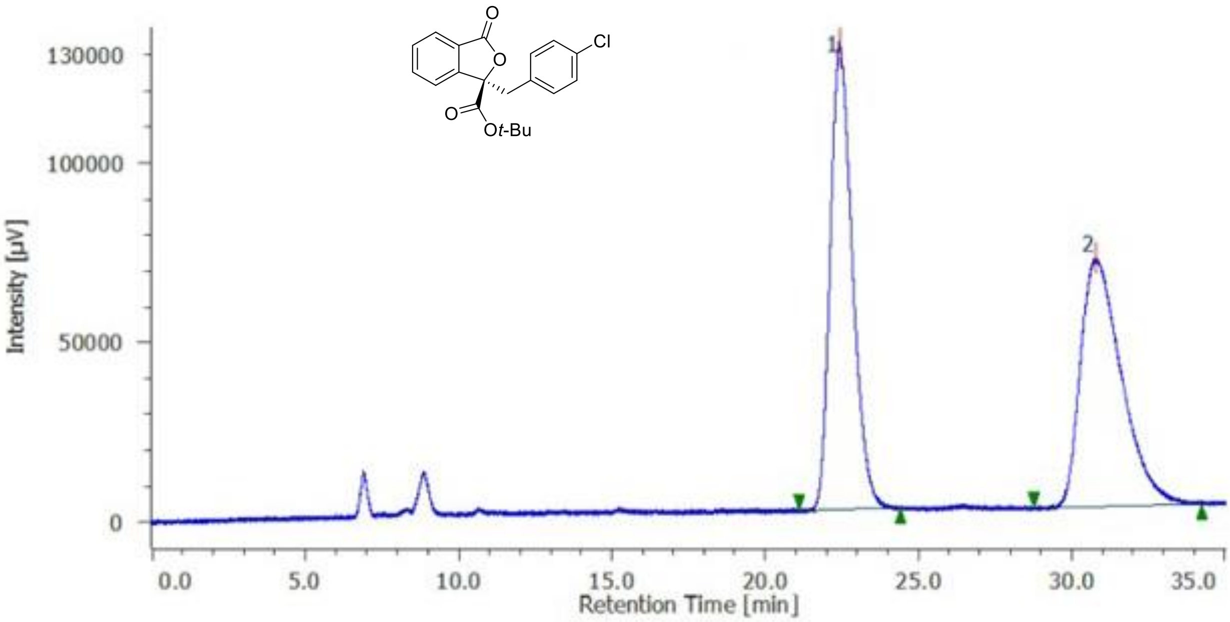

#	Peak Name	CH	tR [min]	Area [µV·sec]	Height [µV]	Area%	Height%	Quantity	NTP	Resolution	Symmetry Factor	Warning
1	Unknown	9	22.419	6478857	127795	50.285	64.895	N/A	4535	4.432	1.324	
2	Unknown	9	30.763	6405498	69132	49.715	35.105	N/A	2536	N/A	1.634	

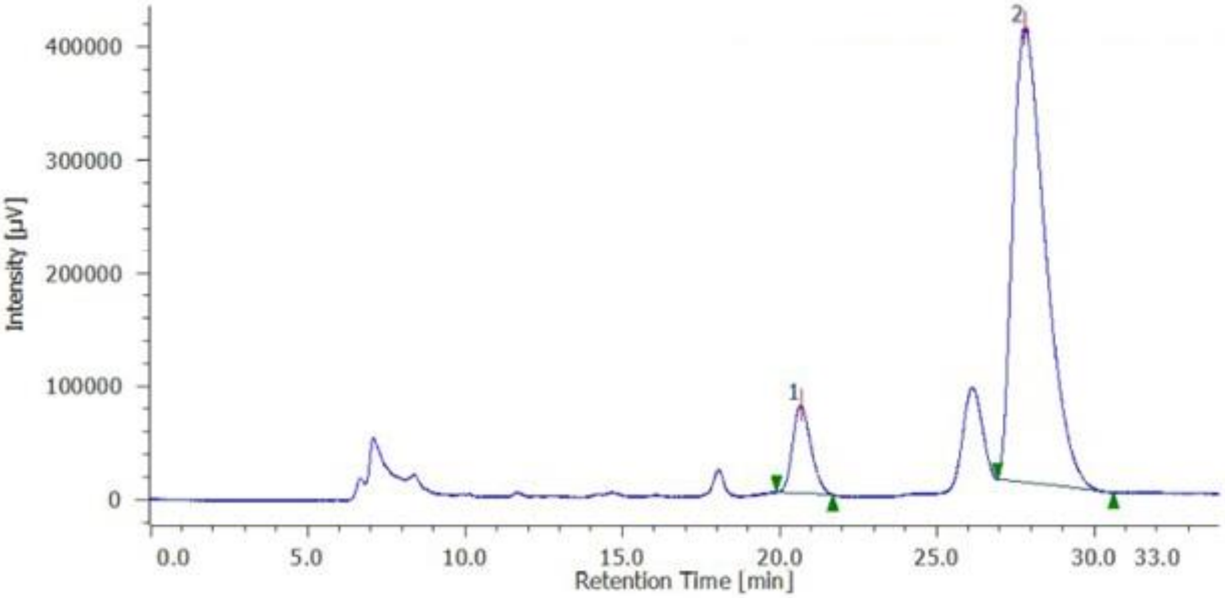

#	Peak Name	CH	tR [min]	Area [µV·sec]	Height [µV]	Area%	Height%	Quantity	NTP	Resolution	Symmetry Factor	Warning
1	Unknown	9	20.695	3110491	77293	9.783	16.194	N/A	5957	4.776	1.175	
2	Unknown	9	27.797	28685675	400012	90.217	83.806	N/A	3391	N/A	1.631	

Compound 17ag

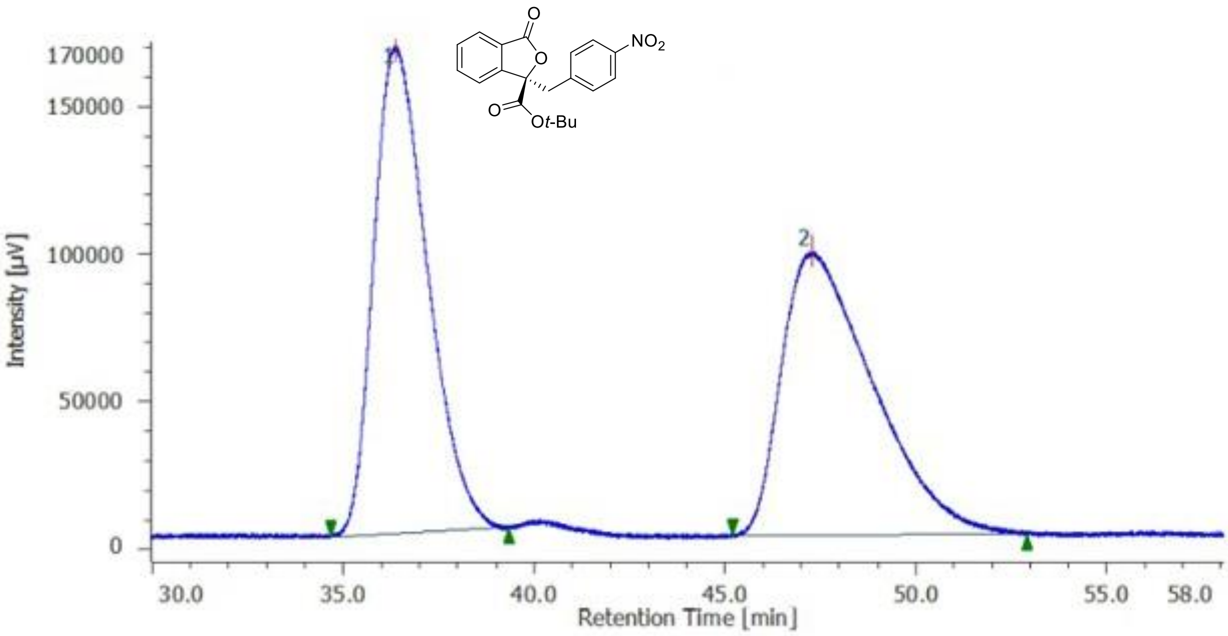

#	Peak Name	CH	tR [min]	Area [μV·sec]	Height [μV]	Area%	Height%	Quantity	NTP	Resolution	Symmetry Factor	Warning
1	Unknown	9	36.378	16132673	164985	50.340	63.191	N/A	3118	3.102	1.373	
2	Unknown	9	47.270	15914568	96105	49.660	36.809	N/A	1818	N/A	1.783	

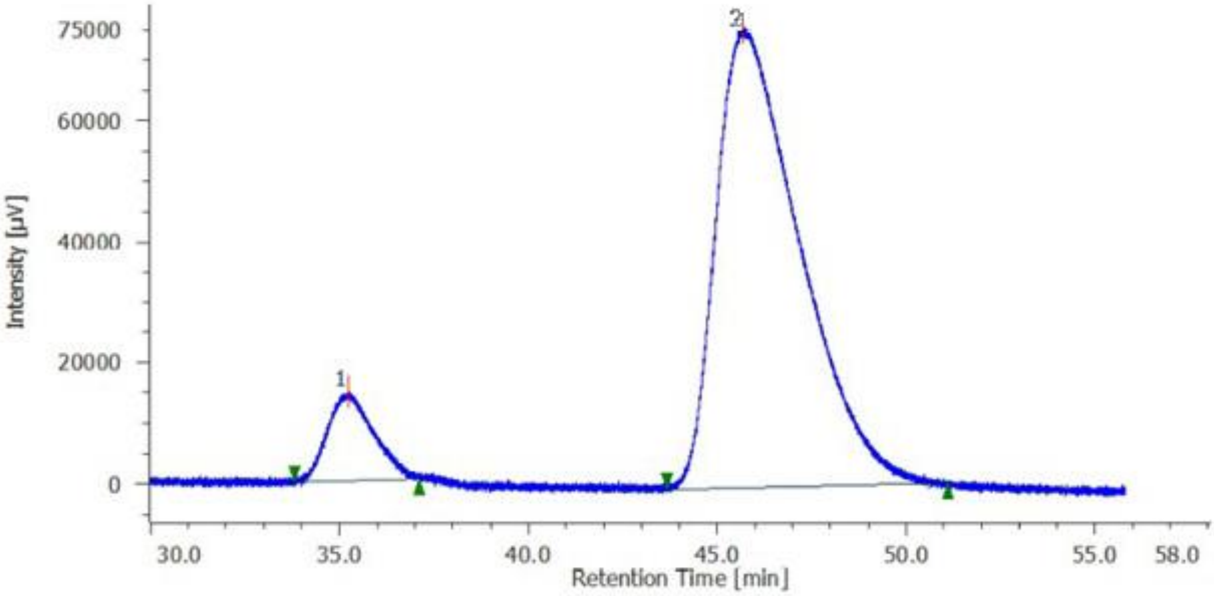

#	Peak Name	CH	tR [min]	Area [μV·sec]	Height [μV]	Area%	Height%	Quantity	NTP	Resolution	Symmetry Factor	Warning
1	Unknown	9	35.224	1205591	14576	9.385	16.087	N/A	3623	3.273	1.189	
2	Unknown	9	45.703	11640452	76028	90.615	83.913	N/A	2008	N/A	1.768	

Compound 17ah

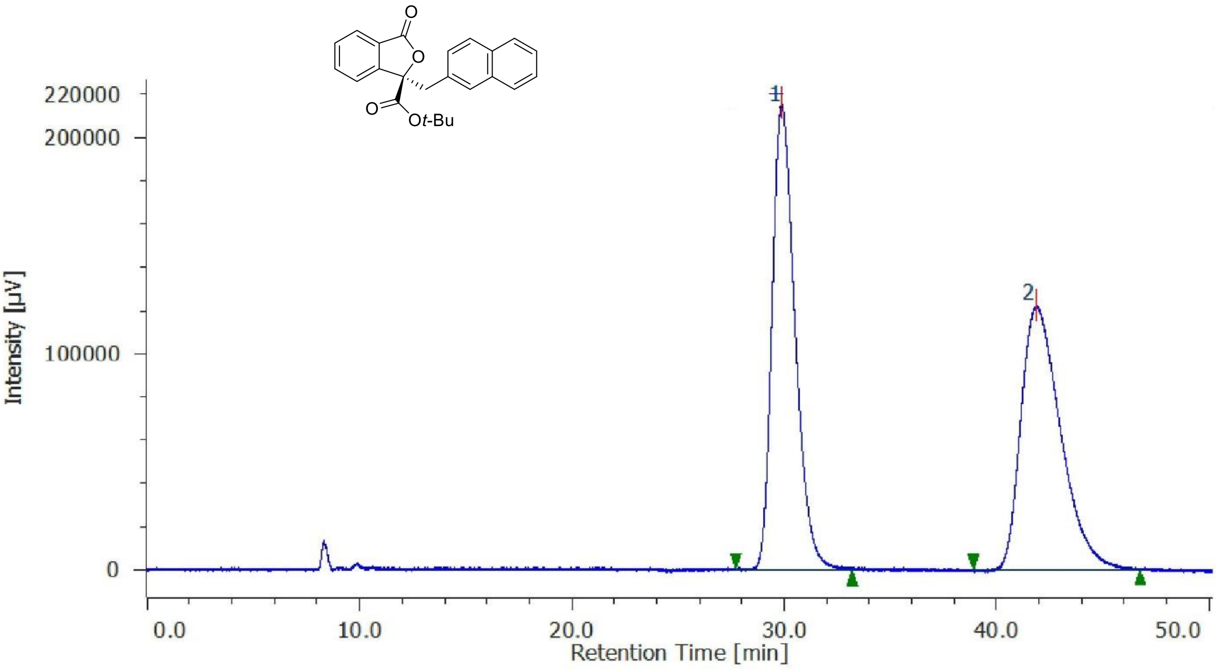

#	Peak Name	CH	tR [min]	Area [μV·sec]	Height [μV]	Area%	Height%	Quantity	NTP	Resolution	Symmetry Factor	Warning
1	Unknown	9	29.875	15955344	215614	50.464	63.792	N/A	3785	4.528	1.323	
2	Unknown	9	41.866	15661747	122380	49.536	36.208	N/A	2472	N/A	1.472	

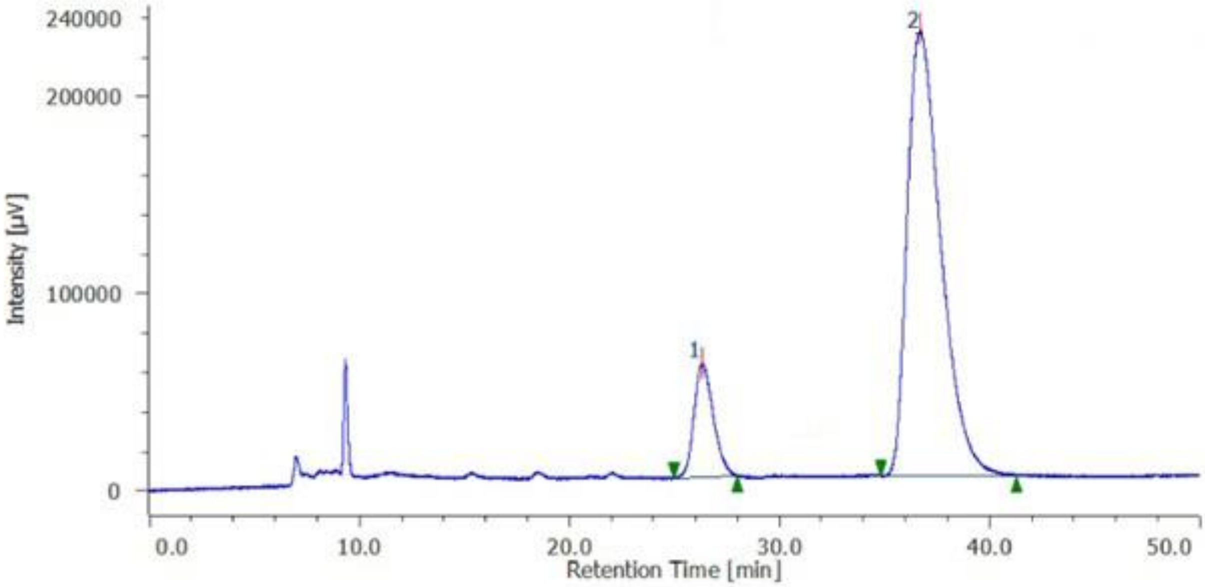

#	Peak Name	CH	tR [min]	Area [μV·sec]	Height [μV]	Area%	Height%	Quantity	NTP	Resolution	Symmetry Factor	Warning
1	Unknown	9	26.259	3716439	57933	12.879	20.330	N/A	3842	4.552	1.328	
2	Unknown	9	36.672	25140937	227030	87.121	79.670	N/A	2572	N/A	1.576	

Compound 17ai

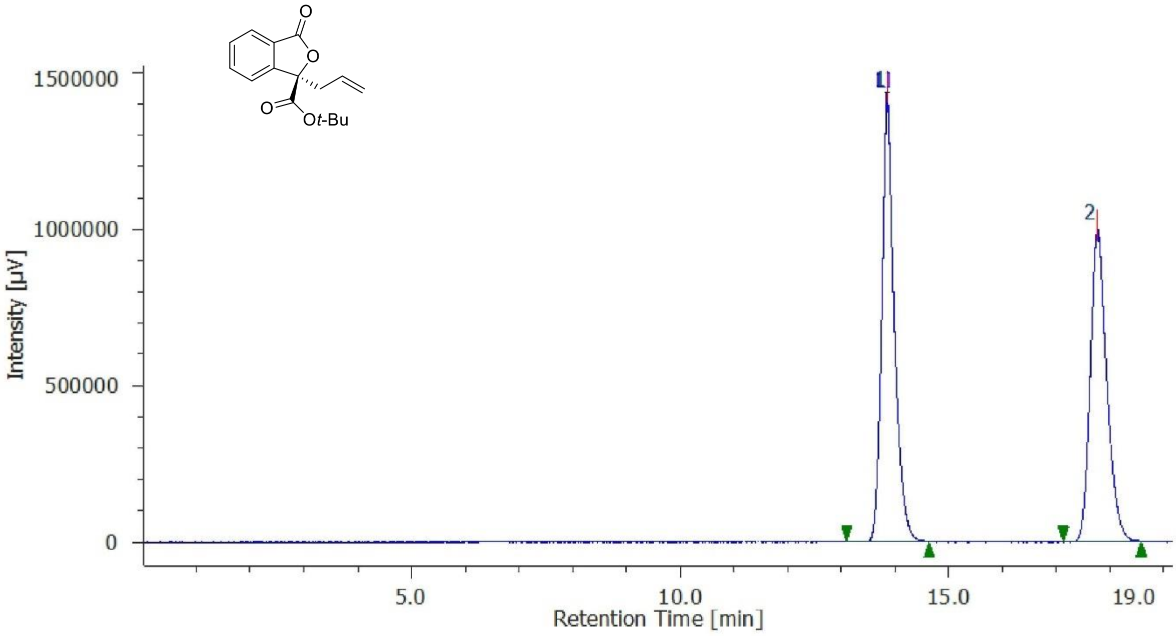

#	Peak Name	CH	tR [min]	Area [µV·sec]	Height [µV]	Area%	Height%	Quantity	NTP	Resolution	Symmetry Factor	Warning
1	Unknown	9	13.857	3768103	189111	49.711	54.221	N/A	11356	6.815	1.286	
2	Unknown	9	17.765	3811979	159670	50.289	45.779	N/A	12730	N/A	1.256	

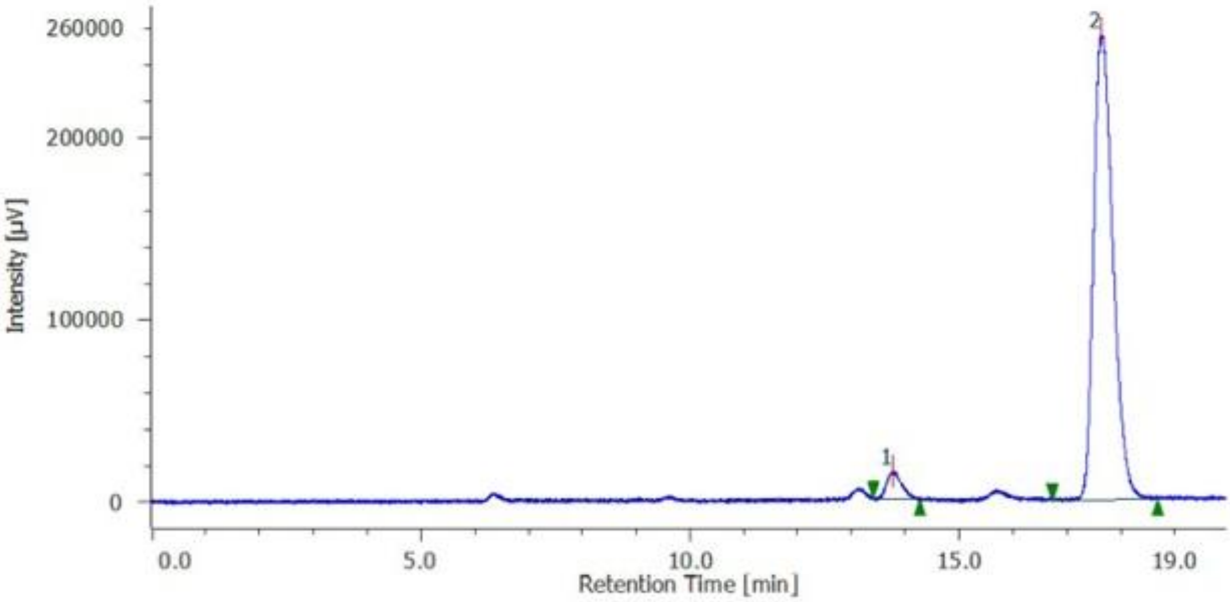

#	Peak Name	CH	tR [min]	Area [µV·sec]	Height [µV]	Area%	Height%	Quantity	NTP	Resolution	Symmetry Factor	Warning
1	Unknown	9	13.767	271583	15043	4.072	5.512	N/A	11459	6.622	1.432	
2	Unknown	9	17.635	6397294	257868	95.928	94.488	N/A	11532	N/A	1.356	

Compound 17ba

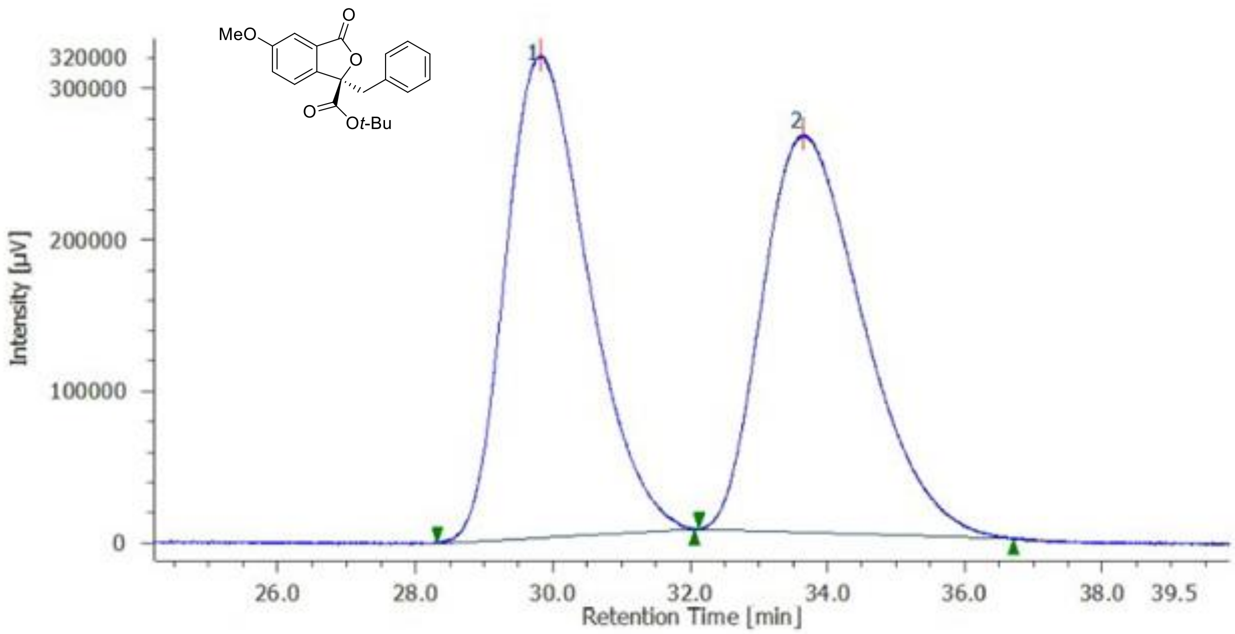

#	Peak Name	CH	tR [min]	Area [µV·sec]	Height [µV]	Area%	Height%	Quantity	NTP	Resolution	Symmetry Factor	Warning
1	Unknown	9	29.827	26544652	318315	49.818	54.791	N/A	2912	1.557	1.312	
2	Unknown	9	33.647	26738238	262651	50.182	45.209	N/A	2468	N/A	1.390	

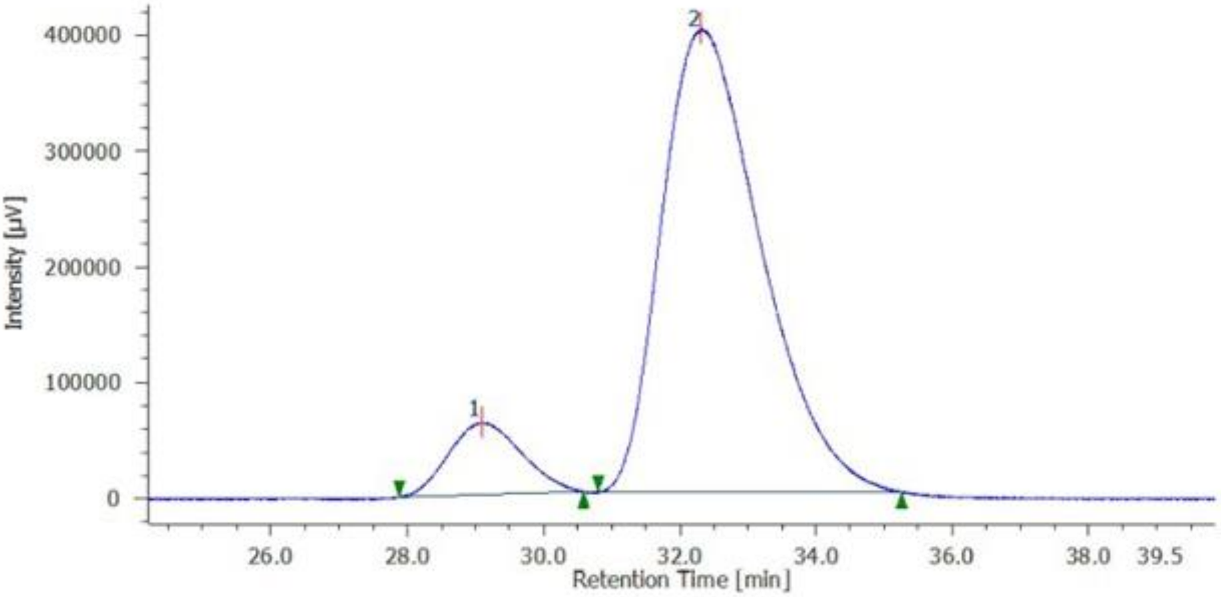

#	Peak Name	CH	tR [min]	Area [µV·sec]	Height [µV]	Area%	Height%	Quantity	NTP	Resolution	Symmetry Factor	Warning
1	Unknown	9	29.101	4590636	62134	10.211	13.433	N/A	3258	1.362	1.139	
2	Unknown	9	32.308	40364935	400407	89.789	86.567	N/A	2323	N/A	1.446	

Compound 17ca

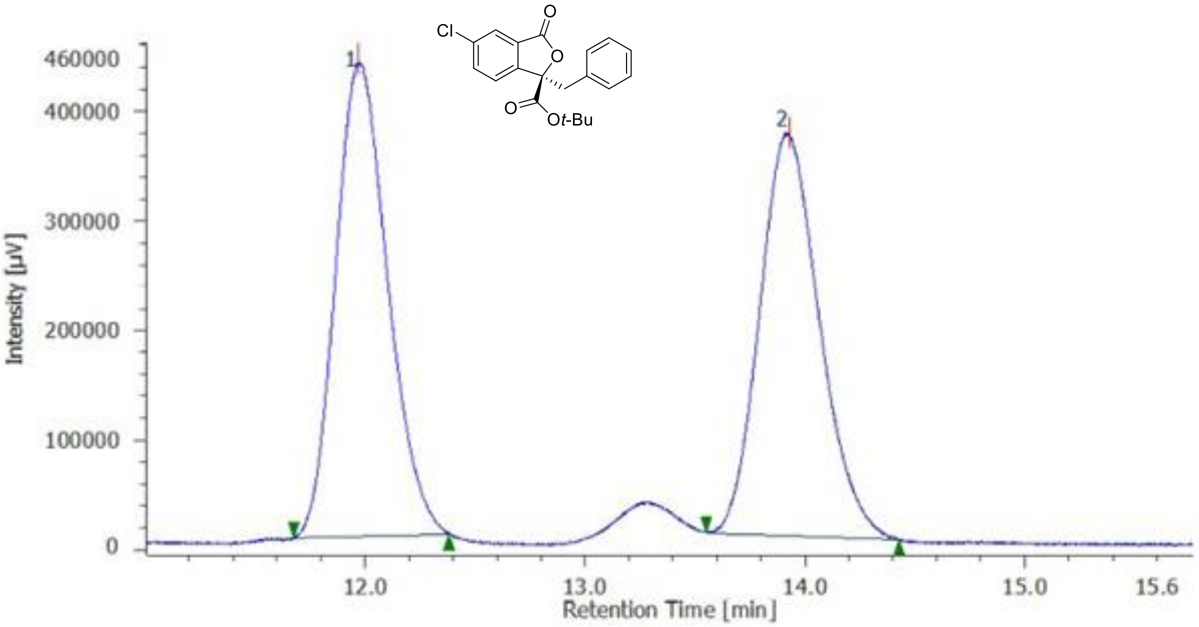

#	Peak Name	CH	tR [min]	Area [µV·sec]	Height [µV]	Area%	Height%	Quantity	NTP	Resolution	Symmetry Factor	Warning
1	Unknown	9	11.970	7103442	435200	50.603	54.213	N/A	12284	4.230	1.218	
2	Unknown	9	13.928	6934263	367562	49.397	45.787	N/A	12590	N/A	1.136	

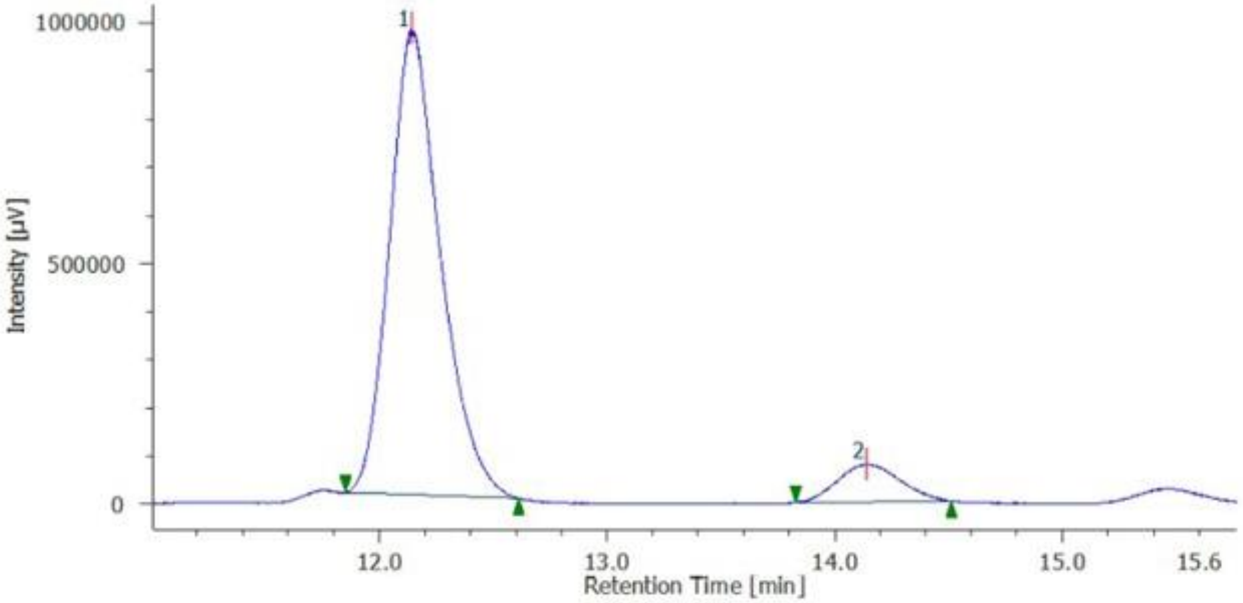

#	Peak Name	CH	tR [min]	Area [µV·sec]	Height [µV]	Area%	Height%	Quantity	NTP	Resolution	Symmetry Factor	Warning
1	Unknown	9	12.139	15007743	968147	91.060	92.450	N/A	14891	4.406	1.280	
2	Unknown	9	14.137	1473401	79060	8.940	7.550	N/A	12219	N/A	1.113	

Compound 17da

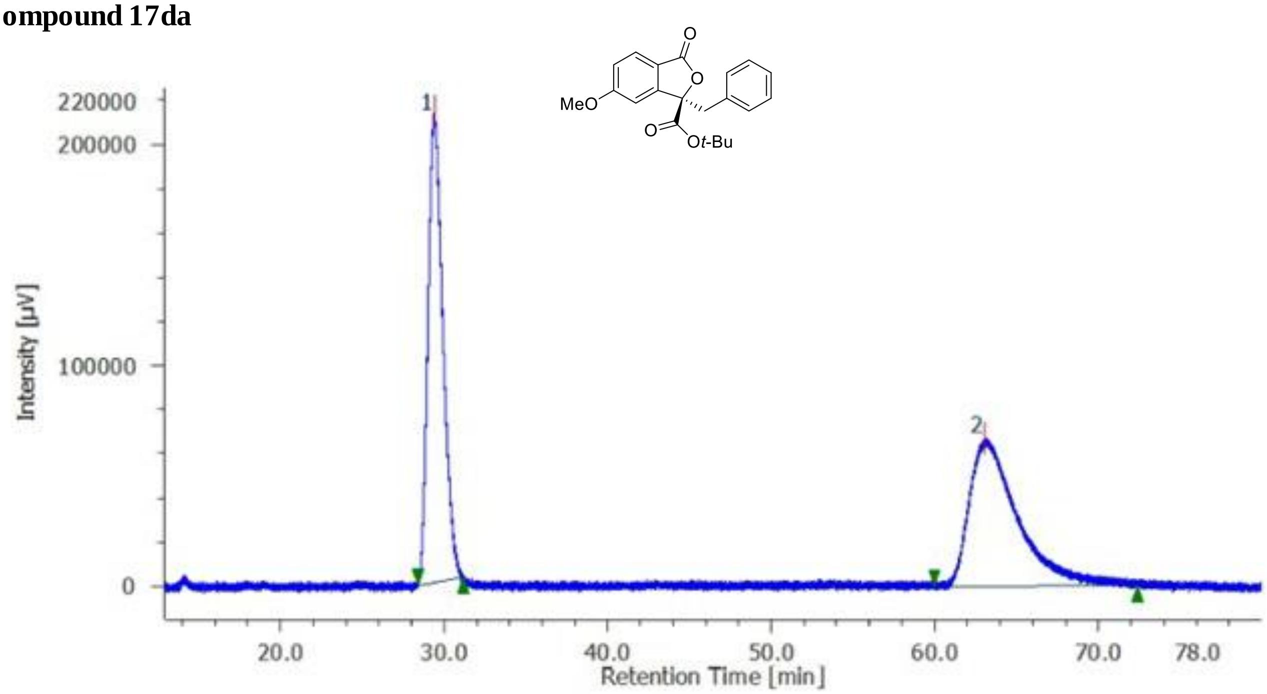

#	Peak Name	CH	tR [min]	Area [µV·sec]	Height [µV]	Area%	Height%	Quantity	NTP	Resolution	Symmetry Factor	Warning
1	Unknown	9	29.412	13460340	213088	49.761	76.195	N/A	4903	9.887	1.298	
2	Unknown	9	63.069	13589871	66572	50.239	23.805	N/A	2403	N/A	2.469	

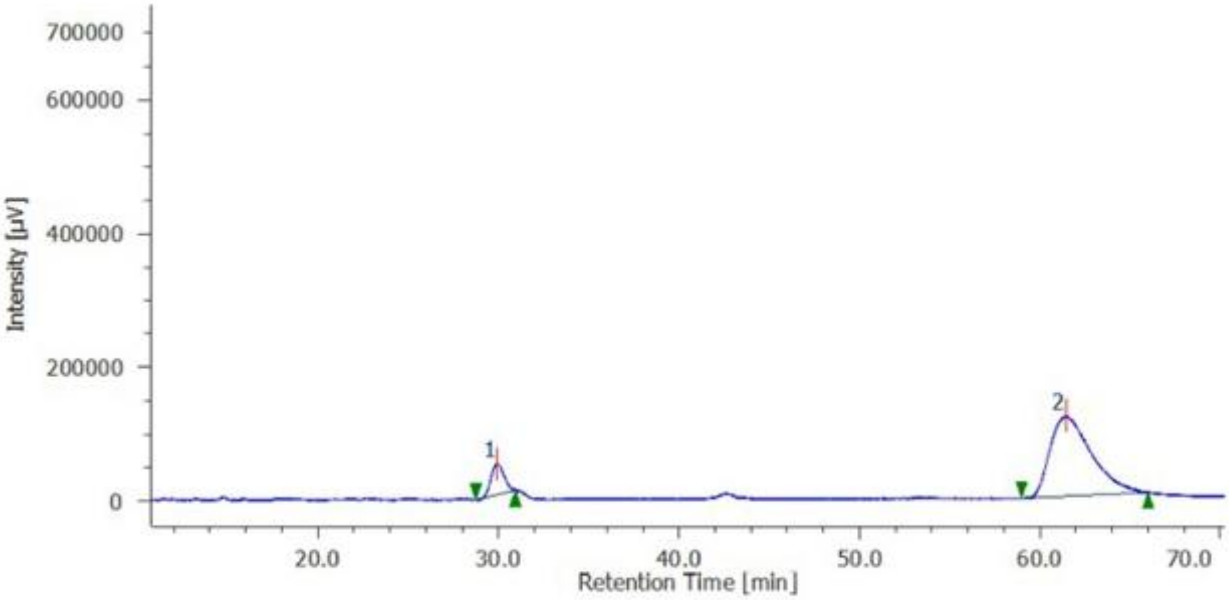

#	Peak Name	CH	tR [min]	Area [µV·sec]	Height [µV]	Area%	Height%	Quantity	NTP	Resolution	Symmetry Factor	Warning
1	Unknown	9	29.908	2251747	46058	10.735	27.777	N/A	7671	11.447	1.156	
2	Unknown	9	61.413	18724384	119755	89.265	72.223	N/A	3499	N/A	1.624	

Compound 17ea

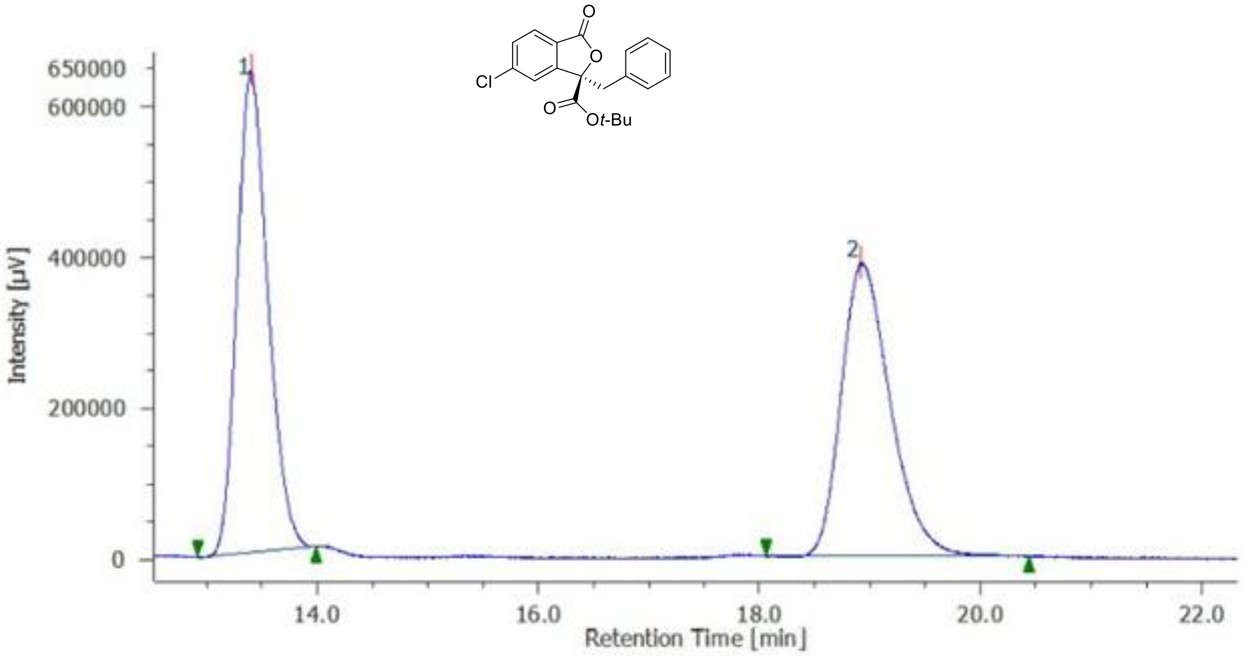

#	Peak Name	CH	tR [min]	Area [µV·sec]	Height [µV]	Area%	Height%	Quantity	NTP	Resolution	Symmetry Factor	Warning
1	Unknown	9	13.407	12331775	637644	50.837	62.140	N/A	11022	8.446	1.195	
2	Unknown	9	18.915	11925621	388496	49.163	37.860	N/A	9012	N/A	1.373	

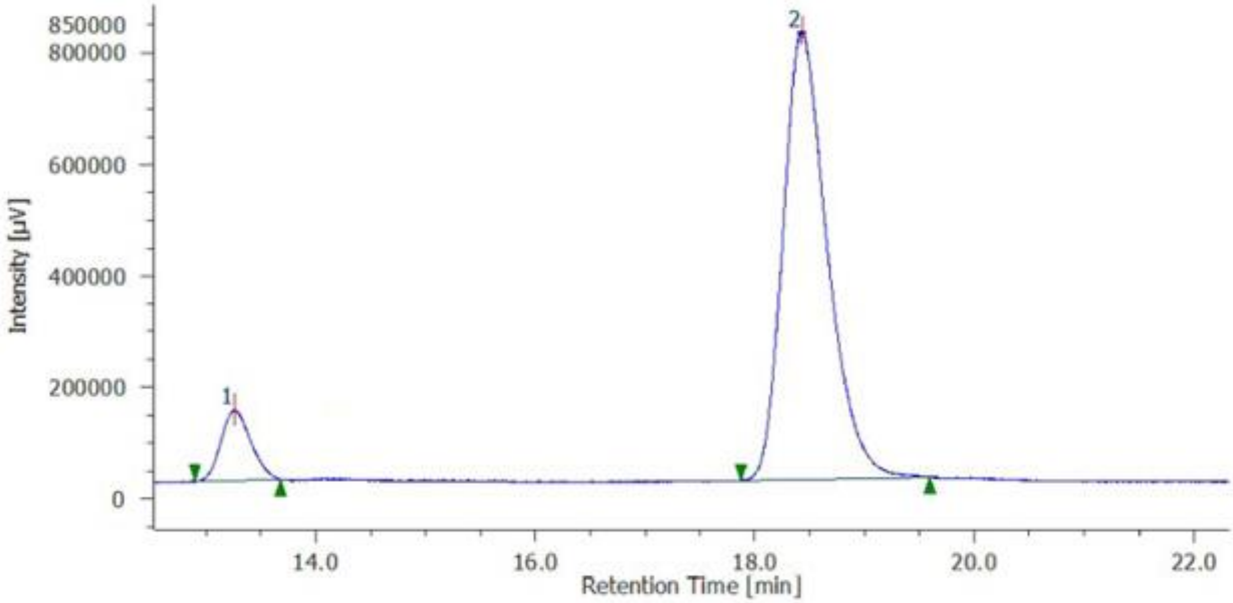

#	Peak Name	CH	tR [min]	Area [µV·sec]	Height [µV]	Area%	Height%	Quantity	NTP	Resolution	Symmetry Factor	Warning
1	Unknown	9	13.256	2374723	125859	9.544	13.459	N/A	11076	8.487	1.193	
2	Unknown	9	18.425	22506716	809275	90.456	86.541	N/A	10545	N/A	1.337	

Compound 18

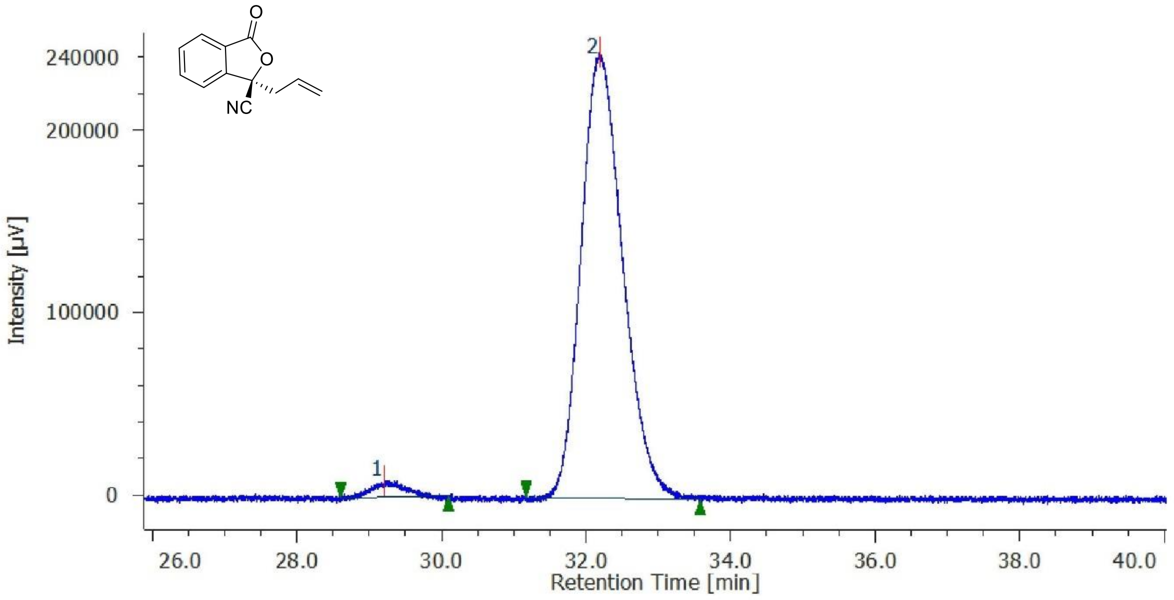

#	Peak Name	CH	tR [min]	Area [µV·sec]	Height [µV]	Area%	Height%	Quantity	NTP	Resolution	Symmetry Factor	Warning
1	Unknown	9	29.210	249103	8880	2.489	3.507	N/A	12648	2.851	1.141	
2	Unknown	9	32.185	9758407	244351	97.511	96.493	N/A	14941	N/A	1.228	

Compound 19

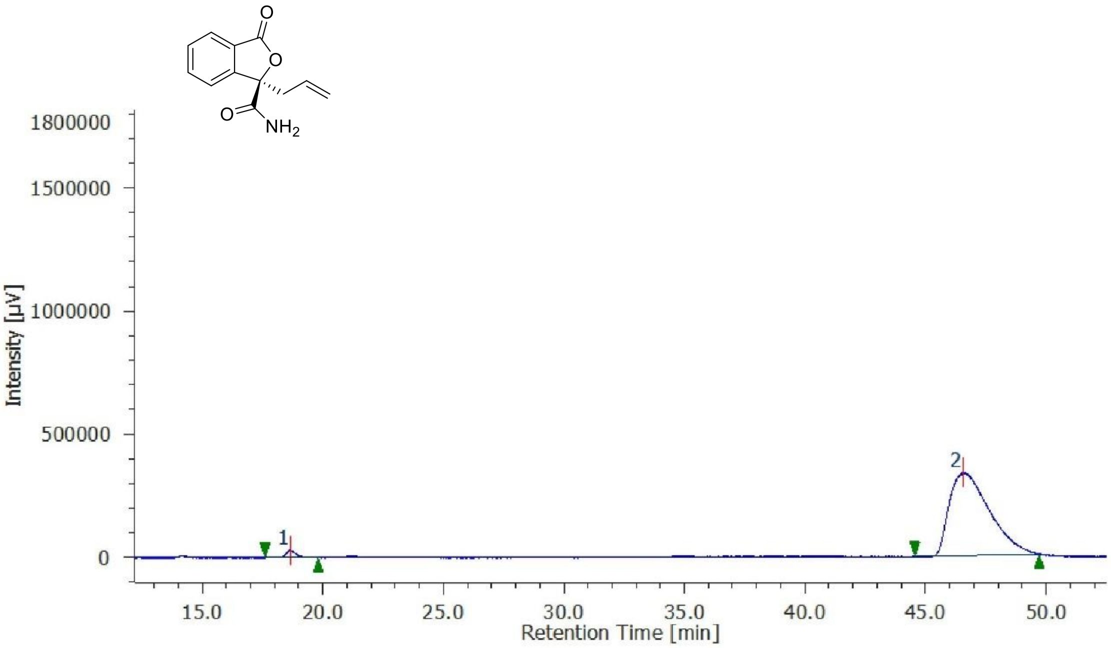

#	Peak Name	CH	tR [min]	Area [µV·sec]	Height [µV]	Area%	Height%	Quantity	NTP	Resolution	Symmetry Factor	Warning
1	Unknown	9	18.652	888052	30123	2.256	8.199	N/A	9857	14.518	1.066	
2	Unknown	9	46.554	38469705	337279	97.744	91.801	N/A	3602	N/A	1.785	

Copies of HPLC traces after recrystallization

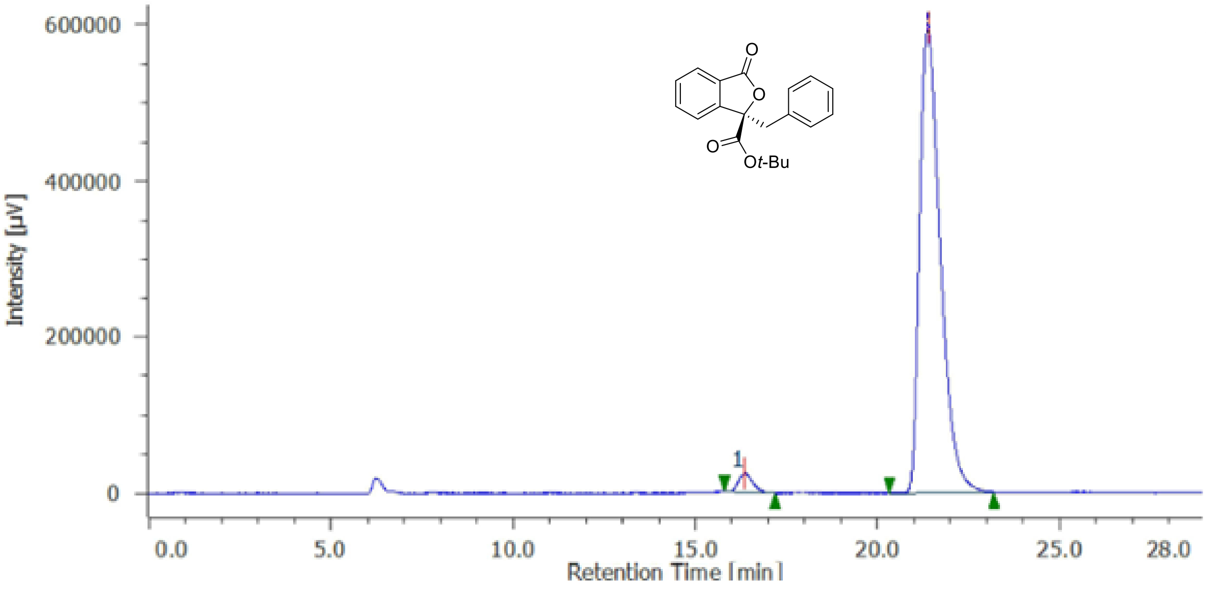

#	Peak Name	CH	tR [min]	Area [μV·sec]	Height [μV]	Area%	Height%	Quantity	NTP	Resolution	Symmetry Factor	Warning
1	Unknown	9	16.357	653866	24450	2.712	3.947	N/A	8853	5.852	1.238	
2	Unknown	9	21.384	23453704	595077	97.288	96.053	N/A	6935	N/A	1.515	

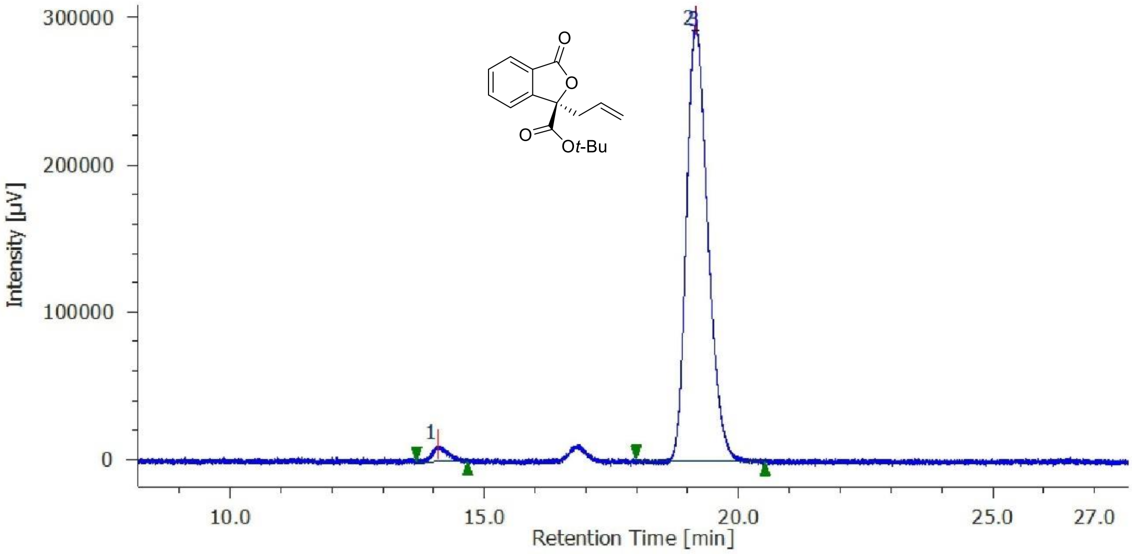

#	Peak Name	CH	tR [min]	Area [μV·sec]	Height [μV]	Area%	Height%	Quantity	NTP	Resolution	Symmetry Factor	Warning
1	Unknown	9	14.086	216971	11425	2.468	3.696	N/A	8988	7.523	1.167	
2	Unknown	9	19.156	8574805	297660	97.532	96.304	N/A	10242	N/A	1.276	
